# In vitro studies of the protein-interaction network of cell-wall lytic transglycosylase RlpA of *Pseudomonas aeruginosa*

**DOI:** 10.1038/s42003-022-04230-x

**Published:** 2022-11-30

**Authors:** Luis F. Avila-Cobian, Stefania De Benedetti, Choon Kim, Rhona Feltzer, Matthew M. Champion, Jed F. Fisher, Shahriar Mobashery

**Affiliations:** Department of Chemistry and Biochemistry, University of Notre Dame, Notre Dame, IN 46556 USA

**Keywords:** Enzymes, Diseases

## Abstract

The protein networks of cell-wall-biosynthesis assemblies are largely unknown. A key class of enzymes in these assemblies is the lytic transglycosylases (LTs), of which eleven exist in *P. aeruginosa*. We have undertaken a pulldown strategy in conjunction with mass-spectrometry-based proteomics to identify the putative binding partners for the eleven LTs of *P. aeruginosa*. A total of 71 putative binding partners were identified for the eleven LTs. A systematic assessment of the binding partners of the rare lipoprotein A (RlpA), one of the pseudomonal LTs, was made. This 37-kDa lipoprotein is involved in bacterial daughter-cell separation by an unknown process. RlpA participates in both the multi-protein and multi-enzyme divisome and elongasome assemblies. We reveal an extensive protein-interaction network for RlpA involving at least 19 proteins. Their kinetic parameters for interaction with RlpA were assessed by microscale thermophoresis, surface-plasmon resonance, and isothermal-titration calorimetry. Notable RlpA binding partners include PBP1b, PBP4, and SltB1. Elucidation of the protein-interaction networks for each of the LTs, and specifically for RlpA, opens opportunities for the study of their roles in the complex protein assemblies intimately involved with the cell wall as a structural edifice critical for bacterial survival.

## Abstract (web summary)

An extensive protein-interaction network of cell-wall biosynthesis assemblies in *P. aeruginosa* is elucidated using a pulldown strategy with mass-spectrometry-based proteomics and binding assays.

## Introduction

The bacterial cell wall is a polymer of crosslinked glycan strands with repeating β-(1→4)-*N*-acetylglucosamine (NAG)-*N*-acetyl muramic acid (NAM) disaccharide. A structurally unique stem peptide—often with a pentapeptide l-Ala-γ-d-Glu-*m*-DAP-d-Ala-d-Ala (where DAP is diaminopimelate)—is appended to the NAM saccharide. The stem from one glycan strand is crosslinked to that of a neighboring strand^1–5^. This cell wall polymer encases the cytoplasmic membrane and provides structural integrity to the bacterium. Several dozen enzymes are involved in the biosynthesis, turnover, and overall homeostasis of the cell wall. The functions of many of these enzymes—located both in the cytoplasm and in the periplasm—are critical. These functions are coordinated with the metabolic pathways and the cell cycle of the bacterium. While the availability of whole-genome sequences for bacteria (with the associated bioinformatic analyses and annotations) gives one context for the mechanistic study of these processes, the resulting experiments often focus on an individual gene and its corresponding individual protein. In reality, these proteins rarely function in isolation^6^. Two multi-protein entities are the elongasome and divisome. In rod-shaped bacteria, the elongasome synthesizes sidewall peptidoglycan and the divisome synthesizes septal peptidoglycan^7–19^. Each of these entities interconnects proteins within the cytoplasm, within the cytoplasmic membrane, within the periplasm, and within the outer membrane. The identification of the proteins in these assemblies is often based on the spatiotemporal convergence of chromophore-labeled proteins to specific subsites, guided by mechanistic intuition. How the protein–protein interactions coordinate is the current mechanistic frontier for the understanding of cell-wall biosynthesis^6,20–25^.

**Fig. 1:**
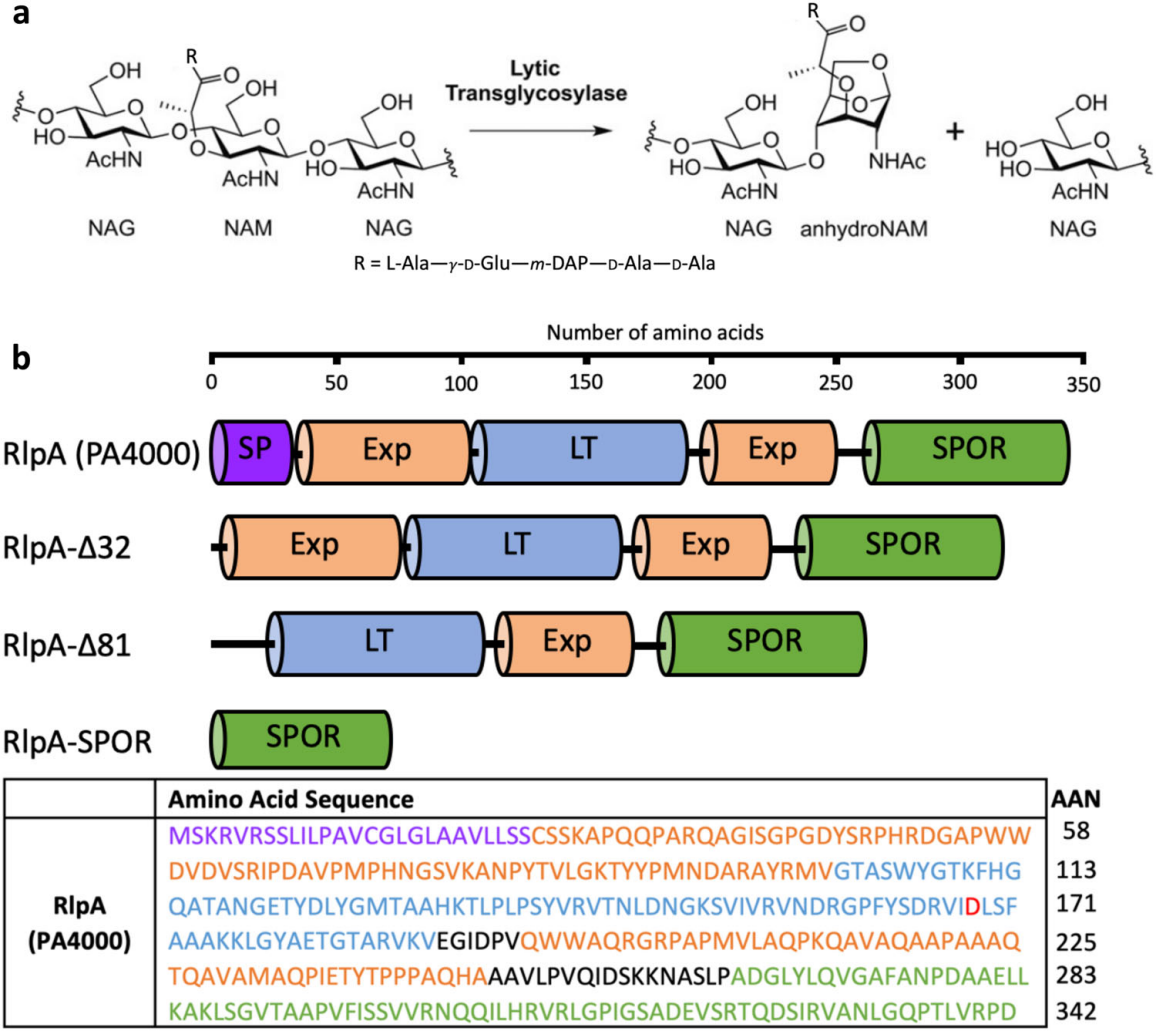
Schematic representations of LT reaction, RlpA constructs, and primary structure of RlpA. **a** LTs catalyze the non-hydrolytic cleavage of the NAM-NAG glycosidic bond. NAG denotes *N*-acetylglucosamine and NAM denotes *N*-acetyl muramic acid. While a peptide stem is attached ordinarily to the lactyl of the NAM unit (R = peptide stem), RlpA acts on lactyl-unsubstituted, or denuded, peptidoglycan (R is oxygen, giving a carboxylate functional group). **b** Primary structure is shown for RlpA (Pseudomonas Genome Database, Cystic Fibrosis Foundation, Therapeutics). “SP” in purple denotes *N*-terminal signal peptide; amino acids 1–26. The linker sequence (amino acids 27–81) is colored orange to reflect its probable identity as part of the expansin domain. “LT” in blue denotes lytic transglycosylase domain: amino acids 102–189. “Exp” in orange denotes expansin-type domain: amino acids 196–246. “SPOR” in green denotes peptidoglycan-binding SPOR domain: amino acids 264–342. Catalytic aspartate in the LT domain is highlighted red: amino acid 168. AAN denotes the number of amino acids in each line of the chart.

A focus of our laboratory is the structural and mechanistic relationship of the lytic transglycosylases (LTs) of the bacterium *Pseudomonas aeruginosa*. The LTs are a family of enzymes that turn over the cell wall. *P. aeruginosa* is typical of many Gram-negative bacteria. It has a family of eleven LT enzymes. Four are soluble enzymes of the periplasm, and seven are lipoproteins fixed to the inner leaflet of the outer membrane by covalent attachment to the lipid of that leaflet, extending into the periplasm. The LT enzymes catalyze the non-hydrolytic cleavage of the NAM-NAG glycosidic bond of the repeating—[NAG-NAM]_n_—a structure of the peptidoglycan strand^26–28^. The LT reaction creates a distinctive anhydroNAM saccharide glycan terminus (Fig. 1a). One LT, the MltG, terminates glycan strand lengthening in peptidoglycan biosynthesis^29–32^. In the absence of MltG, other LTs assume this function. Extensive redundancy of LT function is recognized^33^. While an overall capacity for LT processing is now understood as essential to peptidoglycan integrity, the individual roles of each member of the LT family are not understood. Contributing to this uncertainty is the emerging realization that an LT of one bacterial species may not have the same role as an ortholog found in a different bacterial species^34,35^. A pragmatic approach in this circumstance is to prioritize members of the LT family that are demonstrated as important and/or correlate to antibiotic efficacy, and to identify their protein interactions. For *P. aeruginosa*, the LTs of its family that meets these criteria are the lipoproteins RlpA, MltD, and MltG; and the soluble Slt^27,36,37^.

RlpA is a compelling choice—as assessed both as a protein and as an enzyme—for the study of LT function. This protein is a structural component of the divisome within *Escherichia coli*, and is without catalytic LT activity^38^. In contrast, RlpA of *P. aeruginosa* is both a structural protein and an LT catalyst. RlpA is found in discrete locations in the sidewall peptidoglycan, and at the septal glycan. RlpA is a three-domain protein: an expansin domain, an LT domain, and a peptidoglycan-binding sporulation-related repeat (SPOR) *C*-terminus domain^39^. The catalytic activity of RlpA in turning over the peptidoglycan is documented^33,38,40^. The sequence of this LT domain classifies it as that of a Family 2 LT showing a GH45 motif. Gram-negative bacteria typically have a second GH45 LT, MltA. MltA structures have two domains, an LT and an expansin. Short linkers connect the LT domain to the expansin domain^41,42^. MltA of *E. coli* forms a stable heterotrimer with two proteins: one is a Penicillin-Binding-Protein (PBP) of peptidoglycan synthesis (PBP1b), and the other is the MipA transmembrane scaffolding protein^43^. As we shall see, PBP1b is also a partner of RlpA.

Expansin domains are glycoside-strand-binding domains^44–46^. The identification of the central domain of RlpA as an expansin domain is evident from two sequence motifs (…XXDLS… and …WWA…) also found *inter alia* in the sequence of the *Bacillus subtilis* EXLX1 expansin protein^46–48^. The third domain of RlpA is SPOR domain^39^. The well-studied Gram-negative bacterium *E. coli* has four SPOR domain-containing proteins (DamX, DedD, FtsN, and RlpA)^49,50^. The SPOR domain is a peptidoglycan-binding motif that recognizes the “stem-denuded” peptidoglycan found at the division site of the bacterium^51–53^. Here “denuded” refers to the amidase-catalyzed loss of the stem peptide crosslinkers of the peptidoglycan. FtsN is the best-studied SPOR-containing protein. It is a bitopic membrane protein, which projects its SPOR domain into the periplasm for contact with denuded peptidoglycan. FtsN is proposed as the key regulatory protein whose recruitment activates the divisome for septal peptidoglycan biosynthesis^54–56^. The DedD and DamX SPOR proteins of *E. coli* regulate the catalytic activity of the two PBPs of the divisome (PBP1a and PBP1b, respectively). Deletion of DedD or DamX gives septal defects^57,58^. *E. coli* RlpA lacks LT activity (due to the substitution of the catalytic aspartate with a serine) and is bound to the large (1329 amino acids), polytopic membrane protein FtsK of the divisome^38,59^. FtsK coordinates the essential function of chromosome segregation with cell division, and in this task, may structurally connect the cytoskeletal proteins to proteins of the divisome located in the periplasm, including the membrane-bound PBPs^60,61^. Deletion of RlpA from *E. coli* does not, however, give a recognizable phenotype^49^. In contrast, the RlpA of *P. aeruginosa* has in vitro activity as an LT and its loss of function of RlpA in *P. aeruginosa* gives a phenotype of short, fat cell chains, as a result of defective daughter-cell separation, when the bacterium is grown in low-osmotic strength media^38^. A similar phenotype results from loss of RlpA function in *Vibrio cholerae*^34,40^, and multiple LT, including RlpA, genetic deletion strains^53^. These observations indicate an important contribution of the LTs, and RlpA in particular, to cell shape and to daughter-cell separation. We surmise that denuding the mid-cell peptidoglycan of *P. aeruginosa* recruits the four SPOR domain-containing proteins and positions the four within a now-functioning divisome assembly. RlpA is the only enzyme of the four. Its three interconnected domains offer an extensive opportunity for protein–protein interaction. Here, we report our efforts toward an assessment of the protein-interaction network of *P. aeruginosa* RlpA. We document that RlpA binds to as many as 19 proteins, none of which were known previously as partners of this LT.

## Results

### Pulldown identification of LT-interacting partners

**Fig. 2:**
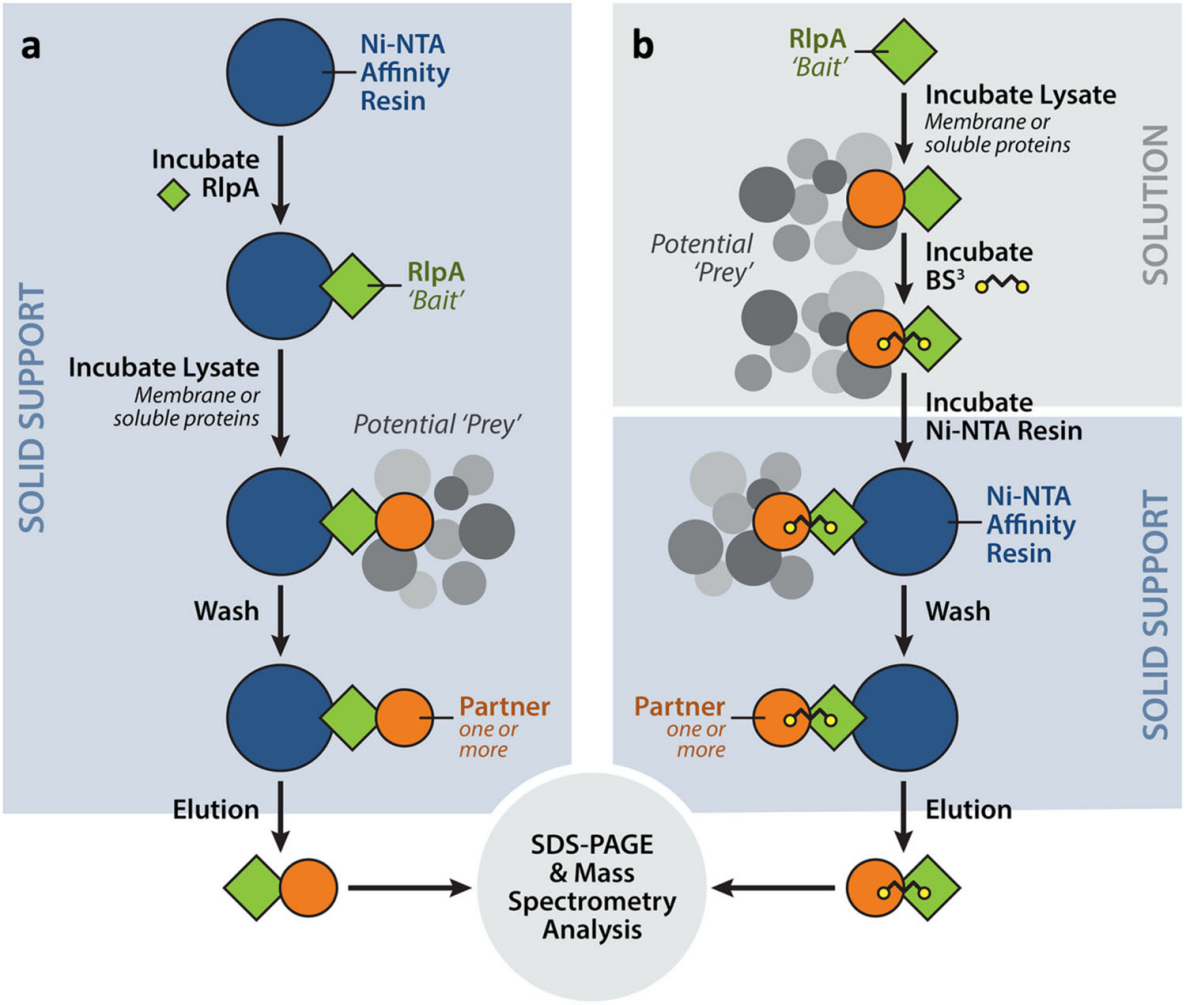
RlpA-Δ32 pulldown-enrichment strategy. **a** Pulldown without cross-linking and **b** with cross-linking. Gray and black shapes denote the *P. aeruginosa* proteome. Black chain with yellow-circle ends denotes BS^3^ cross-linking between “bait” (RlpA) and “prey” (from the proteome). “Prey” protein denotes any available protein binding to “bait”.

We performed pull-down enrichment experiments with LTs, as depicted in Fig. 2. *N*-terminal His-tagged constructs were done on each of the eleven LTs and were used as “bait” for the identification of its interacting partners. Figure 1b, Supplementary Figs. 1, 2, and Supplementary Table 1 display schematic representations, primary structures, and additional information for the construction of each of the eleven LTs. Ni-NTA resin was charged with His-tagged LT to immobilize the LT protein on the solid support. Portions of the resin were added separately to a solution of the soluble *P. aeruginosa* proteome and to a suspension of a solubilized membrane (0.5% NP-40) proteome fraction. Incubation with the bait resin was done by gentle agitation at 4 °C overnight. The suspension was centrifuged, decanted, the resin was washed, and the retained proteins were eluted from the resin by buffer supplemented with 500 mM imidazole. Two controls were performed in parallel: Ni-NTA resin without the LT protein and Ni-NTA resin with the LT protein but not exposed to the proteome solutions. Each elution was analyzed by mass spectrometry (Supplementary Tables 2, 3) for identification of the putative interacting partner proteins.

**Fig. 3:**
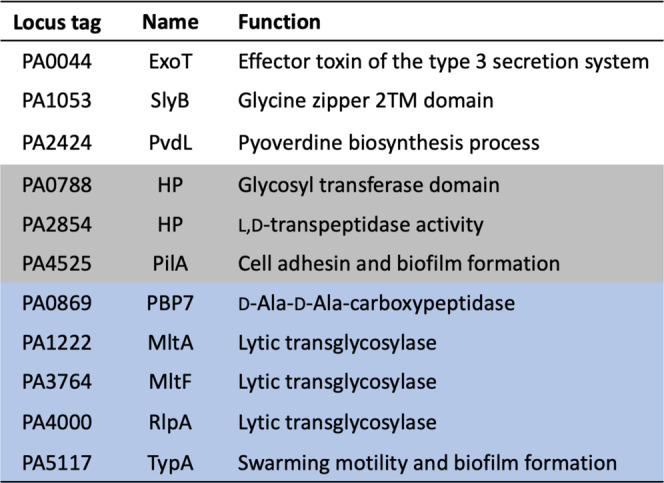
Select putative binding partners from the eleven LT pulldowns. Binding partners devoid of highlighted color were identified solely in the RlpA pulldown. Binding partners highlighted in gray were identified in the RlpA pulldown and from an additional LT pulldown; PA0788 from the SltB1 pulldown, PA2854 from the MltF2 pulldown, and PilA from the MltG pulldown. Binding partners highlighted in blue were identified from all eleven LT pulldowns. HP denotes hypothetical protein. View Supplementary Tables 2 and 3 for all 71 putative binding partners and for their enrichment data and attributed names, functions, and localizations.

**Fig. 4:**
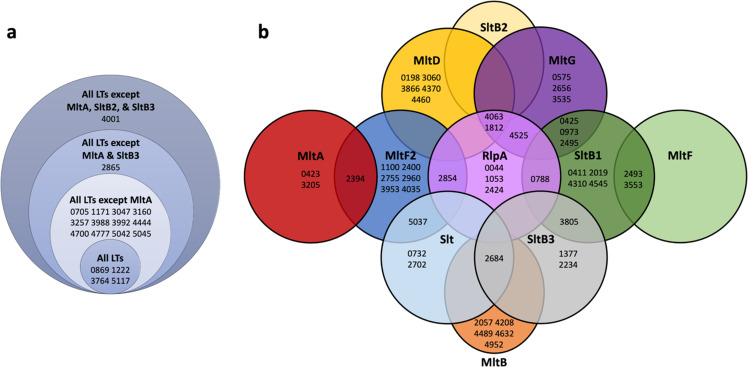
Venn Diagram representations of the 71 identified putative binding partners through all LT pulldowns. **a** Stacked diagram that only displays partners across a minimum of eight different LTs. **b** Grouping of the remaining putative partners across the eleven LTs. Six partners are not represented in this figure due to placement constraints: PA0041 and PA1091 (both with SltB2 and SltB3), PA2530 (across MltB, MltD, MltF2, and SltB3), PA3020 (with MltG, MltF2, and Slt), PA3999 (across MltG, MltF, Slt, and SltB1), and PA5043 (with MltF and Slt). All partners are listed with their respective gene locus tag. View Supplementary Table 2 for their fold-enrichment data.

Samples were analyzed by UHPLC and MS-MS/MS analysis^62,63^. RAW and mgf converted files were searched using Paragon and MaxQuant and quantified using Label-Free Quantification (MaxLFQ). The Pseudomonas FASTA database was obtained from the Pseudomonas Genome Database (Cystic Fibrosis Foundation, Therapeutics)^64,65^. Protein quantification measured the fold-enrichment of LT·bait-identified proteins relative to the control without LT protein^66,67^. Proteins detected exclusively in bait samples at high confidence were assigned a maximum fold-change of 64 (= 2^6^) to reflect the certainty of detection, but limit the fold-enrichment to the dynamic range of MaxLFQ^68,69^. The resulting list of putative interacting partner proteins was prioritized. The criteria for this prioritization were (i) predicted localization into the periplasm and (ii) enrichment of >50-fold. A total of 71 putative binding partners were identified across all eleven LT pulldowns. View Supplementary Tables 2 and 3 for their enrichment data and attributed names, functions, and localization, respectively. Selected putative binding partners from the list of 71 are shown in Fig. 3. Out of the total of the 71 putative binding partners, PBP7, MltA, MltF, RlpA, and TypA were identified across all LT pulldown experiments (Fig. 3, highlighted in blue). Three more binding partners—PA0788, PA2854, and PilA—were identified from the RlpA pulldown and one other LT pulldown: those of SltB1, MltF2, and MltG, respectively (Fig. 3, highlighted in gray). ExoT, SlyB, and PvdL are the binding partners uniquely identified in the RlpA pulldown experiment (Fig. 3). Figure 4 displays Venn diagram representations of the 71 identified putative binding partners for all of the eleven LT pulldowns. Putative protein partners were arranged across two different Venn diagrams, to appreciate a category of proteins that was shared across a minimum of eight LTs and another category focused on putative partners that are unique to an individual LT (Fig. 4a, b, respectively). Eighteen putative partners are diagrammed in Fig. 4a, of which 12 are shared among all LTs except MltA, and four partners across all LTs. As for Fig. 4b, MltF2 is the LT with the most unique putative partners, while there are no unique putative partners for SltB2 and MltF. Overall, SltB1 has the most enriched putative partners and most shared across other LTs, 31 and 27 partners, respectively.

**Fig. 5:**
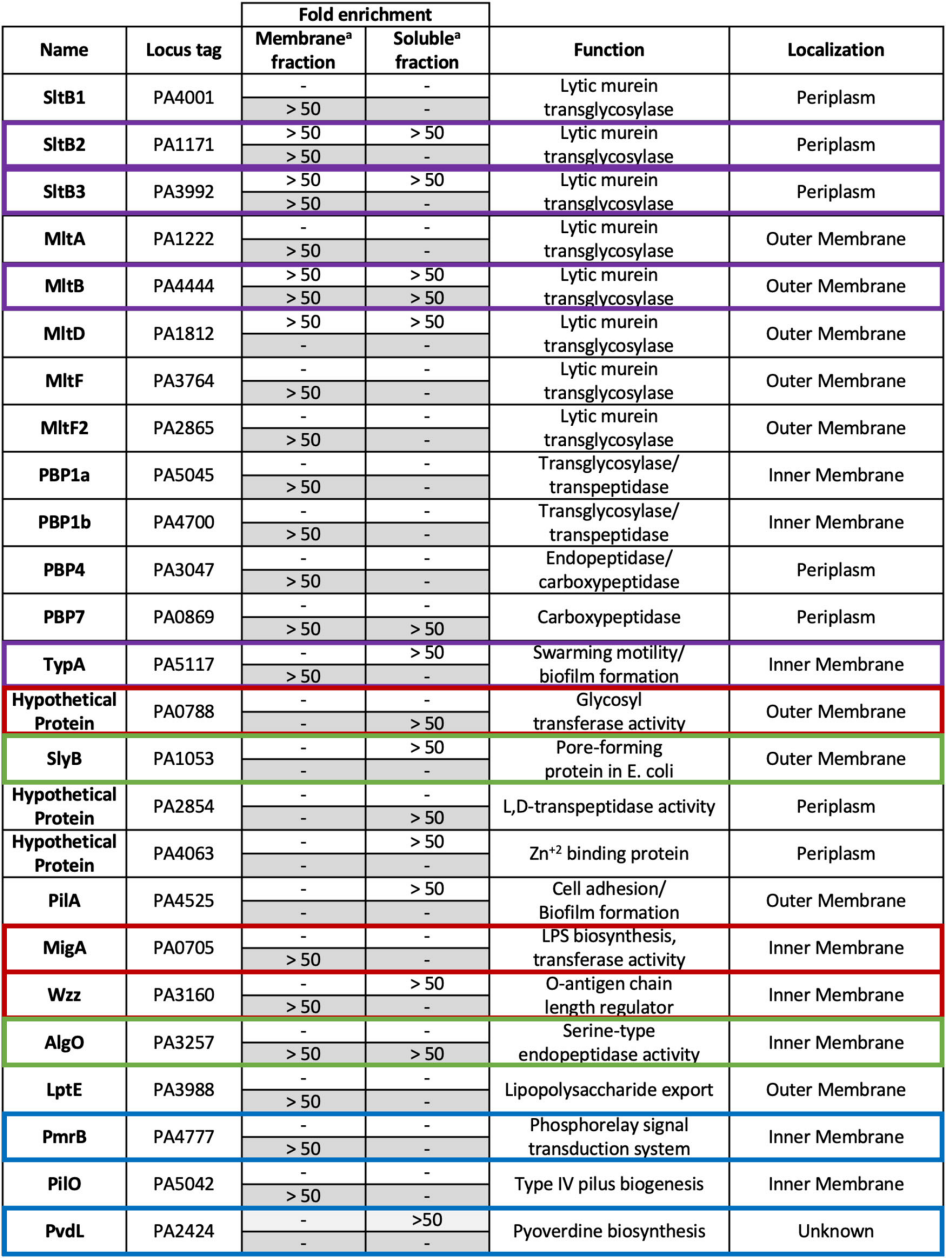
RlpA-dependent pulldown enrichment of putative partner proteins. ^**a**^Fold-enrichment of putative protein partners are calculated as the ratio of spectral count intensity of each run, compared to control (without RlpA-Δ32). A dash indicates no enrichment. Unhighlighted cells (white background) signify the experiment with a crosslinker, and highlighted cells (gray background) indicate the result without a crosslinker. Entries boxed in red indicate proteins that aggregated on the expression of the genes, those in blue were not studied, and those in green were non-direct interactors by MST (SlyB showed interactions with RlpA-Δ32 by SPR, which could not be quantified). All localization entries, regardless of classification, reside in the periplasmic milieu. Periplasm denotes protein that resides in the milieu and is soluble. The outer membrane denotes protein that is bound to the inner leaflet of the outer membrane. The inner membrane denotes protein that is bound to the outer leaflet of the inner membrane. Localizations and functions were collected from the Pseudomonas Genome Database (Cystic Fibrosis Foundation, Therapeutics). The oligomeric states of three proteins of Fig. 5 are known: SltB1 homodimer (*P. aeruginosa*), MltA homodimer (*Acinetobacter baumannii*), PBP1a homodimer (*Streptococcus pneumoniae*), PBP1b homodimer (*E. coli*), PilO homodimer (*P. aeruginosa*). The molecular weights of these proteins are listed in Supplementary Table 1.

A total of 25 proteins were identified from the RlpA pulldown experiment (Fig. 5). These proteins were grouped into seven clusters, depending on their functions (or presumed functions). These clusters were (1) other LTs; (2) penicillin-binding-proteins (PBPs); (3) lipopolysaccharide (LPS)-interacting proteins; (4) proteins involved in the biosynthesis of the pili (fimbriae); (5) proteins involved in the biosynthesis of biofilm; (6) proteins of the type-VI secretion system (T6SS); and (7) proteins of resistance-nodulation cell division multidrug-efflux. The genes for the 25 proteins were cloned (Supplementary Table 1). Twenty of these genes expressed well, whose corresponding proteins were purified to homogeneity. While cloning of the genes for PA0788, *wzz*, and *migA* (Fig. 5, boxed in red) was successful, their proteins were insoluble, despite our efforts. PmrB and PvdL (Fig. 5, boxed in blue) are large polytopic transmembrane proteins spanning the inner membrane. Purification of these proteins was not attempted.

### The RlpA constructs

We decided to study the partners of RlpA in greater detail. The sequence of the *P. aeruginosa* PAO1 *rlpA* gene (PA4000) corresponds to a protein of 342 amino acids. This protein shows an *N*-terminal signal peptide (amino acid residues 1–26); a cysteine-containing lipobox, which identifies the protein for translocation across the periplasm for covalent transfer to the lipid of the inner leaflet of the outer membrane (amino acids 24–29, LSSCSS); a linker sequence (amino acid residues 27–81), a lytic transglycosylase domain (amino acids 101–189, Pfam: PF03330), and an expansin-type domain (amino acids 196–246), which connects to the peptidoglycan-binding SPOR domain (amino acids 264–342, Pfam: PF05036). How the expansin domain is structurally ordered by the adjacent LT and SPOR domains is not evident from sequence analysis. We note in passing here that the expansin LPXXG sequence motif positioned adjacent to the catalytic aspartate of the LT domain of RlpA, is also found in the *P. aeruginosa* SPOR domain-containing proteins PA5037 (homolog of *E. coli* DamX) and PA5052 (homolog of *E. coli* FtsN). Three soluble RlpA constructs were made for the identification of its interacting partners. Figure 1b gives schematic representations of the RlpA constructs and lists the primary structure of RlpA. Supplementary Fig. 1 displays the primary structures of the RlpA constructs.

A truncated *rlpA* gene of *P. aeruginosa* PAO1 (amino acid residues 32–342; lacking the *N*-terminal signal peptide and the lipobox sequence) was cloned into pET28a(+) vector (Supplementary Table 1). Gene expression was induced with isopropyl-β-d-1-thiogalactopyranoside (IPTG). Soluble recombinant RlpA-Δ32 protein was purified to homogeneity by Ni-NTA affinity chromatography, yielding approximately 30 mg of pure *N-*terminally His-tagged RlpA-Δ32 from a 1 L culture. The two other RlpA constructs (RlpA-Δ81 and RlpA-SPOR) were made using procedures similar to those described above (Fig. 1b). RlpA-Δ81 (amino acid residues 82–342; lacking the *N*-terminal signal peptide and the linker sequence) and RlpA-SPOR (amino acid residues 264–342; lacking the *N-*terminal signal peptide, linker sequence, lytic transglycosylase domain, and the expansin-type domain) were cloned into pET28aTEV vector (Supplementary Table 1). Gene expression was induced with IPTG. Soluble recombinant RlpA-Δ81 and RlpA-SPOR were individually purified to homogeneity by Ni-NTA affinity chromatography, yielding approximately 25 and 12 mg of *N*-terminally His-tagged RlpA-Δ81 and RlpA-SPOR from a 1 L culture, respectively.

### MST and SPR evidence for the formation of RlpA complexes

**Fig. 6:**
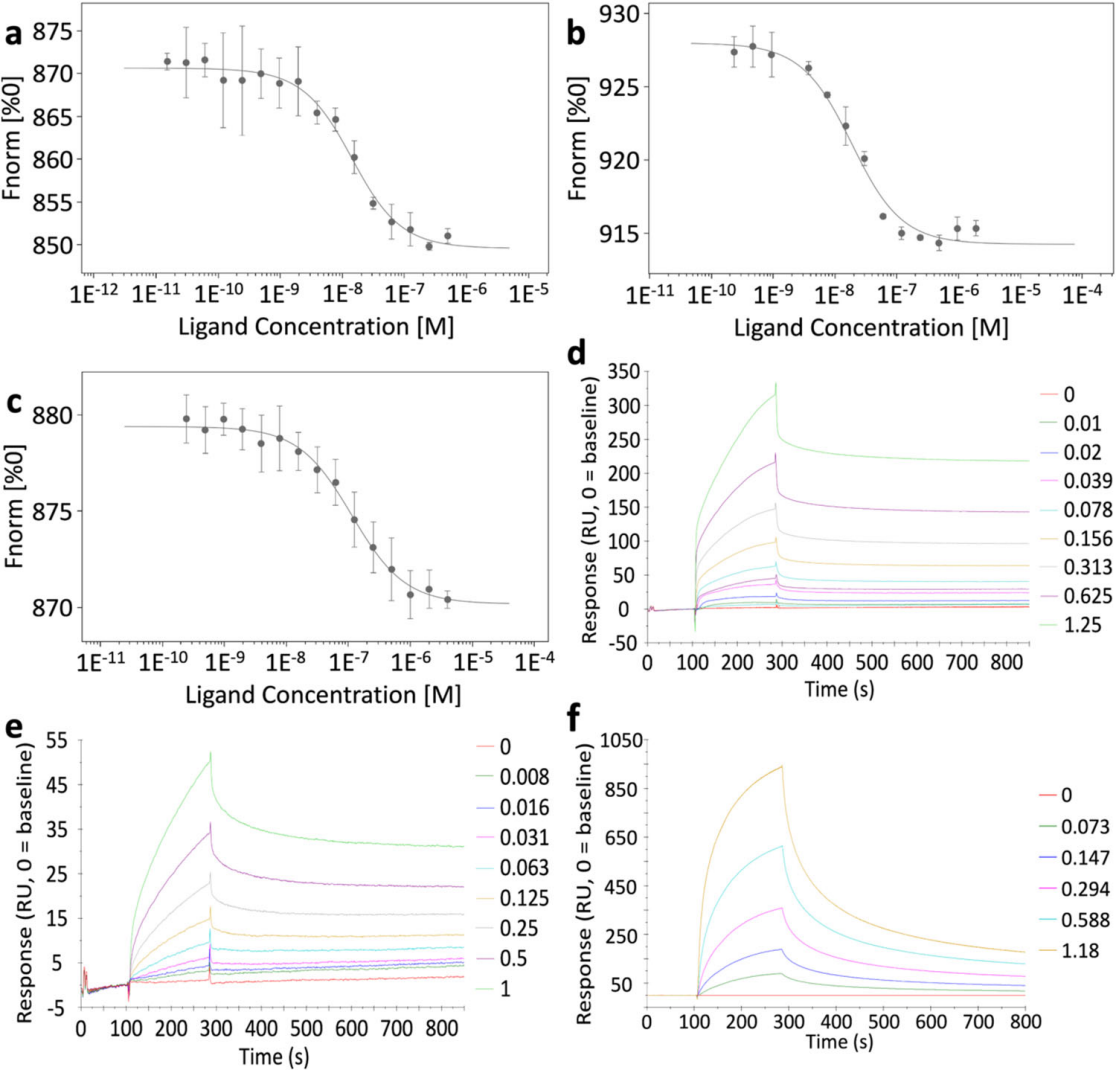
The MST dose-response curves and SPR sensorgrams. The dose-response curve between **a** RlpA-Δ32 and MltF2, **b** RlpA-Δ32 and PBP1b, **c** RlpA-Δ32·MltF2 and PBP1b. RlpA-Δ32 at a concentration of 5 nM is set as the fluorescently-labeled target throughout each MST run (ligand units are in molar). **d** RlpA-Δ32 and MltF2 SPR sensorgram, with concentrations of the latter decreasing in two-fold dilution. **e** RlpA-Δ32 and PBP1a SPR sensorgram, with concentrations of the latter decreasing at five-fold dilutions. **f** RlpA-Δ32·MltF2 and PBP1a SPR sensorgram, with concentrations of the latter decreasing at two-fold dilutions. RlpA-Δ32 is set as the immobilized ligand on the surface of the carboxymethylated dextran chip throughout the SPR runs. The response is measured in response units (RU). Concentration units for SPR experiments are given in μM. The baseline is corrected to zero. **a**–**c** Data were presented as means ± SD from triplicate experiments. View Supplementary Data for source data.

Assessment of possible protein–protein interaction with the soluble RlpA constructs was made using microscale thermophoresis (MST) and surface-plasmon resonance (SPR) experiments. MST validates protein–protein binding in solution as a consequence of the difference in the thermophoretic movement of a fluorescently tagged biomolecule within a complex. MST requires the fluorescent labeling of one of the proteins, for which RlpA-Δ32 was selected. Fluorescently labeled RlpA-Δ32 was mixed with solutions of the partner protein candidates (16 concentrations at two-fold serial dilution) to assess complex formation. Curve fitting of normalized fluorescence against concentration for those proteins that exhibited saturable complexation gave the *K*_D_ values presented in Table 1. Figure 6 shows exemplary data. The MST dose-response curve for the binary interactions between RlpA-Δ32 and MltF2 (Fig. 6a), and for RlpA-Δ32 and PBP1b (Fig. 6b), were both fit to the one-to-one binding. The *K*_D_ for RlpA-Δ32·MltF2 was 12 ± 2 nM and that for RlpA-Δ32·PBP1b was 16 ± 3 nM (Table 1, left column). Eighteen of the 20 candidates exhibited binding interactions by MST (Table 1, left column). The *K*_D_ values of Table 1 are listed from the strongest to the weakest interaction. Supplementary Fig. 3 exhibits the MST traces and dose-response curves of binary RlpA-Δ32 combinations tested. Two proteins—SlyB and AlgO (Fig. 5, boxed in green)—did not show binding to RlpA-Δ32 by MST. This outcome might indicate that SlyB and AlgO interact with another binding partner that has direct contact with RlpA-Δ32. Thus, highly-enriched indirect interactors like SlyB and AlgO, were potentially co-enriched along with direct partner proteins of RlpA-Δ32. This outcome, if valid, would indicate higher-order complexation of RlpA with binding partners. Alternatively, one can interpret the results as false positives for the proteomics analysis.

**Table 1:** The *K*_D_ values for RlpA-Δ32 interactions as measured by MST.

	RlpA (nM)^a^	RlpA + MltF2 (nM)^b^
MltF2	12 ± 2	N/A^a^
PBP1b	16 ± 3	57 ± 15
PA2854	58 ± 11	55 ± 9
MltA	67 ± 13	35 ± 5
LptE	77 ± 19	150 ± 32
SltB3	92 ± 9	390 ± 110
PBP1a	92 ± 9	45 ± 12
MltB	96 ± 29	110 ± 13
MltF	101 ± 16	260 ± 40
SltB2	110 ± 15	150 ± 29
SltB1	140 ± 19	95 ± 12
PA4063	201 ± 26	105 ± 13
MltD	240 ± 54	220 ± 26
PilA	240 ± 43	309 ± 85
PilO	350 ± 73	208 ± 45
PBP7	430 ± 90	NBD
PBP4	550 ± 110	240 ± 32
TypA	1000 ± 190	1800 ± 300
AlgO	NBD^c^	16000 ± 4500
SlyB	NBD^c^	NBD^c^

^a^Values denote the dissociation constants for the binary interactions.

^b^Competition for binding to RlpA by two partner proteins.

^c^NBD for “no binding detected”. RlpA-Δ32 construct was used for this experiment. Data were presented as means ± SD from triplicate experiments.

To investigate potential ternary combinations involving RlpA-Δ32 and two other partner proteins, we set up additional MST experiments in which a binary complex was initially formed between RlpA-Δ32 and MltF2, the strongest interacting complex (Table 1, left column). As described above, solutions of RlpA-Δ32 and MltF2 (fixed final concentration of 5 and 55 nM, respectively, >4-fold above *K*_D_) were mixed with a solution of the partner protein candidates (16 concentrations at two-fold serial dilution). Figure 6c shows the MST dose-response curve for the ternary interactions between RlpA-Δ32·MltF2 and PBP1b. The result fits one-to-one-to-one binding. The *K*_D_ for RlpA-Δ32·MltF2·PBP1b was 57 ± 15 nM (Table 1, right column and Fig. 6c). Table 1 (right column) displays all of the *K*_D_ ternary RlpA-Δ32·MltF2 combinations measured in these analyses. Interestingly, there was no binding detected by PBP7 to the RlpA·MltF2 complex (Table 1, right column). Moreover, taking RlpA-Δ32·PBP7 binding into consideration (Table 1, left column), these results would suggest that the PBP7 and MltF2 binding sites on RlpA-Δ32 overlap. In addition, no binding was observed for the RlpA-Δ32·MltF2·SlyB interaction (Table 1, right column). The *K*_D_ for RlpA-Δ32·MltF2·AlgO was 16 ± 4.5 μM (Table 1, right column and Supplementary Fig. 4s). This result supports the notion that non-direct partners were co-enriched along with a direct partner protein of RlpA-Δ32. Therefore, the experiment supports the presence of higher-order complexation of RlpA with binding partners. Supplementary Fig. 4 displays MST traces and dose-response curves of ternary RlpA-Δ32 combinations tested. Supplementary Fig. 5 provides negative controls for binding to RlpA-Δ32. Soluble derivatives of the binding-partner proteins were used for all MST analyses.

SPR evaluates the rate constants for association (*k*_on_) and for dissociation (*k*_off_) in complexes, and thus complements the MST analysis. Our initial SPR analysis used RlpA-Δ32 covalently bound to the chip surface and evaluated the partner proteins as the flow analytes. Figure 6 displays the sensorgrams for the binding of RlpA-Δ32 and MltF2, RlpA-Δ32 and PBP1b, and RlpA-Δ32·MltF2 and PBP1a (Fig. 6d–f, respectively). The *k*_on_ and *k*_off_ values for each were fit to a one-to-one model. The second-order rate constants for association (*k*_on_) were 43,760 ± 1600 M^–1^ s^–1^ for RlpA-Δ32 and MltF2, and 9100 ± 160 M^–1^ s^–1^ for RlpA-Δ32 and PBP1b (Table 2). The respective *k*_off_ values were 2.8 ± 0.1 × 10^–4^ and 5.0 ± 0.1 × 10^–4^ s^–1^ (Table 2). The calculated *K*_D_ values from the ratio *k*_off_/*k*_on_ for the RlpA-Δ32·MltF2 complex was 6 ± 2 nM, and for the RlpA-Δ32·PBP1b complex was 55 ± 6 nM (Fig. 6d, e, respectively). Table 2, gives the *k*_on_, *k*_off_, and *K*_D_ values for the SPR analyses. For most of the proteins, the *K*_D_ values measured by MST are in good agreement with those measured by SPR (within five-fold of each other). This is consistent with the respective limitations of each method of analysis (MST, use of a fluorescently-labeled protein; SPR, use of immobilized protein). Supplementary Fig. 6 gives the SPR sensorgrams of the binary RlpA-Δ32 interactions tested.

**Table 2:** The kinetics for RlpA-Δ32 binary interactions as measured by SPR.

	*K*_D_ (nM)	10^-3^ *k*_on_ (M^-1^ s^-1^)	10^3^ *k*_off_ (s^-1^)
MltF2	6 ± 2	44 ± 2	0.28 ± 0.01
PBP1b	55 ± 6	9.1 ± 0.2	0.50 ± 0.01
PA2854	65 ± 6	4.9 ± 0.2	0.32 ± 0.02
PBP4	130 ± 4	6.7 ± 0.1	0.86 ± 0.01
SltB3	140 ± 40	5.5 ± 0.1	0.79 ± 0.01
PBP1a	340 ± 40	4.2 ± 0.1	1.44 ± 0.01
PBP7^a^	*	5.9 ± 0.2	*
MltD	430 ± 190	2.7 ± 0.1	1.16 ± 0.30
MltB	620 ± 170	1.27 ± 0.03	0.79 ± 0.01
PA4063	810 ± 340	1.8 ± 0.1	1.45 ± 0.02
SltB2	1040 ± 100	0.48 ± 0.01	0.5 ± 0.01
PilA	1980 ± 100	1.10 ± 0.04	2.18 ± 0.01
TypA	2020 ± 300	0.38 ± 0.01	0.77 ± 0.20
PilO	3130 ± 200	0.88 ± 0.04	2.75 ± 0.02
SltB1	3850 ± 1700	0.33 ± 0.01	1.27 ± 0.01
MltF^a^	*	0.50 ± 0.02	*
MltA	N/A	BD^b^	BD^b^
LptE	N/A	BD^b^	BD^b^
SlyB	N/A	BD^b^	BD^b^
AlgO	NBD^c^	NBD^c^	NBD^c^

	Higher-affinity *K*_D_ (μM)	Higher-affinity *k*_off_ (M^-1^ s^-1^)	Lower-affinity *K*_D_ (μM)	Lower-affinity *k*_off_ (M^-1^ s^-1^)
PBP7	10 ± 2	0.06 ± 0.01	110 ± 24	0.64 ± 0.10
MltF	2 ± 0.3	0.001 ± 0.001	100 ± 15	0.05 ± 0.02

Kinetic parameters (*K*_D_, *k*_*on*_, *k*_off_) for RlpA-Δ32 combinations tested. Two phases of dissociation for RlpA-Δ32 with PBP7 and with MltF.

^a^Binding interaction displays biphasic behavior, with the placeholder indicated by an asterisk.

^b^BD for “binding detected”, but the data were outside the ability to extract reliable kinetic constants.

^c^NBD for “no binding detected”. Data were presented as means ± SEM from triplicate experiments.

SPR analysis of the RlpA-Δ32 interaction with MltF and with PBP7 gave a single discernable phase for association and two phases for dissociation—a “higher-affinity” *k*_off_ and a “lower-affinity” *k*_off_. The two dissociation phases are consistent with a conformational change after the two proteins form a complex with each other (Table 2). At least for the case of MltF, a dramatic conformational change by X-ray crystallography has been documented^70^. Two *K*_D_ values for each binding partner can be evaluated from the ratios of *k*_off_/*k*_on_, as two distinct *k*_off_ events were seen for the two phases. PBP7 displayed a “higher-affinity” *K*_D_ of 10 ± 2 μM and a “lower-affinity” *K*_D_ of 110 ± 24 μM (Table 2). Similarly, the results of MltF show a “higher-affinity” *K*_D_ of 2 ± 0.3 μM and a “lower-affinity” *K*_D_ of 100 ± 15 μM (Table 2). The complexes of RlpA-Δ32 with PBP7 and with MltF are not particularly strong. The range of values in Table 2 for *k*_on_ was >130-fold, and for *k*_off_ was >4600-fold. Binding interactions between the surface-immobilized RlpA-Δ32 and MltA, LptE, and SlyB were noted by the sensorgrams, however, the quality of the data did not allow extraction of the kinetic parameters. SlyB is notable since MST did not document interactions with RlpA-Δ32 (Table 1). A conformational change may be a prerequisite for the interaction, as suggested by the time-resolved SPR measurements. However, the complexity of the conformational states did not lend themselves to suitable analysis for rate measurements. Hence, AlgO is the only protein identified by the pulldown strategy that did not show (by either method) a direct interaction with RlpA-Δ32, however, displayed itself as an indirect interactor for higher-order complexation with the protein.

Ternary combinations involving RlpA-Δ32, MltF2, and a third partner were also evaluated by SPR (Fig. 6f and Supplementary Fig. 7). However, given the experimental setup of SPR, we first linked RlpA-Δ32 to the chip, followed by cross-linking MltF2 to RlpA-Δ32. The covalent complex of RlpA-Δ32·MltF2 now behaves as a single fixed ligand on the surface of the chip. This modified chip was used for the purpose of testing the formation of ternary complexes with other protein candidates. PBP1a, PBP7, TypA, SltB1, and MltF (Supplementary Fig. 7) were examined for this purpose. The *k*_on_ was 51,700 ± 200 M^–1^ s^–1^ and the *k*_off_ was 4.8 ± 0.8 × 10^−3^ s^−1^ for the RlpA-Δ32·MltF2·PBP1a complex (Supplementary Fig. 7). The calculated *K*_D_ values from the ratio *k*_off_/*k*_on_ for the RlpA-Δ32·MltF2·PBP1a complex was 280 ± 17 nM (Fig. 6f). SPR evaluation detected interactions between PBP1a, TypA, SltB1, and MltF with the cross-linked RlpA-Δ32·MltF2 complex, but not with PBP7 (Supplementary Fig. 7). These results agree with the MST ternary combination experiments. These data reveal that the binding sites of PBP7 and MltF2 on the RlpA-Δ32 surface overlap, in contrast to other identified ternary combinations.

Additional SPR experiments used truncated RlpA constructs. The linker sequence (amino acid residues 27–81) and each of the three sub-domains (LT, expansin, SPOR) may function in partner-protein recognition (Fig. 1b). Two truncated RlpA proteins—RlpA-Δ81 and RlpA-SPOR—were explored to differentiate among these possibilities (Supplementary Fig. 8,9, respectively). Attempts to produce three additional RlpA constructs (the LT domain alone, the LT domain plus the expansin-type domain, and the SPOR domain with the expansin-type domain) gave inclusion bodies from which soluble proteins could not be isolated. SPR analyses of the RlpA-Δ81 and RlpA-SPOR constructs were done with eight partner proteins chosen from several of the clusters (Table 3 and Supplementary Figs. 8,9, respectively). Asserting 10-fold as the threshold for a meaningful difference in kinetic parameters, diminished *K*_D_ values for RlpA-Δ81 with partner proteins PA2854 and SltB3 were noted. The binding of RlpA-SPOR to MltF2, PA2854, SltB3, and PA4063 was reduced significantly. Interaction with TypA strengthened, principally due to an enhanced value for *k*_on_.

**Table 3:** The kinetics for binary interactions with the RlpA-Δ81 and RlpA-SPOR constructs, as measured by SPR.

	RlpA-Δ81			RlpA-SPOR		
	*K*_D_ (nM)	10^-3^ *k*_on_ (M^-1^ s^-1^)	10^3^ *k*_off_ (s^-1^)	*K*_D_ (nM)	10^-3^ *k*_on_ (M^-1^ s^-1^)	10^3^ *k*_off_ (s^-1^)
MltF2	3 ± 2	14.9 ± 0.3	0.04 ± 0.01	98 ± 21	7.9 ± 0.2	0.77 ± 0.01
PA2854	2270 ± 230	0.33 ± 0.01	0.75 ± 0.03	1410 ± 410	0.27 ± 0.01	0.38 ± 0.01
SltB3	BD^a^	BD^a^	BD^a^	2140 ± 800	0.37 ± 0.01	0.79 ± 0.01
PBP1a	94 ± 24	18.8 ± 0.4	1.77 ± 0.02	190 ± 34^c^	2.6 ± 0.4^c^	0.49 ± 0.05^c^
MltD	56 ± 1	69 ± 5	3.9 ± 0.3	200 ± 110	13.8 ± 0.7	2.7 ± 0.1
PA4063	1080 ± 340	1.8 ± 0.1	1.94 ± 0.01	NBD^b^	NBD^b^	NBD^b^
TypA	74 ± 13	6.4 ± 0.1	0.47 ± 0.01	4250 ± 100	0.08 ± 0.01	0.34 ± 0.01
PilO	1010 ± 260	0.75 ± 0.05	0.76 ± 0.02	5760 ± 490	0.76 ± 0.01	4.38 ± 0.04

^a^BD for “binding detected”, but the data kinetic constants could not be extracted.

^b^NBD for “no binding detected”.

^c^An alternate fitting model was applied to these sensorgrams in response to stoichiometry data collected from ITC experiments (Fig. 7). Data were presented as means ± SEM from triplicate experiments.

### A complementary pulldown experiment

A solution of recombinant *N*-terminal His-tagged RlpA-Δ32 was incubated separately with the two proteome preparations, in the presence of the protein crosslinker bis(sulfosuccinimidyl)suberate (BS^3^). Ni-NTA resin was added to this mixture to entrap RlpA-Δ32 and partner (“prey”) protein(s) in the preformed covalent complexes (Fig. 2b). The two control experiments were the absence of the proteome lysate (wild-type PAO1 strain) and the absence of RlpA-Δ32. Bound proteins were identified by mass spectrometry. As RlpA is a membrane-bound protein, and so are 17 of its identified partners, there exists a favorable entropic factor for the complexation of partner proteins in intact bacteria, which is absent in the recognition between the bait and prey proteins in solution. We would have been pleased to capture a single cross-linked example, validating the experiments of Fig. 2a. Indeed, four of the partners identified in the experiments of Fig. 2a—SltB2, SltB3, MltB, and TypA—were validated by this approach (Fig. 5, boxed purple).

### Stoichiometry for RlpA with binding partners through ITC

**Fig. 7:**
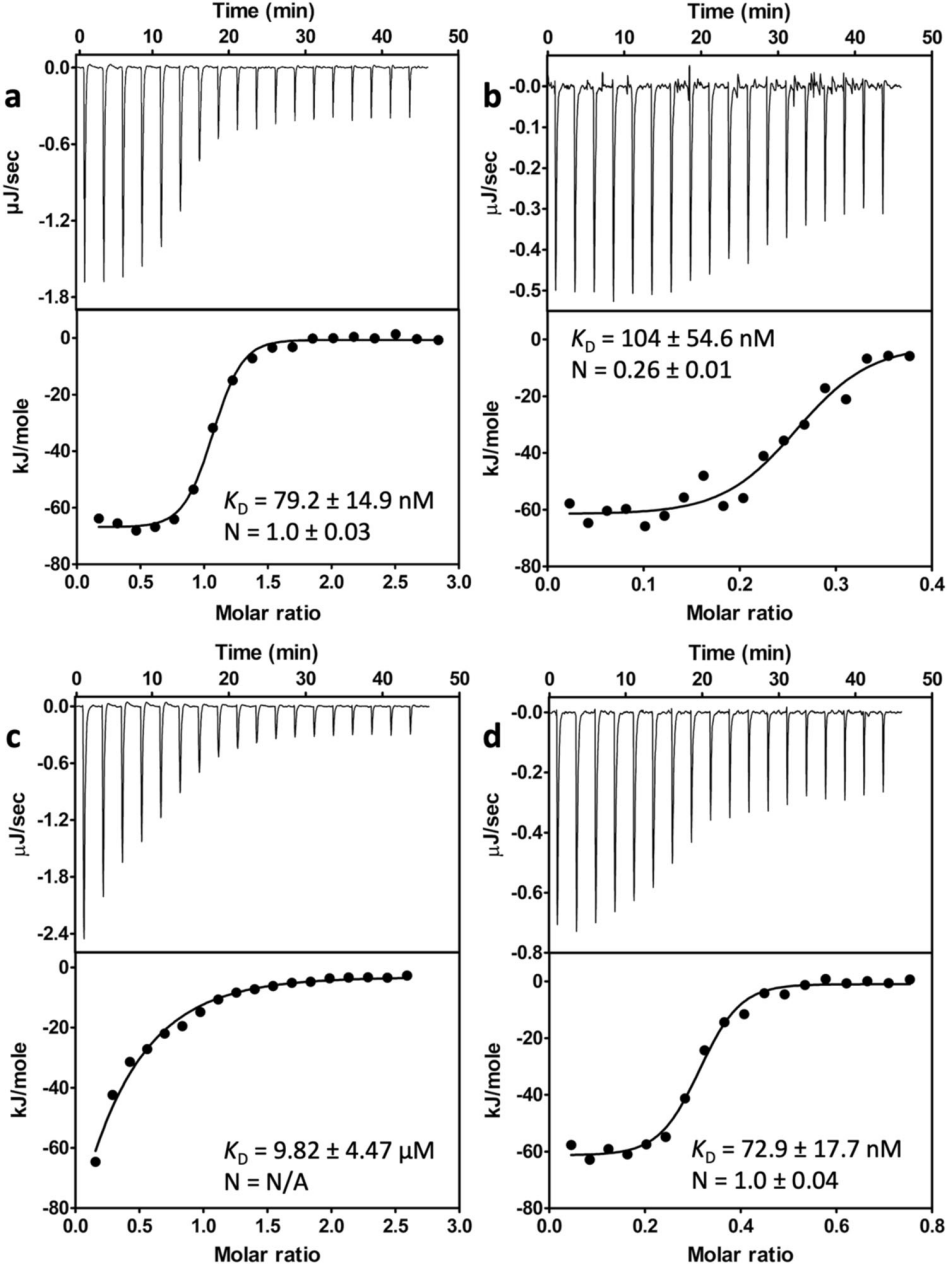
Representative ITC fitting results of RlpA-SPOR with binding partners. The ITC fitting results of RlpA-SPOR with **a** SltB3, **b** PBP1a, **c** SltB1, and **d** PilA. The thermodynamic data were collected from the titration of RlpA-SPOR into the cell with a respective binding partner for **b**–**d**. Data were collected from the titration of SltB3 into RlpA-SPOR. All parameters were calculated by fitting into a one-set-of-sites model. Buffer utilized for ITC was 50 mM Tris and 300 mM NaCl, pH 8.0. Data were presented as means ± SD from triplicate experiments. RlpA-SPOR construct was used for this these experiments. View Supplementary Data for source data.

Isothermal-titration calorimetry (ITC) experiments were utilized to garner further evidence for protein–protein interactions and the stoichiometry data between RlpA and binding partners in solution. RlpA-Δ32 construct was initially used, however, aggregation was present upon RlpA-Δ32 titration into the sample cell containing the binding partner under the conditions of the ITC experiments. Assuming that the flexible segments of RlpA-Δ32, linker sequence and/or expansin-type domain, might have been at the root of aggregation under the ITC conditions, the RlpA-SPOR construct was used with SltB3, PBP1a, SltB1, and PilA as partners. Their respective thermographs, binding kinetics, binding stoichiometry, and fitting can be seen in Fig. 7. The dissociation constant for RlpA-SPOR and SltB3 was evaluated at 79 ± 15 nM with a stoichiometry of 1.0 ± 0.03 (Fig. 7a). The corresponding dissociation constant by SPR elicits an ~27-fold higher value (Table 3). Such a difference is inherent in the two methodologies. SPR requires covalent attachment of a biomolecule on the surface of the chip, which implies a specific orientation of the ligand-mediated through immobilization. While ITC has titration of one biomolecule onto another within an actively stirring sample cell. Figure 6b–d display thermographs and fitting for RlpA-SPOR with PBP1a, SltB1, and PilA, respectively. Dissociation constants and stoichiometry are measured at 104 ± 55 nM and 0.26 ± 0.01 for RlpA-SPOR with PBP1a, respectively (Fig. 6b). The kinetics measured through SPR for the RlpA-SPOR and PBP1a interaction are in good agreement with one another (Table 3). For RlpA-SPOR with SltB1, the dissociation constant was determined at 9.8 ± 4.5 μM (Fig. 7c). Stoichiometry could not be assessed for the RlpA-SPOR and SltB1 interaction since a sigmoidal curve was not achieved to quantify one. The dissociation constant and stoichiometry are measured at 73 ± 18 nM and 1.0 ± 0.04 for RlpA-SPOR with PilA, respectively (Fig. 7d). The kinetic values obtained by ITC further validate the MST and SPR results.

## Discussion

No aspect of the bacterial cell cycle is simple. Rod-shaped bacteria lengthen and then, at the mid-point of the lengthened cell, form a septum as a prelude to cell separation. The lengthening of the cell envelope is catalyzed by the multi-protein, multi-enzyme ensemble termed the elongasome. Septal growth is catalyzed by the equally complex divisome. Notwithstanding their different identities, the elongasome and the divisome spatially coincide at mid-cell for 40% of the *E. coli* cell cycle^19^. The divisome assembles at mid-cell by hierarchical recruitment of its proteins^71–76^. FtsN is essential to cell division. It is a bitopic protein having a short but functionally critical cytoplasmic *N*-terminus, which engages the FtsA protein of the Z-ring, a transmembrane α-helix, and a large periplasmic domain. The periplasmic domain consists of short membrane-proximal helices, a glutamine-rich sequence, and a *C*-terminal SPOR domain^54,77^. A second key sub-structure of FtsN is a short helical sequence in its periplasmic linker, which is used to engage the aPBP (PBP1b) of the divisome^55,77,78^. *E. coli* RlpA also is a SPOR domain-containing protein. RlpA is an accessory component of the *E. coli* divisome, as evidenced by its protein–protein interaction with FtsK^59^. FtsK is a large, bitopic protein that acts at the late stage of septum formation as both a DNA translocase and as a checkpoint for final septal closure^59,60,79,80^. However, the role of RlpA in the *E. coli* divisome is structural and is not catalytic, as a result of a point mutation at the position of the catalytic aspartate (which is present in *P. aeruginosa* RlpA), and as evidenced by the absence of a phenotype upon its genetic deletion^38,49^.

In contrast, RlpA of *P. aeruginosa* has the required catalytic aspartic acid (D168), has enzymatic activity, and has a phenotype. Mutational inactivation of *P. aeruginosa* RlpA results, during exponential growth in low-osmotic media, in chains of shortened and rounded cells^38^. A similar RlpA phenotype in *V. cholerae* is interpreted to indicate a role for RlpA catalysis in daughter-cell separation^34,40,81^. Studies using RlpA-fluorescent protein fusions in both *E. coli* and *P. aeruginosa* localize a minor population of RlpA to sidewall foci, with the major population of RlpA at the Z-ring demarcation of the nascent septum^38,49,50^. A starting point for understanding RlpA function is the similarity of the operons of *E. coli* and of *P. aeruginosa*, which contain the *rlpA* gene (Supplementary Fig. 10). In both bacteria, the *rlpA* gene is in an operon of the elongasome. In *E. coli*, the *rlpA* gene is flanked by the genes *pbpA* (encoding PBP1a, the aPBP of the elongasome), *rodA* (the peptidoglycan glycosyltransferase of the elongasome and the functional partner of the bPBP transpeptidase, PBP2), and *dacA* (a cPBP of peptidoglycan stem processing, PBP5) (Supplementary Fig. 10)^82^. In *P. aeruginosa,* the flanking genes are *pbpA*, *rodA*, *sltb1* (SltB1 is a soluble lytic transglycosylase of the periplasm), and *dacC* (a cPBP of peptidoglycan stem processing, PBP5) (Supplementary Fig. 10)^38^. We identified (among others) PBP1a, PBP1b, SltB1, and the cPBP, PBP7 as RlpA interaction partners. Indeed, the strongest-binding proteins for RlpA (Tables 1,2) are two aPBPs (representing the elongasome and the divisome), seven other LTs, and two cPBPs. These data are consistent with RlpA incorporation into the elongasome, which is then brought into contact with, for its transfer to the divisome.

The core proteins of the *E. coli* divisome are FtsA (interacts with the cytoskeletal Z-ring ensemble), FtsE·FtsX (FtsEX, an early protein pair to the divisome which binds to FtsA, FtsK, FtsQ·FtsL·FtsB (FtsQLB), and FtsW·FtsI (FtsWI; FtsI is PBP3), and PBP1b·LpoB (LpoB is the lipoprotein regulator of PBP1b function)^8,10,12,74^. Although the divisome of *P. aeruginosa* is less studied as compared to the divisome of *E. coli*, the organization and function of the core proteins of the two divisomes appear similar^21^. In the core of *E. coli* divisome, PBP1b partners with at least three other proteins: FtsQLB^83^, PBP3^84^, and FtsN^77,85^. In turn, FtsN interacts with FtsA, FtsWI, and PBP1b^83,85–87^. The structural organization of the divisome is not known^73^. The complexity of these interaction networks indicates that the divisome is, as widely surmised, a complex three-dimensional multi-protein entity. As stated previously, amidase-catalyzed denuding of the mid-cell peptidoglycan at the Z-ring^39,51,53^ recruits the SPOR domain-containing protein FtsN, and presumably concurrently the other SPOR domain-containing proteins RlpA, DamX (PA5037), DedD (PA4278), FtsN (PA5052), and a fifth SPOR protein (PA3110) to initiate divisome activity^39,57,88,89^. DamX and DedD are regulatory proteins of aPBP catalysis^50,57,58,90^. A similar function in *P. aeruginosa* is presumed. Supplementary Fig. 11 gives the sequences of the *P. aeruginosa* SPOR proteins.

Our analyses implicate RlpA partnership with other LTs and cPBPs. Tables 1 and 2 have eight (with RlpA, nine) of the eleven LTs of *P. aeruginosa*. The LT family has extensive functional redundancy^33,35,38,40,91,92^. While data suggest strongly that the catalytic function of at least one LT is essential to these Gram-negative bacteria^92^, many of the individual LTs can assume these essential functions based on their reaction profiles with the cell-wall peptidoglycan^33^. Four LT functions have been identified. The first activity is the glycan sizing of nascent peptidoglycan catalyzed by MltG^29,30,32^. The second activity is the turnover and recycling of peptidoglycan strands liberated during cell-wall synthesis^93–95^. The third activity (as shown in *V. cholerae*, and possibly related to the second function) is the non-divisome clearance from the periplasm of uncrosslinked peptidoglycan strands liberated during cell-wall synthesis^81^. The fourth activity is cell shape-related facilitation of daughter-cell separation under hypo-osmotic conditions, giving the phenotype encountered upon loss of RlpA in both *P. aeruginosa* and *V. cholerae*^43,96^. We underscore that the redundancy of the catalytic reactions argues that the loss of activity of one LT can be compensated by those of others. Additional observations are pertinent. *E. coli* PBP1b binds to several LTs^43,96^. In *V. cholerae*, RlpA is assisted in daughter-cell separation by a second LT, MltC^34^. We interpret our data showing RlpA interaction with numerous other LTs (beyond the SltB1, expressed by its flanking gene) as reflecting both the accommodation of other LTs by RlpA, and, perhaps critically, their intrinsic functional redundancy. The SPOR localization of RlpA, as a unique attribute, might represent an advantage rather than a necessity. An accompanying question is the nature of this LT interaction. The oligomeric character of the protein components of the divisome is an important unsolved question^12^. *E. coli* PBP1b is functional as a dimer^97^. *E. coli* FtsBL is a tetramer^98,99^. The DNA translocase domains of FtsK organize as a hexamer^79,100,101^. Here, our soluble RlpA constructs behave as a monomer based on SEC and AUC analyses (Supplementary Fig. 12). Its interaction with the other LTs and PBPs, namely MltF2 and PBP1b as documented in our report, however, implicates stable hetero-oligomer (heterodimer or higher order) formation. As demonstrated through the RlpA-Δ32·MltF2·AlgO MST experiments (Table 1, right column).

A total of 71 putative binding partners were identified by the eleven LT pulldowns. As implicated by the binding data for RlpA, the overlap of binding partners across various LT pulldowns was expected. Five proteins were identified across all LT pulldowns: PBP7, MltA, MltF, RlpA, and TypA (Figs. 3, 4). Within the LT family, this result suggests that MltA, MltF, and RlpA participate in a larger range of protein–protein interactions than the other LTs. PBP7 interacts with numerous LTs. The promiscuity of this carboxypeptidase with respect to LT interaction suggests a close functional association between cPBPs and LTs in the cell wall processes. PA0788, PA2854, and PilA as binding partners (RlpA pulldown and one other LT pulldown, Figs. 3, 4) gives an additional layer of LT interaction. The involvement of RlpA within glycosyltransferase and transpeptidase activities is akin to its interaction with other PBPs. RlpA interaction with PilA provides suggested involvement within processes such as biofilm formation and cell adhesin.

**Fig. 8:**
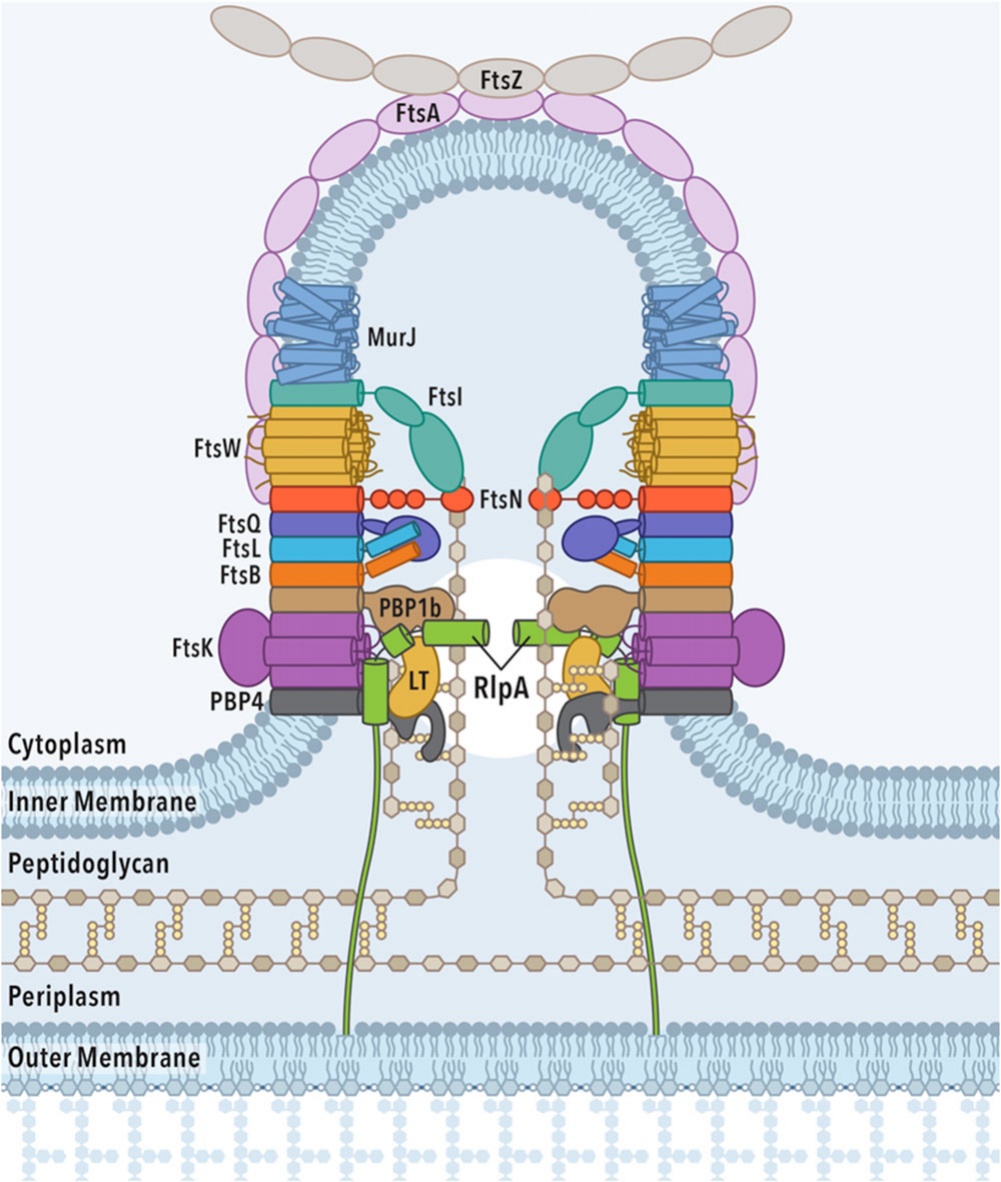
RlpA and its recruitment of binding-partners for the divisome during daughter-cell separation. This perspective illustrates a single snapshot of the entire daughter-cell-separation process. The placement of RlpA and select binding partners spatially demonstrate the interrelationship of such protein–protein interactions for the purposes of peptidoglycan denuding, orthogonal growth of the peptidoglycan, and as components for the multi-enzyme divisome complex. C2 symmetry within the septum is shown. “LT” placeholder can equate to LT binding partners; SltB1, SltB2, SltB3, MltA, MltB, MltD, MltF, or MltF2.

Within the limitations of the pulldown-enrichment mass-spectrometry experiment, our analyses identify the protein partners bound to RlpA. RlpA is recruited from the elongasome to the divisome, by its recognition of mid-cell denuded peptidoglycan, as divisome function initiates. The data of Tables 1, 2 incorporate, but do not differentiate, likely different interaction networks. Tables 1,2 should not be interpreted as a complete list of interactors with RlpA. Other interactors, such as FtsK in *E. coli* and MltC in *V. cholera*, in these species are bona fide interactors with RlpA^40,59^. FtsK is not identified as a RlpA-binding partner in *P. aeruginosa* by our pulldown-enrichment strategy. This difference in *P. aeruginosa* indicates either a different interactome for RlpA in *P. aeruginosa* compared to *E. coli*, or a limitation of our method of analysis. For example, FtsK could remain in the cell pellet during sample preparation for both soluble and membrane portions for the pulldown experiment, and thus not identified through MS/MS analysis. It is also conceivable that the interaction is not direct, but mediated through other proteins. Another possibility is that the level of expression of FtsK is not high enough to enable MS/MS identification. We reemphasize that low-copy number proteins will be missed through this methodology. The partnerships for RlpA, which are emphasized in the cartoon of Fig. 8 are an aPBP (such as PBP1b), a cPBP (such as PBP4), and a second LT (such as SltB1) in the divisome. STRING analysis of *E. coli* RlpA suggests a highly similar interaction network^59^.

Denuding the mid-cell peptidoglycan may have an additional purpose. Denuding is partial depolymerization. The peptidoglycan of the nascent septum must integrate with the peptidoglycan of the sidewall, but the progressive growth of the nascent septum is spatially orthogonal to the peptidoglycan of the sidewall. Denuding will allow reorientation of the sidewall peptidoglycan to support septal growth in the orthogonal direction. Moreover, mid-cell denuding may preserve a center-line position for RlpA in the new septum, for ultimate catalytic function in daughter-cell separation. As Fig. 8 implies, it is important to note that the two nascent growing peptidoglycan halves in the septum do not crosslink to one another. Otherwise, defective daughter-cell separation has been previously shown with loss of function RlpA in low-osmotic strength media^38^. A possible explanation is the involvement of LT and PBP4 functions that remove or prevent this crosslinking from occurring. Thus, LTs with PBP4 are catalytically active throughout the forward motion of the divisome. Figure 8 gives a cartoon perspective on the interrelationship among peptidoglycan denuding, orthogonal growth of the peptidoglycan, and a place for RlpA (and its protein network) as important (albeit peripheral) proteins of the divisome.

Notwithstanding that the formation of peptidoglycan multi-protein complexes is dynamic, driven possibly by multiple transient protein–protein interactions^102^, and not by protein partners binding to RlpA at the same time, this report opens the opportunity to explore the mechanistic implications of each of these partnerships. The opportunities include the interactions that we document for RlpA, as well as the ones in Supplementary Tables 2 and 3, where as many as 71 proteins have been identified as putative partners of lytic transglycosylases of *P. aeruginosa* strain PAO1. The incorporation of in vivo experiments to observe and gather spatiotemporal information are part of the future directions herein this study. RlpA serving roles as a general adapter protein would leave the LT as a structural and catalytic component of both divisome and elongasome complexes. It could be envisioned that at different cell cycle time points, RlpA would be in the divisome complex and cleave-denuded peptidoglycan strands in the septum or interact with components of the elongasome to meet the needs of the lateral sides of the pseudomonal cell.

## Methods

### Cloning

The RlpA gene (for constructs RlpA-Δ32, RlpA-Δ81, and RlpA-SPOR) were cloned from the *P. aeruginosa* PAO1 genomic DNA into pET28a(+) for RlpA-Δ32 using restriction enzymes KasI and XhoI (New England Biolabs), and into pET28aTEV using restriction enzymes KasI and XhoI (New England Biolabs) for RlpA-Δ81 and RlpA-SPOR. The binding-partner genes were cloned from the *P. aeruginosa* PAO1 genomic DNA into their corresponding vector: pET28a(+), pASK-IBA17k, using the corresponding restriction enzymes KasI, KpnI, and XhoI (New England Biolabs, Supplementary Table 1). The pASK-IBA17k plasmid vector derives from pASK-IBA17(+) (IBA Life Sciences), but is modified to encode a kanamycin-resistance cassette instead of the ampicillin-resistance cassette. Q5 Hot Start High Fidelity DNA polymerase (New England Biolabs) was used. The list of the primers used for cloning are given in Supplementary Table 1. The purity of the PCR reaction product(s) was determined using 1% agarose gel for 30 min at 100 V. The sequence of the gene in each case was confirmed by DNA sequencing on both strands (Molecular Cloning Laboratories).

### Gene expression

The plasmid, pET28a_*rlpA-Δ32*, was introduced into *E. coli* DH5α (Thermo Fisher Scientific) by heat-shock transformation, followed by a selection of transformants on an LB plate containing 30 μg mL^–1^ of kanamycin (Sigma-Aldrich). The gene in *rlpA-Δ32*-pET28a was confirmed by DNA sequencing (Molecular Cloning Laboratories). The plasmid was then introduced into *E. coli* BL21-star (DE3, Invitrogen) in a similar process for expression. The additional RlpA constructs and each recombinant plasmid for every binding-partner gene was introduced similarly into *E. coli* DH5α, confirmed by DNA sequencing (Molecular Cloning Laboratories), and then introduced into *E. coli* BL21-star (DE3). For the expression of recombinant plasmids for the binding partners that proved difficult to express, the plasmid was introduced into *E. coli* C43 (DE3, Invitrogen) in each case (Supplementary Table 1). We followed the expression procedure for pET28a-*dacB*, as described previously^33^.

### Protein purification

A single colony of *E. coli* BL21-star (DE3) transformant with pET28a-*rlpA-Δ32* plasmid was cultured overnight in LB media containing 30 μg mL^–1^ of kanamycin (Sigma-Aldrich). This culture was transferred into 1 L of fresh LB media and was allowed to grow at 37 °C until an OD_600_ of 0.6 was reached. Protein expression was induced with 0.5 mM isopropyl-β-d-1-thiogalactopyranoside (IPTG, IBI Scientific) at 16 °C overnight. The cell pellet was resuspended in 30 mL of lysis buffer [50 mM Tris pH 8.0 buffer, 300 mM NaCl, 20 mM imidazole, 0.05% Brij-35, 10 μg/mL of DNase I (bovine pancreas, Sigma-Aldrich), 10 μg mL^–1^ of lysozyme (chicken egg white, Sigma-Aldrich)]. Proteins were released from the cells by sonification on ice (1 min of sonication, 2 min rest on ice; 10 times). After centrifugation (45 min at 18,000 × *g*), the supernatant was loaded onto 5 mL of Ni-NTA resin (Macherey-Nagel). The resin was washed with 50 mL of lysis buffer. Proteins were eluted with a gradient of 20 to 500 mM imidazole (Sigma-Aldrich) in a total of 200 mL of elution buffer (50 mM Tris·HCl, 300 mM NaCl, pH 8.0). Fractions with recombinant protein (as verified by SDS-PAGE gel) were collected and concentrated (Amicon Ultra-Centrifugal Filter, 10-kDa cut-off). A typical yield of RlpA-Δ32 was 30 mg of protein from a 1 L culture. Approximately 10 mg of RlpA-Δ32 retained their recombinant poly-His-tag, while the rest were subjected to Thrombin cleavage. The protein solution was subjected to a Thrombin CleanCleave kit (bovine, Sigma-Aldrich). Cleavage of RlpA-Δ32 and subsequent removal of RlpA-Δ32 from thrombin-agarose was done according to the manufacturer’s instructions. Aliquots of both poly-His-tagged and cleaved RlpA-Δ32 (~4 mg mL^–1^) in 10 mM HEPES, 150 mM NaCl, 3 mM EDTA, 0.005% surfactant P20, pH 7.4 buffer were flash-frozen for storage at –80 °C. In this buffer, the RlpA-Δ32 protein was stable to thawing and was stable for days at 4 °C. Its solutions were re-frozen and re-thawed successfully. The molar concentration of the RlpA-Δ32 protein in the solutions was determined from the A_280 nm_ absorbance (using the calculated ε_280 nm_ = 47,000 L mol^–1^ cm^–1^), and cross-calibrated to the visible absorbance determined by Bradford protein assay (Thermo Fisher Scientific).

The two remaining RlpA constructs (RlpA-SPOR and RlpA-Δ81) were subjected to similar procedures, as described above. RlpA-Δ81 (amino acid residues 82–342; lacking the *N*-terminal signal peptide and linker sequence) and RlpA-SPOR (amino acid residues 264–342; lacking the *N-*terminal signal peptide, linker sequence, lytic transglycosylase domain, and expansin-type domain) were cloned into pET28aTEV vector (Supplementary Table 1). The plasmids, pET28aTEV-*rlpA-Δ81* and pET28aTEV-*rlpA-SPOR*, went through a similar transformation, expression, and purification methods as described above. The yield from a 1 L culture of soluble *N*-terminally His-tagged RlpA-Δ81 was approximately 25 mg. The yield of RlpA-SPOR was approximately 12 mg. Approximately 8 mg of RlpA-Δ81 and 4 mg of RlpA-SPOR retained their recombinant poly-His-tags, while the rest were subjected to Tobacco Etch Virus (TEV) cleavage.

A ratio of 1 µg His-tagged TEV protease (Molecular Cloning Laboratories) per 50 µg of recombinant protein (0.5 and 0.24 µg for RlpA-Δ81 and RlpA-SPOR, respectively) was used with an overnight incubation at 4 °C. Ni-NTA affinity chromatography purified both protein constructs by removal of the cleaved poly-His-tag and the His-tagged TEV protease. Aliquots of both poly-His-tagged and cleaved RlpA constructs (~3 and 1 mg mL^–1^ for RlpA-Δ81 and RlpA-SPOR, respectively) in 10 mM HEPES, 150 mM NaCl, 3 mM EDTA, 0.005% surfactant P20 pH 7.4 buffer were flash-frozen for storage at –80 °C (as described in the experimental). Both RlpA constructs stored in this solution were stable to thawing, and for days at 4 °C. Their solutions were successfully re-frozen and re-thawed. The molar concentration of the RlpA constructs in the solutions were determined from their A_280 nm_ absorbance (using the calculated ε_280 nm_ = 34,000 and 3000 L mol^–1^ cm^–1^ for RlpA-Δ81 and RlpA-SPOR, respectively) and cross-calibrated to the visible absorbance as determined by Bradford protein assay.

The recombinant plasmids for the binding partners that use pET28a(+) followed the same procedure for pET28a-*rlpA*, as described above, regardless of whether the transformed bacterium was *E. coli* BL21-star (DE3), C43 (DE3), or LEMO21 (Invitrogen). Each of the binding-partner recombinant plasmids that use pASK-IBA17k had their single colony of either *E. coli* BL21-star (DE3) or C43 (DE3) transformant cultured overnight in LB media containing 30 μg mL^–1^ of kanamycin (Sigma-Aldrich). This culture was transferred into 1 L of fresh LB media and was allowed to grow at 37 °C until an OD_600_ of 0.6. Protein expression was induced with 0.5 mM anhydrotetracycline hydrochloride (AHT, Abcam) at 16 °C overnight. The cell pellet was resuspended in 30 mL of lysis buffer [10 mM Tris pH 8.0, 150 mM NaCl, 0.05% Brij-35, 10 μg mL^–1^ of DNase I (bovine pancreas, Sigma-Aldrich), 10 μg/mL of lysozyme (chicken egg white, Sigma-Aldrich)]. Proteins were released from the cells by sonification on ice (10 × 1 min cycles with a 2 min rest). After 45 min of centrifugation at 18,000 × *g*, the supernatant was loaded onto 3 mL of Strep-Tactin resin (IBA Lifesciences). The resin was washed with 30 mL of lysis buffer and the protein was eluted with 15 mL of elution buffer (10 mM Tris·HCl, 150 mM NaCl, 2.5 mM desthiobiotin pH 8.0 buffer, Sigma-Aldrich). The fractions that contained the recombinant protein, as verified by SDS-PAGE gel, were collected and concentrated with an Amicon Ultra-Centrifugal Filter with either 10-kDa or 30-kDa cut-off. Size-exclusion chromatography was necessary for some proteins, typically a 300 mL 1.5 × 80 cm column of Sephacryl S-200 HR resin (GE Healthcare Life Sciences) equilibrated with 10 mM HEPES pH 7.4 buffer with 150 mM NaCl, 3 mM EDTA, and 0.005% P20 surfactant. Fractions with recombinant protein (as verified by SDS-PAGE gel) were concentrated (Amicon Ultra-Centrifugal Filter with a 10- or 30-kDa cut-off). Supplementary Table 1 identifies which binding partners required this additional purification step. All binding-partner proteins were stable at 4 °C and at –80 °C, and survived freeze-thaw cycles. The yields from 1 L cultures for the purified proteins are given on Supplementary Table 1.

### Protein preparations for the pulldown assays

*P. aeruginosa* PAO1 was grown in 500 mL LB medium to OD_600_ of 0.6 (mid-log phase) with shaking at 37 °C. Bacteria were harvested (5000 × *g*, 20 min, 4 °C) and washed once with 1× PBS buffer. Subsequently, the pellet was resuspended in 7 mL of 1× PBS buffer with Halt™ Protease Inhibitor Cocktail (100×) (Thermo Fisher Scientific) and transferred to a 15-mL Falcon tube. The bacterial suspension was sonicated (15 sec of sonication, 30 s rest on ice; 30 times). Cell debris was removed by centrifugation (8000 × *g*, 20 min, 4 °C). The supernatant was centrifuged (Sorvall XW-90 ultracentrifuge: 120,000 × *g* for 1 h at 4 °C). The supernatant (“soluble proteome”) was transferred to fresh tubes. The pellet (“membrane proteome”) was resuspended in cold 1× PBS and gently sonicated (5 s of sonication, 30 s rest on ice; five times). NP-40 (Thermo Fisher Scientific) detergent was added to give a 0.5% concentration. The suspension was rotated for 30 min at 4 °C. Both proteome samples were used immediately or stored at –80 °C until used. Total protein concentration was determined by BCA assay (Thermo Fisher Scientific).

### Pulldown experiments without crosslinking

Four tubes of RlpA-Δ32·Ni-NTA resin were prepared (two active, two control). In each of the active tubes a total of 50 µL of Ni-NTA (Macherey-Nagel) was incubated with 300 µg of *N*-terminally His-tagged RlpA-Δ32 by rotation for 1 h at 4 °C (total volume of 0.2 mL). The slurry was centrifuged for 1 min at 500 × *g* at 4 °C. The supernatant was collected. The resin was washed once with 500 µL of PBS buffer to remove any unbound RlpA-Δ32. The resin was recovered by centrifugation (1 min, 500 × *g*, 4 °C). The wash was discarded. A total of 3 mg of lysate (wild-type PAO1 strain, OD_600_ 1.0, exponential phase, 1 mL of a 3 mg mL^–1^ solution; soluble proteome in one tube and membrane proteome in the second tube) was incubated with the RlpA-Δ32·Ni-NTA resin (overnight, rotating at 4 °C). The resin was separated by centrifugation (1 min, 500 × *g*, 4 °C). The supernatant was discarded. The resin was washed twice with 400 µL of PBS buffer. Both washes were discarded. The complexes of RlpA-Δ32 with the partners were eluted with two washes of 300 µL of PBS buffer supplemented with 500 mM imidazole at 4 °C. Controls (absence of either the lysate or RlpA) were prepared and performed in parallel. Controls were appropriately used in-line with their respective cellular compartment. All fractions were analyzed by SDS-PAGE gels to confirm the elution of the complexes. The elution washes of both the RlpA-Δ32-complex samples (membrane- and soluble-fraction) and control without RlpA-Δ32 were analyzed by mass spectrometry.

### Pulldown experiments with cross-linking

His-tagged RlpA-Δ32 (300 µg) was incubated with 3 mg of lysate (wild-type PAO1 strain, OD_600_ 1.0, exponential phase, 1 mL of a 3 mg mL^–1^ solution membrane preparation or soluble-fraction) overnight, rotating at 4 °C. Subsequently, the mixture was incubated with 0.2 mL of a 5 mM solution (in DMSO) of the crosslinker bis(sulfosuccinimidyl)suberate (BS^3^, Thermo Fisher Scientific), by rotation at room temperature for 30 min. The reaction was quenched by setting the final buffer concentration to 50 mM Tris·HCl pH 8 buffer by addition of 100 μL of 500 mM Tris·HCl pH 8 (15 min reaction, rt). The mixture was then incubated with rotation with 50 µL of Ni-NTA resin for 1 h at 4 °C. The sample was centrifuged (1 min, 500 × *g*, 4 °C). The supernatant was removed, and the resin was washed twice with 400 µL of PBS buffer (all washes discarded). The RlpA·partner complexes were eluted with two washes of 300 µL of PBS buffer supplemented with 500 mM imidazole. The controls in the absence of either lysate or RlpA were prepared and performed in parallel. Controls were appropriately used in-line with their respective cellular compartment. All fractions were analyzed with SDS-PAGE gels to confirm the elution of the complexes. The elution washes of both the RlpA-complex samples (membrane- and soluble-fraction) and the control without RlpA-Δ32 were analyzed by mass spectrometry.

### Mass spectrometry analyses

Samples and controls were prepared for mass-spectrometry-based proteomics analysis as described previously^62,103^. Briefly, 400 μL elutions from the Ni-NTA bait experiments were precipitated in a 5 mL microcentrifuge tube using ten-fold excess of ice-cold acetone, pelleted, and dried. Pellets containing 20 μg protein were resuspended in 40 µL of 0.20 M triethylammonium bicarbonate (TEAB) buffer containing 10 mM dithiothreitol (DTT) with 6% SDS detergent. The suspensions were heated at 95 °C for 5 min. After cooling, samples were collected by brief centrifugation, and alkylated in the dark (reaction time of 20 min) by addition of 200 mM iodoacetamide in TEAB buffer to a final concentration of 20 mM. Samples were acidified with 13% H_3_PO_4_ to 1.2% (v/v), flocculated with 95:5 methanol/100 mM aqueous TEAB, and digested with trypsin using S-Traps, according to manufacturer’s protocol (Protifi, NY). Following digestion, eluted peptides were desalted for LC-MS analysis with a 1 mL-10 mg HLB-packed sorbent solid-phase extraction cartridge (Waters). Samples were dried in a MiVac (Genvac, MA) and stored at –20 °C until analysis.

Samples were resuspended in 25 µL of 0.2% aqueous formic acid and analyzed by nanoUHPLC-MS-MS/MS on a QExactive (Thermo) running a TOP15 Method. A 90-min gradient running at 900 nL/min was used as described^62,63^. RAW files were searched using MaxQuant and quantified using Label-Free Quantification [MaxLFQ]. The Pseudomonas FASTA database, concatenated with common contaminants, was obtained from The Pseudomonas Genome DB (the Cystic Fibrosis Foundation, Therapeutics)^64,65^. Data were filtered to a 1% protein false discovery rate (FDR) determined using target-decoy methods as in refs. ^62,103^. RAW and processed datafiles are available through the MassIVE/ProteomeExchange data repository (http://mchampion-nas.esc.nd.edu:5000/sharing/zcDQDUJQb Password PA01#2022). Protein quantification was used to measure fold-enrichment of RlpA-Δ32·bait samples compared to controls. The MaxLFQ ratio of RlpA-Δ32·bait-identified proteins divided by control was done as described^66,67^. Proteins that were detected exclusively and to high confidence (local FDR < 0.001) in bait samples were assigned a maximum fold-change of 64 (=2^6^). This reflects a practical limit of quantification and removes infinite ratios from the pool of data^68,69^. Novel proteins were further filtered with annotations in the Pseudomonas Genome Database with keywords ‘transglycosylase’ and then by matching the subcellular localization with the known transglycosylase.

### Fluorescent protein labeling and microscale thermophoresis

Fluorescent labeling of RlpA-Δ32 was required for MST. A 10-μM solution of purified RlpA-Δ32 was allowed to react (1 h in the dark, rt) with 10 μM amine reactive RED-NHS dye (Nanotemper Technologies) dissolved in dimethylformamide (DMF, 200 μL final volume of mixture with final 4.6% of DMF). The incubated mixture was put through an Econo-Pac chromatography column (Bio-Rad) by gravity flow to separate the free dye from the modified RlpA-Δ32. The fractions that contained the fluorophore-tagged RlpA-Δ32 (as verified by the A_260_/A_280_ measured on an Implen NanoPhotometer NP80) were kept at 4 °C for immediate use or stored at –80 °C. The sample survived freeze-thaw cycles.

We used a fixed concentration of 5 nM modified RlpA-Δ32 and 16 samples of increasing (progressively doubling) concentration for the partner protein. Each sample was loaded into a Monolith NT.115 premium capillary (Nanotemper Technologies) by capillary action. Binary MST runs imply two biomolecules being tested: a target at a fixed concentration with a ligand at a progressively doubling concentration. Ternary MST runs imply three biomolecules being tested; a target set at a fixed concentration, another biomolecule also set at a fixed concentration (at a concentration that allows for at least 80% saturation of the fluorescently tagged biomolecule, calculated by the following formula: Amount of biomolecule for target complex (M) = ((*K*_D_ · (% bound/100) · Target concentration) + ((% bound/100) · Target concentration^2^)) – (((% bound/100)^2^ · Target^2^)/Target concentration – ((% bound/100) · Target concentration))), and a ligand at a progressive doubling concentration. In the present report, we tested ternary MST runs that involved 5 nM of RlpA-Δ32 as target, 55 nM of MltF2 as the biomolecule for target complex, and progressively doubling of a partner protein (concentrations tested of partner protein can range from 0.1 nM to 50 μM, depending on the specific partner protein used). Prior to any MST runs, all proteins were centrifuged at 17,000 × *g* at 4 °C for 10 min. All MST experiments were performed in triplicate. The MST was made available by the Warren Center for Drug Discovery at the University of Notre Dame.

### Carboxymethylated dextran chip ligand immobilization and surface-plasmon resonance

Analysis of biomolecule interaction started with the immobilization of RlpA-Δ32 onto a carboxymethylated dextran (CM4) chip (Cytiva). The CM4 sensor chip (Cytiva) contains two flow cells running in succession, they will be known as flow cell (FC) 1 and FC 2. FC 2 was pretreated with 10 μL of the recommended running buffer (10 mM HEPES pH 7.4, 150 mM NaCl, 3 mM EDTA, 0.005% Surfactant P20) at 10 μL min^–1^. FC 2 is activated with 100 μL of equimolar NHS (final concentration 50 μM in deionized water, Cytiva) and EDC (final concentration 240 μM in deionized water, Cytiva) at 10 μL min^–1^. Next, a flow of 100 μL with 0.25 μM RlpA-Δ32 in 10 mM sodium acetate pH 5.0 buffer at 10 μL min^–1^ is fixed to FC 2 as the surface lysines of the protein react with the NHS active ester of the chip. A flow of 75 μL with 1 M ethanolamine hydrochloride-NaOH pH 8.5 buffer (Cytiva) at 10 μL min^–1^ quenched any unreacted NHS ester. The same immobilization process was used on FC 1, where the protein GFP-STT (IBA Lifesciences) was bound to prevent nonspecific binding to the reference cell. The chip was equilibrated for the protein–protein assay by a 50 μL flow of running buffer at 10 μL min^–1^ (10 mM HEPES pH 7.4, 150 mM NaCl, 3 mM EDTA, 0.005% Surfactant P20). Amine-coupling is exploited here for adhering both GFP (IBA Lifesciences) and RlpA-Δ32 to FC 1 and FC 2 on the chip, respectively. The same amine-coupling immobilization procedure was used for the RlpA-Δ81 and RlpA-SPOR constructs in FC 2 for their own respective sensor chips. A Biacore X100 analytical system (GE Healthcare, operating with the Biacore X100 Control Software and Biacore X100 Evaluation Software) was used. The analysis of binary-protein complexation was carried out by injecting the analyte (binding partner) in running buffer for 3 min over the GFP-STT (IBA Lifesciences) and RlpA-Δ32 immobilized CM4 chip, then washing the chip with running buffer for 10 min at 5 μL min^–1^ flow rate. The CM4 chip was regenerated by a 10 μL pulse of 0.10 M NaOH at 10 μL min^–1^. Regeneration in the context of this section equates to eliminating any and all analyte presently bound to a ligand that is covalently bound to the chip’s surface once SPR cycle runs have been completed. All experiments were conducted at 25 °C. Each SPR run had at least five different concentrations of analyte spread out across two- to fivefold dilutions. Initial analyte concentrations span 1–100 μM, depending on the analyte. Double-blank subtraction was used for all SPR experiments. All of the displayed sensorgrams are [(FC 2) – (FC 1)] data and are measured in response units (RU). Curves were fit to a 1:1 stoichiometry.

### Isothermal-titration calorimetry

ITC experiments were performed using a MicroCal PEAQ-CAL instrument (Malvern) from the Biophysics Instrumentation Core facility at the University of Notre Dame. All protein samples used for ITC experiments were prepared in 50 mM Tris, 300 mM NaCl, and pH 8.0 degassed buffer prior to measurements. Using a micro-syringe, 2 μL of each protein was added at intervals of 200 s into the cell with stirring (750 rpm) at 25 °C. Controls devoid of tested protein within the sample cell for each ITC experiment were run. All data were fit to the one-set-of-sites model. The curve fittings were generated using GraphPad Prism 5. Data are expressed as the mean ± SEM from triplicate experiments. The ITC thermograms are representative of triplicate experiments.

### Analytical ultracentrifugation

Spin samples at 15,000 RPM for 10 min at 4 °C. Sedimentation velocity experiments were performed in a ProteomeLab XL-I AUC (Beckman-Coulter) at 42,000 RPM and 20 °C using an AN-50 Ti rotor (Beckman-Coulter). Double-sector cells equipped with 1.2 cm charcoal-epon centerpieces (Beckman-Coulter) and sapphire windows were used. Samples were allowed to equilibrate for 75 min prior to starting the run. Absorbance at 280 nm was measured with 0.003 cm radial step size. The partial specific volume of RlpA-Δ32 and RlpA-SPOR were assumed to be 0.73 mL/g. Buffer density and viscosity at 20 °C were calculated using SEDNTERP. Data were analyzed using the c(*s*) distribution and c(M) distribution models in Sedfit. Data were plotted using GUSSI.

### Statistics and reproducibility

Statistical analyses were conducted on graphs and tables presented within this manuscript. Standard deviation (SD) and standard error of the mean (SEM) were implemented in their respective analyses. They are denoted as such within the figure and table text. All statistical analyses were done in triplicate experiments. The reproducibility of all experiments presented in the manuscript are robust and repeatable.

### Reporting summary

Further information on research design is available in the Nature Portfolio Reporting Summary linked to this article.

## Supplementary information

Supplementary Information

Description of Additional Supplementary Files

Supplementary Data 1

Reporting Summary

## Data Availability

The authors declare that the data supporting the findings of this study are available within the article and it’s Supplementary Information and Supplementary Data files. A reporting summary for this paper is available as a Supplementary Information file. Source data (Supplementary Data) are provided with this paper.
