# Supplementary material for: In vitro studies of the protein-interaction network of cell-wall lytic transglycosylase RlpA of Pseudomonas aeruginosa
Source: Commun Biol. 2022 Nov 30;5:1314. doi: 10.1038/s42003-022-04230-x (PMC9712689; doi:10.1038/s42003-022-04230-x)
Supplement: Supplementary file 1 — Supplementary Information [file 42003_2022_4230_MOESM1_ESM.pdf]

Supplementary Information

In vitro Studies of the Protein-Interaction Network of Cell-Wall Lytic Transglycosylase RlpA of *Pseudomonas aeruginosa*

Luis F. Avila-Cobian[1], Stefania De Benedetti[1], Choon Kim[1], Rhona Feltzer[1], Matthew M. Champion[1], Jed F. Fisher[1], and Shahriar Mobashery[1*]

[1]Department of Chemistry and Biochemistry, University of Notre Dame, Notre Dame, IN, 46556, USA. Correspondence and requests for materials should be addressed to S.M. (email: mobashery@nd.edu)

Table of Contents

	Content	*Page(s)*
**1**	**Supplementary Fig. 1.** Primary structures of RlpA constructs.	S3
**2**	**Supplementary Fig. 2.** Primary structures of LTs used for pulldown procedure.	S4–S6
**3**	**Supplementary Table 1.** Cloning, expression, and purification details for RlpA constructs and binding partners.	S7–S8
**4**	**Supplementary Table 2.** Putative partners (prey) for all 11 Pseudomonas LTs (bait) and their enrichment in the pulldown procedure.	S9–S14
**5**	**Supplementary Table 3.** List of the putative partners for all 11 LTs with attributed name, functions, and localization	S15 – S17
**6**	**Supplementary Fig. 3**. MST traces and dose-response curves of tested binary RlpA-Δ32 combinations.	S18 – S22
**7**	**Supplementary Fig. 4.** MST traces and dose-response curves of tested ternary RlpA-Δ32 combinations.	S23 – S27
**8**	**Supplementary Fig. 5.** Determination of the availability of binding for presumed negative controls against RlpA-Δ32.	S28
**9**	**Supplementary Fig. 6.** SPR sensorgrams of tested binary RlpA-Δ32 interactions.	S29 – S31
**10**	**Supplementary Fig. 7.** SPR sensorgrams and kinetics of tested ternary RlpA-Δ32 interactions.	S32 – S33
**11**	**Supplementary Fig. 8.** SPR sensorgrams of tested RlpA-SPOR interactions.	S34 – S35
**12**	**Supplementary Fig. 9.** SPR sensorgrams of tested RlpA-Δ81 interactions.	S36 – S37
**13**	**Supplementary Fig. 10.** RlpA operons in *Escherichia coli* and *Pseudomonas aeruginosa*	S38
**14**	**Supplementary Fig. 11.** Primary structures of SPOR-domain containing proteins within *Pseudomonas aeruginosa* PAO1.	S39
**15**	**Supplementary Fig. 12.** AUC c(*s*) analyses on the monomeric state of the RlpA-constructs.	S40

Construct	Amino Acid Sequence	AAN
**RlpA-Δ32**	MGSSHHHHHHSSGLVPRGSHMQQPARQAGISGPGDYSRPHRDGAPWWDVDVSRIPDAVPMPHNGSVKANPYTVLGKTYYPMNDARAYRMVGTA<br>SWYGTKFHGQATANGETYDLYGMTAAHKTLPLPSYVRVTNLDNGKSVIVRVNDRGPFYSDRVIDLSFAAAKKLGYAETGTARVKVEGIDPVQWWAQR<br>GRPAPMVLAQPKQAVAQAAPAAAQTQAVAMAQPIETYTPPPAQHAAAVLPVQIDSKKNASLPADGLYLQVGAFANPDAAELLKAKLSGVTAAPVFISS<br>VVRNQQILHRVRLGPIGSADEVSRTQDSIRVANLGQPTLVRPD	93<br>190<br>288<br>331
**RlpA-Δ81**	MGSSHHHHHHSSGENLYFQGHMYTVLGKTYYPMNDARAYRMVGTASWYGTKFHGQATANGETYDLYGMTAAHKTLPLPSYVRVTNLDNGKSVIVR<br>VNDRGPFYSDRVIDLSFAAAKKLGYAETGTARVKVEGIDPVQWWAQRGRPAPMVLAQPKQAVAQAAPAAAQTQAVAMAQPIETYTPPPAQHAAAV<br>LPVQIDSKKNASLPADGLYLQVGAFANPDAAELLKAKLSGVTAAPVFISSVVRNQQILHRVRLGPIGSADEVSRTQDSIRVANLGQPTLVRPD	95<br>190<br>283
**RlpA-SPOR**	MGSSHHHHHHSSGENLYFQGHMADGLYLQVGAFANPDAAELLKAKLSGVTAAPVFISSVVRNQQILHRVRLGPIGSADEVSRTQDSIRVANLGQPTLVR<br>PD	99<br>101

Supplementary Fig. 1. Primary structures of RlpA constructs. Constructs were all prepared based on the PA4000 sequence within *P. aeruginosa* (PAO1). RlpA-Δ32 has *N*-terminal poly-Histidine-tag, linker, and thrombin cleavage site sequences preceding start of gene sequence. RlpA-Δ81 and RlpA-SPOR have *N*-terminal poly-Histidine-tag, linker, and TEV cleavage-site sequences preceding start of gene sequence. Recombinant tags were cleaved off of each RlpA construct, with their appropriate cleavage enzyme, prior to any downstream experiments. All sequences were retrieved from The Pseudomonas Genome Database (Cystic Fibrosis Foundation, Therapeutics). The lytic transglycosylase domain is highlighted in blue lettering, when present. The catalytic aspartate in the lytic transglycosylase domain is highlighted in red lettering, when present. The expansin-type domain is highlighted in orange lettering, when present. The SPOR domain is highlighted in green lettering. AAN denotes number of amino acids in each line of the chart.

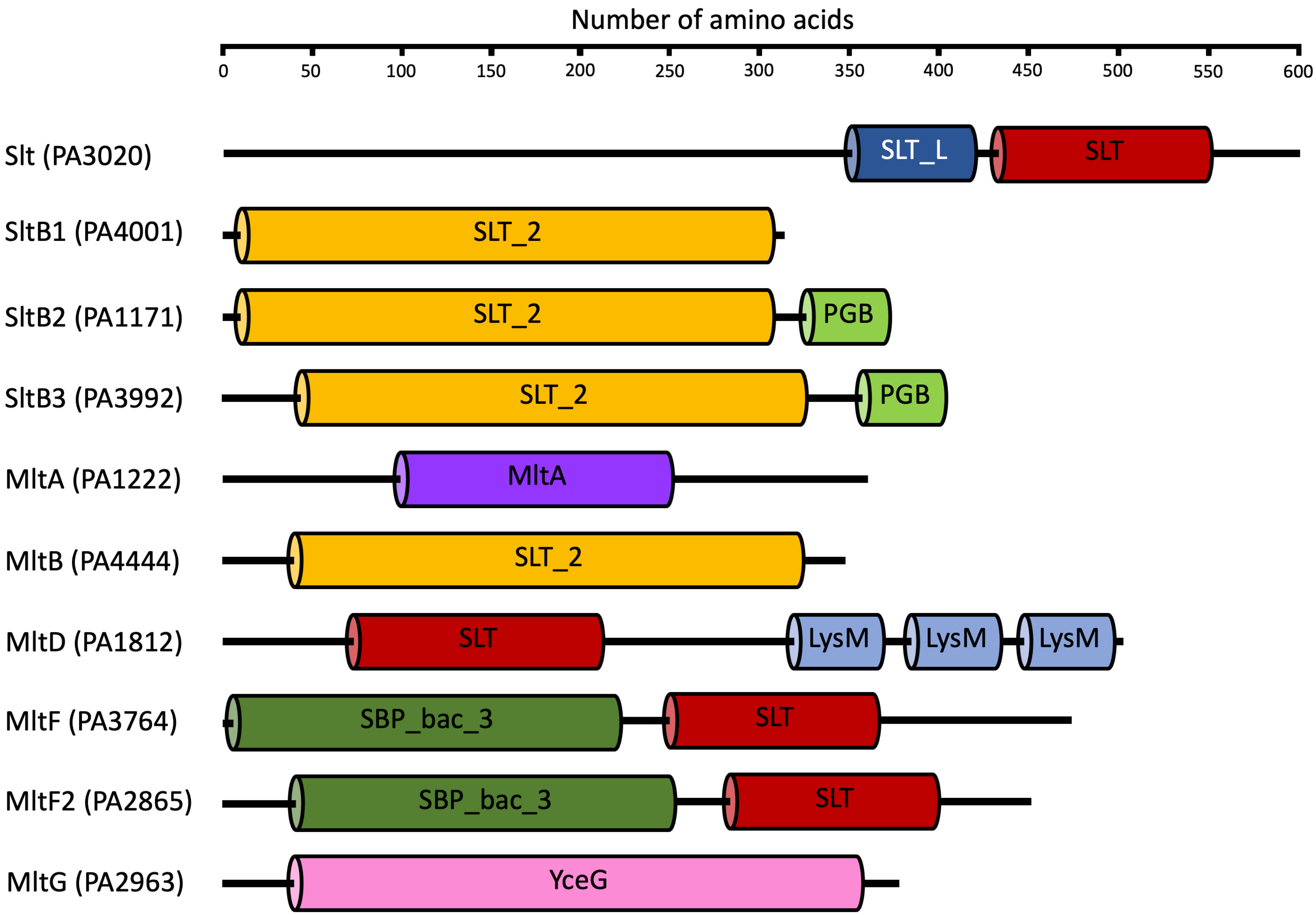
Number of amino acids
0
50
100
150
200
250
300
350
400
450
500
550
600
Slt (PA3020)
SLT_L
SLT
SltB1 (PA4001)
SLT_2
SltB2 (PA1171)
SLT_2
PGB
SltB3 (PA3992)
SLT_2
PGB
MltA (PA1222)
MltA
MltB (PA4444)
SLT_2
MltD (PA1812)
SLT
LysM
LysM
LysM
MltF (PA3764)
SBP_bac_3
SLT
MltF2 (PA2865)
SBP_bac_3
SLT
MltG (PA2963)
YceG

Construct	Amino Acid Sequence	AAN
**Slt (PA3020)**	MGSSHHHHHHSSGLVPRGSHMASLTEQRRLYDQAKAALAKGNSAPYMASRSALRDYPLEPYLAYDELTHRLKSASNEEVERFLTEHGDLPQIGWLKLR<br>WLRLLADRGDWKTFVNYYDPKLNFTELDCLYGQYQLGHGQKAEGYATSERLWLVGKSQPAACDTLFGLWQGEGQLTEEKVWKRLKLAAEARNYSLAS<br>HLAQRLPTLGNQGALMVSVAQNPAQLSQTGRFSQRDHATADVVGLGLRRLARQDPEKALSLLDYYSSALPFSSDEKVAIAREIGLSLAKRFDPRALPLMT<br>QYDPGLRDNTVTEWRTRLLLRLGRWDEAYALTRKLPQDLAATSRWRYWQARSLQLAQPNSKEPIALYQKLAGERDFYGFLAADRLSVPYKLGNRPAHID<br>PRVLQRVRNAASTRRAMEFFNRGEVINARREWYHAARLFDRDELIAQARLAYDMQWYFPAIRSISQAQYWDDLDIRFPMAHRATLVREAKNRGLHSS<br>WIFAITRQESAFMSDARSGVGATGLMQLMPGTAKETSRKFGIPLASTQQLIVPDVNIRLGAAYLSQVHSQFNGNRVLASAAYNAGPGRVRQWLKDTRH<br>LAFDVWIETIPFDETRQYVQNVLSYAVIYGQKLNAPQPIVDWHERYFDDF	98<br>195<br>295<br>394<br>491<br>589<br>639
**SltB1 (PA4001)**	MGSSHHHHHHSSGLVPRGSHMGDYDGSPQVAEFVSEMTRDYGFAGEQLMGLFRDVNRKQSILDAISRPAERVKQWKEYRPIFISDARISRGVDFWNK<br>HAEDLARAEKEYGVPAEIIVSIIGVETFFGRNTGSYRVMDALSTLGFDYPPRADFFRKELREFLLLAREQQVDPLSLTGSYAGAMGLPQFMPSSFRAYAVDF<br>DGDGHINIWSDPTDAIGSVASYFKQHGWVTGEPVVSVAEINDESAESAVTRGVDPTMSLGELRARGWRTHDALRDDQKVTAMRFVGDKGIEYWVGL<br>PNFYVITRYNRSAMYAMAVYQLAGEIARARGAH	97<br>199<br>295<br>328
**SltB2 (PA1171)**	MGSSHHHHHHSSGLVPRGSHMQPDASSFPSCLAGLQKKAQAQGISADSYERFTSGLQADLSVLDLLDAQPEFTTPLWDYLAGLVDEQRVSDGKAMLA<br>QHDKLLDQVAARYGVDKYTVVAVWGVESDYGRIFGKRPLLTSLSTLSCYGRRQSFFQGEFLATLKLLQAGDIRDAGITGSWAGAFGHTQFMPSTYARIAV<br>DFDGDGRRDLVGSVPDALGSTANYLKKAGWRTGQPWGYEVKVPADFPASLAGRGKRQPLSAWVARGVRRVDGQPLPGGDEKAAILLPAGAQGPAFL<br>VYRNYDAIYSYNAAESYALAIALLSDRLRGGSGLVASWPTDDPGISRLERKQLQKALLARGYDIGEADGLIGTSTRKAIQAEQKRLGLTPADGRAGRKILEAL<br>KGAQP	97<br>197<br>293<br>396<br>401
**SltB3 (PA3992)**	MGSSHHHHHHSSGLVPRGSHMQKNPTVEYNQPAAPLQTKAPFSGAGPAASVPAGAPNEAQPGQSFEQWRDAFRQQALAGGIDAQTFDRAFAGVQ<br>PDPAVVEADRSQPEFTRPVWKYLEGALDPLRVRQGQARLAQHARILGEVDARYAVDADAVVAIWGMESNYGSHMGNKNVIRSLATLAYEGRRPEFAH<br>AQLLAALKILQHGDVPASFMIGSWAGAMGQTQFIPTTHNQYAVDFDGDGKRDIWGSPGDALASTANYLKASGWIAGQPWGFEVRLPAGFDYSLAELT<br>IRKPLGEWQGMGVQGVNGGPLPSGLSGEQASLLLPAGHRGPAFLVLHNFRAILKYNNSSAYALAVGLLADSFKGGGRIVGAWPLEDVPLSRSQRIELQR<br>QLAARGHDPGAVDGIIGANTRKAIRACQQEFGWPADGYPTPALLDRLRTP	94<br>191<br>288<br>387<br>437
**MltA (PA1222)**	MGSSHHHHHHSSGLVPRGSHMCDDGKKEPPPKPAEVTTYNSVPWDALPATSDADLLAGFNAWRSACARLAKDPVWGEPCASATTVAADPTAVRAFL<br>QERMQVYSLRSSSNGDQGLITGYYEPVYHGSLSQGEKTPVPVYGVPDDLVVVALESVYPELKGKRLRGRLEGRVLKPYDDAATIRDNGSSAPVLAWLGDP<br>MDLQFLQIQGSGRIQLEDGRQLRIGYGDQNGHPYKPVGRWLVEQGLVPKEEISMKRIRDWAEANPQRVSELLASNPSFVFFSLRPDSDEGPRGSLNVPL<br>TDGYSVAIDRKVIPLGSLMWLSTTRPDDGSAVVRPVAAQDTGGAIVGEVRADLFWGTGDAAGELAGHMKQPGRLWLLWPKGAPLPAS	96<br>196<br>295<br>382
**MltB (PA4444)**	MGSSHHHHHHSSGLVPRGSHMSSEPTPPPKPAAKPQARTVISPRPVRQSVQPILPLRGDYANNPAAQHFIDRMVSQHGFNRQQLHDLFAQTQRLDW<br>VIRLMDRQAPTYTPPSGPNGAWLRYRKKFVTPGNVQNGVLFWDQYETDLQRASRVYGVPPEIIVGIIGVETRWGRVMGKTRIIDALSTLSFSYPRRAEFF<br>SGELEQFLLQARKEGTDPLALRGSYAGAMGYGQFMPSSFTKYAVDFDGDGHIDLWNPRDAIGSVANYFKQHGWVSGDRVAVPASGRAPSLEDGFKTL<br>YPLDVLASAGLRPQGPLGGHRQASLLRLDMGRNYQYWYGLPNFYVITRYNHSTHYAMAVWELGKEVDRVRHRSVVRQD	96<br>196<br>293<br>371
**MltD (PA1812)**	MGSSHHHHHHSSGLVPRGSHMSGQIKGEAQAKAARPGARAVDVQFNPSWLHTQPGQNAAYNDIWDRMRDGFQLQDAISTNPRIERQRLWFLSNQ<br>SFLEQSSARGSLYMHYVVERLEERNMPLELALLPVIESAYNPFALSRSNAAGLWQFIPATGQHFNLRQTNFYDGRRDITASTNAALTYLERLHDMFNGD<br>WMLALAAYNAGEGTVSRAIERNEKLGLPTDYWNLPLPQETQDYVPKLLALSQIVMAPDSYGISLNPINNEPYFQAVRVKRGIDLSSVAALANLDEDELYQ<br>LNPAYKRRVTMDGPQQLLVPMEKAAFLTASLDTLKPKEVTAWQQYRVRSGDSLHSIANRYRITVAELKSANRLSSNHLRKGQQLSIPGQIAGGAVKPVY<br>QQLARQASTPARTRSYKVKNGDSLWQIARNNGVDVNDLKRWNGLDKHALKVGQTLKLQGGTQALAARKGNAAGKRDSATYYKVKQGDSMYLIAKRF<br>NVEMKHLQRWNPRSKQALKPGQTLTLYLDTASR	94<br>193<br>293<br>392<br>488<br>521

Construct	Amino Acid Sequence	AAN
**MltF (PA3764)**	MGSSHHHHHHSSGLVPRGSHMRITGQPDNWHREQPETRDLASIRRAGVLKVLVNQSRNSSGEVKGEPIGVEYRRLRAFEQYLNSRSPSARNLTLKLIPKP<br>KDQLLAALQRGEGDLVAPGELLPAHDGLQVSPSAPVRADVPLVLVARKGNRRYTRLEQLSGRTIPLPAGSAAEGAIELVNQRLAQRRMAPIRIEKLDASL<br>AVEDVMEMVQAGILGFTVVEQPIAERWAKVLPKLRVDRHLVLDNRADMAWYVRRDASTLRATIDRFLADYRAPADQDVAFQRVYRRAYKVRNPLGA<br>ADRKRLEAVRPLLQRYARQSSMDWLALAAVAYKESHLNPKARGSGGASGLMQITPAAARSVGVGNVHDKDSNVLAASRYLTKIRKQFFSSKHLDERERL<br>AFTLAAYNMGPERVQNLRTQARRRGLDPNRWFFQVERVAAEEIGMGVVSYVSSVNKYYLAYERERVRLEPGVRAKTATAQK	100<br>200<br>296<br>395<br>476
**MltF2 (PA2865)**	MGSSHHHHHHSSGLVPRGSHMAKRFAERLGVELKIETADNLDDLYAQLSREGGPALAAAGLTPGREDDASVRYSHTYLDVTPQIIYRNGQQRPTRPEDL<br>VGKRIMVLKGSSHAEQLAELKKQYPELKYEESDAVEVVDLLRMVDVGDIDLTLVDSNELAMNQVYFPNVRVAFDFGEARGLAWALPGGDDDSLMNEV<br>NAFLDQAKKEGLLQRLKDRYYGHVDVLGYVGAYTFAQHLQQRLPRYESHFKQSGKQLDTDWRLLAAIGYQESLWQPGATSKTGVRGLMMLTNRTAQA<br>MGVSNRLDPKQSIQGGSKYFVQIRSELPESIKEPDRSWFALAAYNIGGAHLEDARKMAEKEGLNPNKWLDVKKMLPRLAQKQWYAKTRYGYARGGET<br>VHFVQNVRRYYDILTWVTQPQMEGSQIAESGLHLPGVNKTRPEEDSGDEKL	99<br>196<br>293<br>390<br>441
**MltG (PA2963)**	MGSSHHHHHHSSGLVPRGSHMQQQRALEQPLQLTEERLLDVSSGSTPGGMLARLEQEKVLHGAFWLRLYWRFNLPGQALHSGEYRLLPGMKGADLL<br>ELWREGEVVQYSLTLVEGWSFRQVREALARQGKLEQTLAGLSDGEIMQRLGKPDEVAEGRFFPDTYRYTRGMRDIDILRKAYQRMQTILAKEWDGRSQ<br>DLPYRDAYQALIMASLVEKETGVPEERSQIAGVFVRRLQRGMLLQTDPTVIYGMGERYNGKITRADLREPTPYNTYVVPGMPPTPIALAGREAIRAALHP<br>AEGETLYFVARGDGSHVFSSSLDEHNKAVREYQLKRRSDYRSSPAPITPPPQ	96<br>194<br>294<br>346

Supplementary Fig. 2. Primary structures of LTs used for pulldown procedures. Constructs were all prepared based off the *P. aeruginosa* PAO1 genome. *N*-terminal poly-Histidine-tag, linker, and thrombin cleavage site sequences precede the start of all gene sequences in the table above and are illustrated as such. All sequences were retrieved from The Pseudomonas Genome Database (Cystic Fibrosis Foundation, Therapeutics). The SLT_L domain is highlighted in navy-blue lettering; amino acids 404–470 for Slt. The SLT_2 domain is highlighted in light-orange lettering; amino acids 40–330 for SltB1, amino acids 25–316 for SltB2, amino acids 75–369 for SltB3, amino acids 59–352 for MltB. 'PGB' in light green denotes peptidoglycan binding domain; amino acids 339–393 for SltB2, amino acids 391–445 for SltB3. The MltA domain is highlighted in purple lettering; amino acids 126–281 for MltA. The SLT domain is highlighted in red lettering; amino acids 485–596 for Slt, amino acids 110–215 for MltD, amino acids 301–405 for MltF, amino acids 310–418 for MltF2. The LysM domains are highlighted in blue lettering; amino acids 349–392, 419–462, and 484–527 for MltD. The Bacterial extracellular solute-binding proteins Family 3 (SBP_bac_3) domain is highlighted in dark green lettering; amino acids 33–269 for MltF, amino acids 66–271 for MltF2. The YceG domain is highlighted in pink lettering; amino acids 56–328 for MltG.

Supplementary Table 1. Cloning, expression, and purification details for RlpA constructs and binding partners.

Name	Locus tag	Gene cloned (AA)	Molecular Weight (Da)	Plasmid	Primers and Restriction Sites	*E. coli* strain	SEC	Protein Amount
RlpA-Δ32	PA4000	32-342	35,875	pET28a(+)	rlpA_Fwd_NdeI (5’-atatacatatgaaggcgccccagcagccggcca-3’)<br>rlpA_Rev_XhoI (5’-atatactcgagtcagtcggggcgtaccagcgtcgg-3’)	BL21 star	X	30 mg
RlpA-Δ81	PA4000	82-342	30,308	pET28aTEV	Δ81_Fwd_NdeI (5’- atatatcatatgtacaccgtgctgggc-3’)<br>Δ81_Rev_XhoI (5’- atatatctcgagtcagtcggggcgtac-3’)	BL21 star	x	25 mg
RlpA-SPOR	PA4000	264-342	11,249	pET28aTEV	SPOR_Fwd_NdeI (5’- atatatcatatggccgatggcctgtatc-3’)<br>SPOR_Rev_XhoI (5’- atatatctcgagtcagtcggggcgtac-3’)	BL21 star	x	12 mg
SltB1	PA4001	34-340	36,782	pET28a(+)	sltB1_Fwd_NdeI (5’-ccccccatatgggggactacgacggctcgccgcaa-3’)<br>sltB1_Rev_XhoI (5’-cttgctcgagtcaatgggcacctcgcgcgcgg-3’)	BL21 star	X	65 mg
SltB2	PA1171	19-398	43,141	pET28a(+)	sltB2_Fwd_NdeI (5’-ttaattcatatgcaacccgacgcctcgagcttccct-3’)<br>sltB2_Rev_HindIII (5’-taataagctttcagggctgggcgcccttcagc-3’)	BL21 star	X	43 mg
SltB3	PA3992	33-448	46,884	pET28a(+)	sltB3_Fwd_NdeI (5’-ccccccatatgcagaagaatccgacagtcgaatacaac-3’)<br>sltB3_Rev_XhoI (5’-ccttctcgagctatggcgtccgcaggcggtc-3’)	BL21 star	X	24 mg
MltA	PA1222	25-385	41,395	pET28a(+)	GenScript, Optimized for *E. coli* expression	BL21 star	X	26 mg
MltB	PA4444	18-367	41,841	pET28a(+)	mltB_Fwd_NheI (5’-ccccccatatgagcagcgaaccgacgccacca-3’)<br>mltB_Rev_XhoI (5’-tcgcctcgagctaatcctgcctgacgacggagcg-3’)	BL21 star	X	65 mg
MltD	PA1812	35-534	58,543	pET28a(+)	mltD_Fwd_NdeI (5’- ggggggcatatgagcggccagatcaagggggagg-3’)<br>mltD_Rev_HindIII (5’-ggggaagctttcagcggctcgcggtgtccaggta-3’)	BL21 star	X	33 mg
MltF	PA3764	40-490	53,326	pET28a(+)	mltF_Fwd_NdeI (5’-gtattacatatgcagaaggagggcgtactgcgcgt-3’)<br>mltF_Rev_XhoI (5’-ctatctcgagctagagtttctcgtcgccgctgtc-3’)	BL21 star	X	30 mg
MltF2	PA2865	22-476	53,588	pET28a(+)	mltF2_Fwd_NheI (5’-taatgctagccgcatcaccggccagccgctgtc-3’)<br>mltF2_Rev_XhoI (5’-ccccctcgagttatttttgagccgtcgccgtcttc-3’)	BL21 star	X	8 mg
PBP1a	PA5045	36-822	87,238	pET28a(+)	pbp1a_Fwd_NdeI (5’-cgcggcagccatatggtggaagctctgcgtaacgtccagc-3’)<br>pbp1a_Rev_XhoI (5’-gtggtgctcgagctatcagaacaggtcgatcggcgcgcct-3’)	BL21 star	X	29 mg
PBP1b	PA4700	46-774	80,413	pASK-IBA17k	pbp1b_Fwd_KasI (5’-ctttattttcagggcgccgatgccgtggtgcaggagaa-3’)<br>pbp1b_Rev_KpnI (5’-gagggatccccgggtacctcaattcagccagccacgtacc-3’)	C43	X	12 mg
PBP4	PA3047	23-476	49,640	pET28a(+)	GenScript, Optimized for *E. coli* expression	LEMO21	✓	7 mg
PBP7	PA0869	25-310	31,513	pASK-IBA17k	pbp7_Fwd_KasI (5’-ctttattttcagggcgccccgccgcccaaggccgggct-3’)<br>pbp7_Rev_KpnI (5’-gagggatccccgggtacctcagcgtacggcctgggccaccggc-3’)	C43	X	10 mg
TypA	PA5117	32-650	69,116	pASK-IBA17k	typA_Fwd_KasI (5’-atatatggcgccctgcgcaacatcgcc-3’)<br>typA_Rev_KpnI (5’- tatataggtaccttagaccttgctgcgctc-3’)	BL21 star	X	32 mg
HP	PA0788	64-1041	109,940	pET28aTEV	0788_Fwd_EcoR1 (5’-gcgcgcgaattcggaagcgcacacctcgcg-3’)<br>0788_Rev_XhoI (5’-gcgcgcctcgagctacaggcggccgatctg-3’)	BL21 star	X	X
SlyB	PA1053	20-154	13,715	pET28aTEV	slyB_Fwd_KasI (5’-ctttattttcagggcgccggctgccaatccagcctcac-3’)<br>slyB_Rev_KpnI (5’-gagggatccccgggtacctcagtgggcgacgcggc-3’)	BL21 star	X	18 mg
HP	PA2854	24-323	34,800	pET28aTEV	2854_Fwd_KasI (5’-tttcagggccatatgctggaactgcagctc-3’)<br>2854_Rev_KpnI (5’-caccacgagctcgattcagggcgtaagctg-3’)	BL21 star	✓	22 mg
HP	PA4063	18-196	19,416	pET28aTEV	4063_Fwd_KasI (5’-tttcagggccatatgcatgacgaccacgac-3’)<br>4063_Rev_KpnI (5’-caccacgagctcgatctacagcttcagctc-3’)	BL21 star	X	27 mg
PilA	PA4525	20-149	12,463	pET28aTEV	pilA_Fwd_KasI (5’-ctttattttcagggcgcctatcagaactatgttgcgcgttcggaaggt-3’)<br>pilA_Rev_XhoI (5’-gcgcgcctcgagttagttatcacaacctttcggagtgaacat-3’)	BL21 star	X	15 mg

MigA	PA0705	32-299	41,396	pASK-IBA17k	migA_Fwd_KasI (5’-atatatatggcgccgtcgtagcgcc-3’)<br>migA_Rev_KpnI (5’-atatatggtacctcagctctcgtgatggaga-3’)	BL21 star	X	X
Wzz	PA3160	52-320	33,259	pET28aTEV	wzz_Fwd_NdeI (5’-tttcagggccatatgtatgaggcccgcatagcgat-3’)<br>wzz_Rev_XhoI (5’-gtggtgctcgagctattacatcgctctccttggacgaa-3’)	BL21 star	X	13 mg
AlgO	PA3257	43-709	77,206	pET28a	algO_Fwd_NdeI (5’-cgcggcagccatatgcagaattcctgggacagcctgcag-3’)<br>algO_Rev_XhoI (5’-gtggtgctcgagctatcagtgcttggccaccgccgagtt-3’)	C43	X	9 mg
LptE	PA3988	19-207	23,292	pET28aTEV	lptE_Fwd_NheI (5’- cgcggcagccatatggcctgcggcttccaactgcg-3’)<br>lptE_Rev_XhoI (5’- gtggtgctcgagctatcacggggtggggaactcga-3’)	BL21 star	✓	17 mg
PmrB	PA4777	X	X	X	N/A<br>N/A	X	X	X
PilO	PA5042	32-207	19,855	pASK-IBA17k	pilO_Fwd_KasI (5’-atatatggcgccggctacaacttccatctgagtga-3’)<br>pilO_Rev_KpnI (5’-atatatggtacctcatttcttcagccccttgtc-3’)	BL21 star	X	21 mg
PvdL	PA2424	X	X	X	N/A<br>N/A	X	X	X

The name, locus tag, section of gene cloned (AA: amino acids included), molecular weight, plasmid, primers, restriction sites, *E. coli* strain, SEC: size-exclusion chromatography, and protein amount (per 1 L growth LB culture) are shown. HP: hypothetical protein, ✓: attempted. X: not attempted.

Supplementary Table 2. Putative partners (prey) for all 11 Pseudomonas LTs (bait) and their enrichment in the pulldown procedure.

Partners (prey)	Lytic transglycosylases (bait)																					
Locus tag	MltA		MltB		MltD		MltF		MltF2		MltG		RlpA		Slt		SltB1		SltB2		SltB3	
	Mem	Sol	Mem	Sol	Mem	Sol	Mem	Sol	Mem	Sol	Mem	Sol	Mem	Sol	Mem	Sol	Mem	Sol	Mem	Sol	Mem	Sol
**PA0041**	-	-	-	-	-	-	-	-	-	-	-	-	-	-	-	-	-	-	>50	-	-	>50
	-	-	-	-	-	-	-	-	-	-	-	-	-	-	-	-	-	-	-	-	-	-
**PA0044**	-	-	-	-	-	-	-	-	-	-	-	-	-	>50	-	-	-	-	-	-	-	-
	-	-	-	-	-	-	-	-	-	-	-	-	-	-	-	-	-	-	-	-	-	-
**PA0198**	-	-	-	-	>50	-	-	-	-	-	-	-	-	-	-	-	-	-	-	-	-	-
	-	-	-	-	-	-	-	-	-	-	-	-	-	-	-	-	-	-	-	-	-	-
**PA0411**	-	-	-	-	-	-	-	-	-	-	-	-	-	-	-	-	>50	-	-	-	-	-
	-	-	-	-	-	-	-	-	-	-	-	-	-	-	-	-	-	-	-	-	-	-
**PA0423**	-	>50	-	-	-	-	-	-	-	-	-	-	-	-	-	-	-	-	-	-	-	-
	-	-	-	-	-	-	-	-	-	-	-	-	-	-	-	-	-	-	-	-	-	-
**PA0425**	-	-	-	-	-	-	-	-	-	-	-	-	-	-	-	-	-	>50	-	-	-	-
	-	-	-	-	-	-	-	-	-	-	-	>50	-	-	-	-	-	-	-	-	-	-
**PA0575**	-	-	-	-	-	-	-	-	-	-	-	>50	-	-	-	-	-	-	-	-	-	-
	-	-	-	-	-	-	-	-	-	-	-	-	-	-	-	-	-	-	-	-	-	-
**PA0705**	-	-	-	-	-	-	-	-	>50	-	-	-	-	-	-	-	-	-	-	-	-	-
	-	-	>50	-	>50	-	>50	-	-	-	>50	-	<50	-	>50	-	>50	-	>50	-	>50	-
**PA0732**	-	-	-	-	-	-	-	-	-	-	-	-	-	-	-	>50	-	-	-	-	-	-
	-	-	-	-	-	-	-	-	-	-	-	-	-	-	-	-	-	-	-	-	-	-
**PA0788**	-	-	-	-	-	-	-	-	-	-	-	-	-	>50	-	-	-	>50	-	-	-	-
	-	-	-	-	-	-	-	-	-	-	-	-	-	-	-	-	-	>50	-	-	-	-
**PA0869**	-	>50	-	-	>50	-	-	-	-	>50	-	-	-	-	>50	>50	>50	>50	-	-	-	-
	-	-	>50	-	>50	-	>50	-	>50	-	>50	-	>50	>50	>50	-	>50	-	>50	-	>50	-
**PA0973**	-	-	-	-	-	-	-	-	-	-	-	-	-	-	-	-	-	>50	-	-	-	-
	-	-	-	-	-	-	-	-	-	-	-	>50	-	-	-	-	-	-	-	-	-	-

Partners (prey)	Lytic transglycosylases (bait)																					
Locus tag	MltA		MltB		MltD		MltF		MltF2		MltG		RlpA		Slt		SltB1		SltB2		SltB3	
	Mem	Sol	Mem	Sol	Mem	Sol	Mem	Sol	Mem	Sol	Mem	Sol	Mem	Sol	Mem	Sol	Mem	Sol	Mem	Sol	Mem	Sol
**PA1053**	-	-	-	-	-	-	-	-	-	-	-	-	-	>50	-	-	-	-	-	-	-	-
	-	-	-	-	-	-	-	-	-	-	-	-	-	-	-	-	-	-	-	-	-	-
**PA1091**	-	-	-	-	-	-	-	-	-	-	-	-	-	-	-	-	-	-	-	-	-	>50
	-	-	-	-	-	-	-	-	-	-	-	-	-	-	-	-	-	-	-	>50	-	-
**PA1100**	-	-	-	-	-	-	-	-	-	>50	-	-	-	-	-	-	-	-	-	-	-	-
	-	-	-	-	-	-	-	-	-	-	-	-	-	-	-	-	-	-	-	-	-	-
**PA1171**	-	-	-	-	>50	>50	-	-	-	-	-	-	>50	>50	-	-	-	-	>50	>50	-	-
	-	-	>50	-	>50	-	>50	-	>50	-	>50	-	>50	-	>50	-	>50	-	>50	-	>50	-
**PA1222**	>50	>50	-	-	-	-	-	-	>50	-	-	-	-	-	-	-	-	-	-	-	-	-
	-	>50	>50	-	>50	>50	>50	-	>50	-	>50	-	>50	-	>50	-	>50	-	>50	-	>50	-
**PA1377**	-	-	-	-	-	-	-	-	-	-	-	-	-	-	-	-	-	-	-	-	-	>50
	-	-	-	-	-	-	-	-	-	-	-	-	-	-	-	-	-	-	-	-	-	-
**PA1812**	-	-	-	-	>50	>50	-	-	-	-	>50	>50	>50	>50	-	-	-	-	-	-	-	-
	-	-	-	-	>50	-	-	-	-	-	>50	>50	-	-	-	-	-	-	7	-	-	-
**PA2019**	-	-	-	-	-	-	-	-	-	-	-	-	-	-	-	-	>50	-	-	-	-	-
	-	-	-	-	-	-	-	-	-	-	-	-	-	-	-	-	-	-	-	-	-	-
**PA2057**	-	-	>50	-	-	-	-	-	-	-	-	-	-	-	-	-	-	-	-	-	-	-
	-	-	-	-	-	-	-	-	-	-	-	-	-	-	-	-	-	-	-	-	-	-
**PA2234**	-	-	-	-	-	-	-	-	-	-	-	-	-	-	-	-	-	-	-	-	-	>50
	-	-	-	-	-	-	-	-	-	-	-	-	-	-	-	-	-	-	-	-	-	-
**PA2394**	-	>50	-	-	-	-	-	-	-	>50	-	-	-	-	-	-	-	-	-	-	-	-
	-	-	-	-	-	-	-	-	-	-	-	-	-	-	-	-	-	-	-	-	-	-
**PA2400**	-	-	-	-	-	-	-	-	-	>50	-	-	-	-	-	-	-	-	-	-	-	-
	-	-	-	-	-	-	-	-	-	-	-	-	-	-	-	-	-	-	-	-	-	-

Partners (prey)	Lytic transglycosylases (bait)																					
**Locus tag**	**MltA**		**MltB**		**MltD**		**MltF**		**MltF2**		**MltG**		**RlpA**		**Slt**		**SltB1**		**SltB2**		**SltB3**	
	Mem	Sol	Mem	Sol	Mem	Sol	Mem	Sol	Mem	Sol	Mem	Sol	Mem	Sol	Mem	Sol	Mem	Sol	Mem	Sol	Mem	Sol
**PA2424**	-	-	-	-	-	-	-	-	-	-	-	-	-	>50	-	-	-	-	-	-	-	-
	-	-	-	-	-	-	-	-	-	-	-	-	-	-	-	-	-	-	-	-	-	-
**PA2493**	-	-	-	-	-	-	>50	>50	-	-	-	-	-	-	-	-	-	>50	-	-	-	-
	-	-	-	-	-	-	-	-	-	-	-	-	-	-	-	-	-	-	-	-	-	-
**PA2495**	-	-	-	-	-	-	-	-	-	-	-	-	-	-	-	-	-	>50	-	-	-	-
	-	-	-	-	-	-	-	-	-	-	-	>50	-	-	-	-	-	-	-	-	-	-
**PA2530**	-	-	-	>50	-	>50	-	-	-	>50	-	-	-	-	-	-	-	-	-	-	-	>50
	-	-	-	-	-	>50	-	-	-	-	-	-	-	-	-	-	-	-	-	-	-	-
**PA2656**	-	-	-	-	-	-	-	-	-	-	-	>50	-	-	-	-	-	-	-	-	-	-
	-	-	-	-	-	-	-	-	-	-	-	-	-	-	-	-	-	-	-	-	-	-
**PA2684**	-	-	-	-	-	-	-	-	-	-	-	-	-	-	-	>50	-	-	-	-	-	-
	-	-	-	>50	-	-	-	-	-	-	-	-	-	-	-	-	-	-	-	-	-	>50
**PA2702**	-	-	-	-	-	-	-	-	-	-	-	-	-	-	-	>50	-	-	-	-	-	-
	-	-	-	-	-	-	-	-	-	-	-	-	-	-	-	-	-	-	-	-	-	-
**PA2755**	-	-	-	-	-	2	-	-	>50	-	-	-	-	-	-	-	-	-	-	-	-	-
	-	-	-	-	-	-	-	-	-	-	-	-	-	-	-	-	-	-	-	-	-	-
**PA2854**	-	-	-	-	-	-	-	-	>50	-	-	-	-	-	-	-	-	-	-	-	-	-
	-	-	-	-	-	-	-	-	-	>50	-	-	-	>50	-	-	-	-	-	-	-	-
**PA2865**	-	-	-	>50	-	-	-	-	-	>50	-	-	-	-	-	-	-	-	-	>50	-	-
	-	-	>50	-	>50	-	>50	-	>50	-	>50	-	>50	-	>50	-	>50	-	>50	-	-	-
**PA2960**	-	-	-	-	-	-	-	-	-	>50	-	-	-	-	-	-	-	-	-	-	-	-
	-	-	-	-	-	-	-	-	-	-	-	-	-	-	-	-	-	-	-	-	-	-
**PA3020**	-	-	-	-	-	-	-	-	-	-	>50	>50	-	-	>50	>50	-	-	-	-	-	-
	-	10	-	-	5	-	-	-	>50	-	-	-	-	-	>50	>50	-	-	8	-	3	-

Partners (prey)	Lytic transglycosylases (bait)																					
Locus tag	MltA		MltB		MltD		MltF		MltF2		MltG		RlpA		Slt		SltB1		SltB2		SltB3	
	Mem	Sol	Mem	Sol	Mem	Sol	Mem	Sol	Mem	Sol	Mem	Sol	Mem	Sol	Mem	Sol	Mem	Sol	Mem	Sol	Mem	Sol
**PA3047**	-	-	-	-	7	>50	-	-	-	-	-	-	-	-	-	-	-	-	-	-	3	>50
	-	-	>50	-	>50	-	>50	-	>50	-	>50	-	>50	-	>50	-	>50	-	>50	-	>50	-
**PA3060**	-	-	-	-	>50	-	-	-	-	-	-	-	-	-	-	-	-	-	-	-	-	-
	-	-	-	-	-	-	-	-	-	-	-	-	-	-	-	-	-	-	-	-	-	-
**PA3160**	-	-	-	-	-	-	-	-	-	-	-	-	-	>50	-	-	>50	-	-	-	-	-
	-	-	>50	-	>50	-	>50	-	>50	-	>50	-	>50	-	>50	-	>50	-	>50	-	>50	-
**PA3205**	-	>50	-	-	-	-	-	-	-	8	-	-	-	-	-	-	-	-	-	-	-	4
	-	-	-	-	-	-	-	-	-	-	-	-	-	-	-	-	-	-	-	-	-	-
**PA3257**	-	-	-	-	-	-	-	-	-	-	-	>50	-	-	>50	-	-	-	-	-	-	>50
	-	-	>50	-	>50	>50	>50	-	>50	>50	>50	-	>50	>50	>50	-	>50	-	>50	>50	>50	>50
**PA3535**	-	-	-	-	-	-	-	-	-	-	>50	-	-	-	-	-	-	-	-	-	-	-
	-	-	-	-	-	-	-	-	-	-	-	-	-	-	-	-	-	-	-	-	-	-
**PA3553**	-	-	-	-	-	-	>50	>50	-	-	-	-	-	-	-	-	>50	-	-	-	-	-
	-	-	-	-	-	-	-	-	-	-	-	-	-	-	-	-	4	-	-	-	-	-
**PA3764**	-	-	-	-	-	-	>50	>50	-	-	-	-	-	-	-	4	-	-	-	-	-	-
	-	>50	>50	>50	>50	-	>50	-	>50	-	>50	-	>50	-	>50	-	>50	-	>50	-	>50	-
**PA3805**	-	-	-	-	-	-	-	-	-	-	-	-	-	-	-	-	>50	>50	-	-	-	>50
	-	-	-	-	-	-	-	-	-	-	-	-	-	-	-	-	-	>50	-	-	-	-
**PA3866**	-	-	-	-	-	>50	-	-	-	-	-	-	-	-	-	-	-	-	-	-	-	-
	-	-		-	-	-	-	-	-	-	-	-	-	-	-	-	-	-	-	-	-	-
**PA3953**	-	-	-	-	-	-	-	-	-	>50	-	-	-	-	-	-	-	-	-	-	-	-
	-	-	-	-	-	-	-	-	-	-	-	-	-	-	-	-	-	-	-	-	-	-
**PA3988**	-	-	-	-	-	-	-	-	-	-	-	-	-	-	-	-	>50	>50	-	-	-	-
	-	-	>50	-	>50	-	>50	-	>50	-	>50	-	>50	-	>50	-	>50	-	>50	-	>50	-

Partners (prey)	Lytic transglycosylases (bait)																					
Locus tag	MltA		MltB		MltD		MltF		MltF2		MltG		RlpA		Slt		SltB1		SltB2		SltB3	
	Mem	Sol	Mem	Sol	Mem	Sol	Mem	Sol	Mem	Sol	Mem	Sol	Mem	Sol	Mem	Sol	Mem	Sol	Mem	Sol	Mem	Sol
PA3992	-	-	-	-	>50	>50	-	-	-	-	-	-	>50	>50	-	-	-	-	>50	>50	>50	>50
	-	-	>50	3	>50	-	>50	-	>50	>50	>50	-	>50	-	>50	3	>50	-	>50	>50	>50	>50
PA3999	-	-	-	-	-	-	>50	>50	-	-	-	-	-	-	>50	-	>50	>50	-	-	-	-
	-	-	-	-	-	-	3	3	-	-	-	>50	-	-	5	-	-	-	-	-	-	-
PA4000	>50	>50	-	-	-	>50	-	-	>50	>50	-	-	>50	>50	-	-	-	-	-	-	-	-
	-	-	>50	-	>50	-	>50	-	>50	-	>50	-	>50	-	>50	-	>50	-	>50	-	>50	-
PA4001	-	-	-	-	-	-	-	-	-	-	-	-	-	-	-	-	>50	>50	-	-	-	-
	-	-	>50	-	>50	-	>50	>50	>50	-	>50	>50	>50	-	>50	-	>50	38	-	-	-	-
PA4035	-	-	-	-	-	-	-	-	-	>50	-	-	-	-	-	-	-	-	-	-	-	-
	-	-	-	-	-	-	-	-	-	-	-	-	-	-	-	-	-	-	-	-	-	-
PA4063	-	-	-	-	-	>50	-	-	-	>50	-	-	-	>50	-	-	-	-	-	>50	-	-
	-	-	-	-	-	-	-	-	-	-	-	-	-	-	-	-	-	-	-	-	-	-
PA4208	-	-	-	>50	-	-	-	-	-	-	-	-	-	-	-	-	-	-	-	-	-	-
	-	-	-	-	-	-	-	-	-	-	-	-	-	-	-	-	-	-	-	-	-	-
PA4310	-	-	-	-	-	-	-	-	-	-	-	-	-	-	-	-	>50	-	-	-	-	-
	-	-	-	-	-	-	-	-	-	-	-	-	-	-	-	-	-	-	-	-	-	-
PA4370	-	-	-	-	-	>50	-	-	-	-	-	-	-	-	-	-	-	-	-	-	-	-
	-	-	-	-	-	-	-	-	-	-	-	-	-	-	-	-	-	-	-	-	-	-
PA4444	-	-	>50	>50	>50	-	-	-	-	-	-	-	>50	>50	-	-	-	-	>50	>50	>50	>50
	-	-	>50	>50	>50	>50	>50	>50	>50	-	>50	-	>50	>50	>50	-	>50	-	>50	-	>50	-
PA4460	-	-	-	-	>50	>50	-	-	-	-	-	-	-	-	-	-	-	-	-	-	-	-
	-	-	-	-	-	-	-	-	-	-	-	-	-	-	-	-	-	-	-	-	-	-
PA4489	-	-	-	>50	-	-	-	-	-	-	-	-	-	-	-	-	-	-	-	-	-	-
	-	-	-	-	-	-	-	-	-	-	4	-	-	-	-	-	-	-	3	-	-	-
PA4525	-	-	-	-	-	-	-	-	-	-	-	-	-	>50	-	-	-	-	-	-	-	-
	-	-	-	-	-	-	-	-	-	-	-	>50	-	-	-	-	-	-	-	-	-	-
PA4545	-	-	-	-	-	-	-	-	-	-	-	-	-	-	-	-	>50	-	-	-	-	-
	-	-	-	-	-	-	-	-	-	-	-	-	-	-	-	-	-	-	-	-	-	-

Partners (prey)	Lytic transglycosylases (bait)																					
Locus tag	MltA		MltB		MltD		MltF		MltF2		MltG		RlpA		Slt		SltB1		SltB2		SltB3	
	Mem	Sol	Mem	Sol	Mem	Sol	Mem	Sol	Mem	Sol	Mem	Sol	Mem	Sol	Mem	Sol	Mem	Sol	Mem	Sol	Mem	Sol
**PA4592**	-	-	-	>50	-	-	-	-	-	-	-	-	-	-	-	-	-	-	-	-	-	-
	-	-	-	-	-	-	-	-	-	-	-	3	-	-	-	-	-	-	-	-	-	-
**PA4632**	-	-	>50	-	-	-	-	-	-	7	-	2	-	-	-	-	-	-	-	-	-	-
	-	-	-	-	-	-	-	-	-	-	-	3	-	-	-	-	-	-	-	-	-	-
**PA4700**	-	-	-	-	-	-	-	-	-	-	-	-	-	-	>50	-	-	-	-	-	-	-
	-	-	>50	-	>50	-	>50	-	>50	-	>50	-	>50	-	>50	-	>50	-	>50	-	>50	-
**PA4777**	-	-	-	-	-	-	-	-	-	-	>50	-	-	-	-	-	-	-	-	-	-	-
	-	-	>50	-	>50	-	>50	-	>50	-	>50	-	>50	-	>50	-	>50	-	>50	-	>50	-
**PA5037**	-	-	-	-	-	-	-	-	-	>50	-	-	-	-	-	>50	-	-	-	-	-	-
	-	-	-	-	-	-	-	-	-	-	-	-	-	-	-	-	-	-	-	-	-	-
**PA5042**	-	-	-	-	-	-	>50	>50	-	-	-	-	-	-	>50	-	>50	-	-	-	-	-
	-	-	>50	-	>50	-	>50	-	>50	-	>50	-	>50	-	>50	-	>50	-	>50	-	>50	-
**PA5043**	-	-	-	-	-	-	>50	>50	-	-	-	-	-	-	>50	-	-	-	-	-	-	-
	-	-	-	-	-	-	-	-	-	-	-	-	-	-	-	-	-	-	-	-	-	-
**PA5045**	-	-	-	-	-	-	-	-	-	-	-	-	-	-	-	-	>50	-	-	-	-	-
	-	-	>50	-	>50	-	>50	-	>50	-	>50	-	>50	-	>50	-	>50	-	>50	-	>50	-
**PA5117**	-	>50	-	-	-	-	-	-	-	-	-	-	-	>50	-	-	-	-	-	-	-	-
	-	-	>50	-	>50	-	>50	-	>50	-	>50	-	>50	-	>50	-	>50	-	>50	-	>50	-

The fold enrichment of each putative partner is calculated as the ratio of the spectral count intensity of each sample compared to the control (without LT). Only proteins with 2-fold or more in enrichment, which localize to the periplasm, were considered. Integral membrane proteins were excluded from further analysis. Pulldown assays with all 11 His-tagged LTs (bait) and with the membrane (left column) or the soluble (right column) fractions of *P. aeruginosa* PAO1 (prey) were performed in the presence (white rows) or the absence (gray rows) of the cross-linker bis(sulfosuccinimidyl)suberate (BS^3, Thermo Scientific, Fig. 5). The binding of each LT to itself does not imply oligomerization of the protein. It is caused by the presence of the bait, as mass spectrometry cannot distinguish between the native protein from the bacterium and the heterologously expressed recombinant His-LT. The dash lines indicate that the protein was not enriched/identified in that specific fraction. The proteins are listed in the locus tag order.

Supplementary Table 3. List of the putative partners for all 11 LTs with attributed name, functions, and localization.

Locus tag	Name	Localization	Function
**PA0041**	Hemoagglutinin	Outer membrane/ Extracellular	Filamentous hemoagglutinin
**PA0044**	ExoT	Extracellular	Effector toxin of the type 3 secretion system
**PA0198**	ExbB	Cytoplasmic membrane	MotA/TolQ/ExbB proton channel
**PA0411**	PilJ	Outer membrane	Type IV pili methyl-accepting chemotaxis transducer
**PA0423**	PasP	Unknown	Lipid/polyisoprenoid-binding YceI-like domain
**PA0425**	MexA	Cytoplasmic membrane	Resistance-Nodulation-Cell Division (RND) multidrug efflux membrane fusion protein
**PA0575**	RmcA	Cytoplasmic membrane	Solute-binding protein
**PA0705**	MigA	Cytoplasmic membrane	LPS biosynthetic process/Biofilm formation. Glycosyl transferase activity.
**PA0732**	Hypothetical Protein	Unknown	Cysteine-type peptidase activity Peptidoglycan L,D-transpeptidase
**PA0788**	Hypothetical Protein	Outer membrane	Glycosyl transferase PBP transglycosylase domain
**PA0869**	PBP7	Periplasm	D-Ala-D-Ala- carboxypeptidase
**PA0973**	OprL	Outer membrane	Peptidoglycan-associated lipoprotein with OmpA domain
**PA1053**	SlyB	Outer membrane	Glycine zipper 2TM domain
**PA1091**	FgtA	Cytoplasmic membrane	Glycosyl transferase
**PA1100**	FliE	Periplasm	Flagellar hook-basal body complex protein
**PA1171**	SltB2	Cytoplasmic membrane	Lytic transglycosylase
**PA1222**	MltA	Outer membrane	Lytic transglycosylase
**PA1377**	Pitax	Unknown	N-acetyltransferase activity
**PA1812**	MltD	Cytoplasmic membrane	Lytic transglycosylase
**PA2019**	MexX	Cytoplasmic membrane	Resistance-Nodulation-Cell Division (RND) multidrug efflux membrane fusion protein
**PA2057**	SppR	Outer membrane	TonB-dependent receptor
**PA2234**	PslD	Outer membrane	Polysaccharide biosynthesis in biofilm formation
**PA2394**	PvdN	Outer membrane	Pyoverdine biosynthesis
**PA2400**	PvdJ	Unknown	Pyoverdine biosynthesis process / AMP-dependent synthetase
**PA2424**	PvdL	Unknown	Pyoverdine biosynthesis process /AMP-dependent synthetase
**PA2493**	MexE	Cytoplasmic membrane	Multidrug efflux pump

Locus tag	Name	Localization	Function
**PA2495**	OprN	Outer membrane	Multidrug efflux pump
**PA2530**	Hypothetical Protein	Unknown	Metalloprotease activity
**PA2656**	CarS	Cytoplasmic membrane	Signal transduction histidine kinase related-protein
**PA2684**	Tse5	Outer membrane	Type VI secretion system[e] RHS protein signature
**PA2702**	Tse2	Unknown	ADP-ribosyltransferase toxin
**PA2755**	Eco	Periplasm	Serine endopeptidase-inhibitor activity
**PA2854**	Hypothetical Protein	Unknown	Peptidoglycan L,D-transpeptidase activity
**PA2865**	MltF2	Outer membrane	Lytic transglycosylase
**PA2960**	PilZ	Unknown	Pilus formation
**PA3020**	Slt	Periplasm	Lytic transglycosylase
**PA3047**	PBP4	Periplasm	D-Ala-D-Ala carboxypeptidase
**PA3060**	PelE	Cytoplasmic membrane	Biofilm formation and extracellular polysaccharide biosynthesis process
**PA3160**	Wzz	Cytoplasmic membrane	Polysaccharide chain length determinant
**PA3205**	Hypothetical Protein	Periplasm	LTXXQ motif
**PA3257**	AlgO	Outer membrane	Signal transduction
**PA3535**	Hypothetical Protein	Outer membrane	Serine protease
**PA3553**	ArnC	Cytoplasmic membrane	Lipid A biosynthesis
**PA3764**	MltF	Outer membrane	Lytic transglycosylase
**PA3805**	PilF	Outer membrane	Type 4 fimbriae biogenesis
**PA3866**	PiocynS4	Extracellular	Pathogenesis/Response to stimulus/receptor binding
**PA3953**	Hypothetical Protein	Periplasm	Metabolic process?
**PA3988**	LptE	Outer membrane	LPS assembly
**PA3992**	SltB3	Cytoplasmic membrane	Lytic transglycosylase
**PA3999**	PBP5	Cytoplasmic membrane	D-Ala-D-Ala carboxypeptidase
**PA4000**	RlpA	Cytoplasmic membrane	Lytic transglycosylase
**PA4001**	SltB1	Cytoplasmic membrane	Lytic transglycosylase
**PA4035**	Hypothetical Protein	Periplasm	Peptidoglycan biosynthesis
**PA4063**	Hypothetical Protein	Periplasm	Unknown function

Locus tag	Name	Localization	Function
**PA4208**	OpmD	Outer membrane	Efflux pump with MexGHI
**PA4310**	PctB	Cytoplasmic membrane	Chemotaxis transducer
**PA4370**	IcmP	Outer membrane	Cellular response to iron
**PA4444**	MltB	Cytoplasmic membrane	Lytic transglycosylase
**PA4460**	LptA/LptH?	Periplasm	Lipopolysaccharide export
**PA4489**	MagD	Cytoplasmic Membrane/Periplasm	Endopeptidase inhibitor activity
**PA4525**	PilA	Periplasm/ Extracellular	Cell adhesion/biofilm formation
**PA4545**	ComL	Outer membrane	Cell envelope organization
**PA4592**	OM precursor	Outer membrane	Outer membrane efflux protein
**PA4632**	Hypothetical Protein	Periplasm	Metalloendopeptidase activity
**PA4700**	PBP1b	Cytoplasmic membrane	Transpeptidase
**PA4777**	PmrB	Cytoplasmic membrane	Phosphorelay signal transduction system
**PA5037**	Hypothetical Protein	Cytoplasmic membrane	PG binding (SPOR domain)
**PA5042**	PilO	Cytoplasmic membrane	Type IV fimbriae biosynthesis
**PA5043**	PilN	Cytoplasmic membrane	Type IV fimbriae biosynthesis
**PA5045**	PBP1a	Cytoplasmic membrane	Transpeptidase
**PA5117**	TypA	Cytoplasmic membrane	Swarming motility/biofilm formation

The name, the function, and the localization of the proteins shown in Table 2 are listed. All localization entries, regardless of classification, reside in the periplasmic milieu. Periplasm denotes protein resides in the milieu and is soluble. Outer membrane denotes protein that is bound to the inner leaflet of the outer membrane. Inner membrane denotes protein that is bound to outer leaflet of the inner membrane. Localization and function of each protein, unless previously published, were found in the following prediction sites: PSORT2b, EMBL-EBI, Pfam, CATH-Gene3D, PRINTS, Hama.

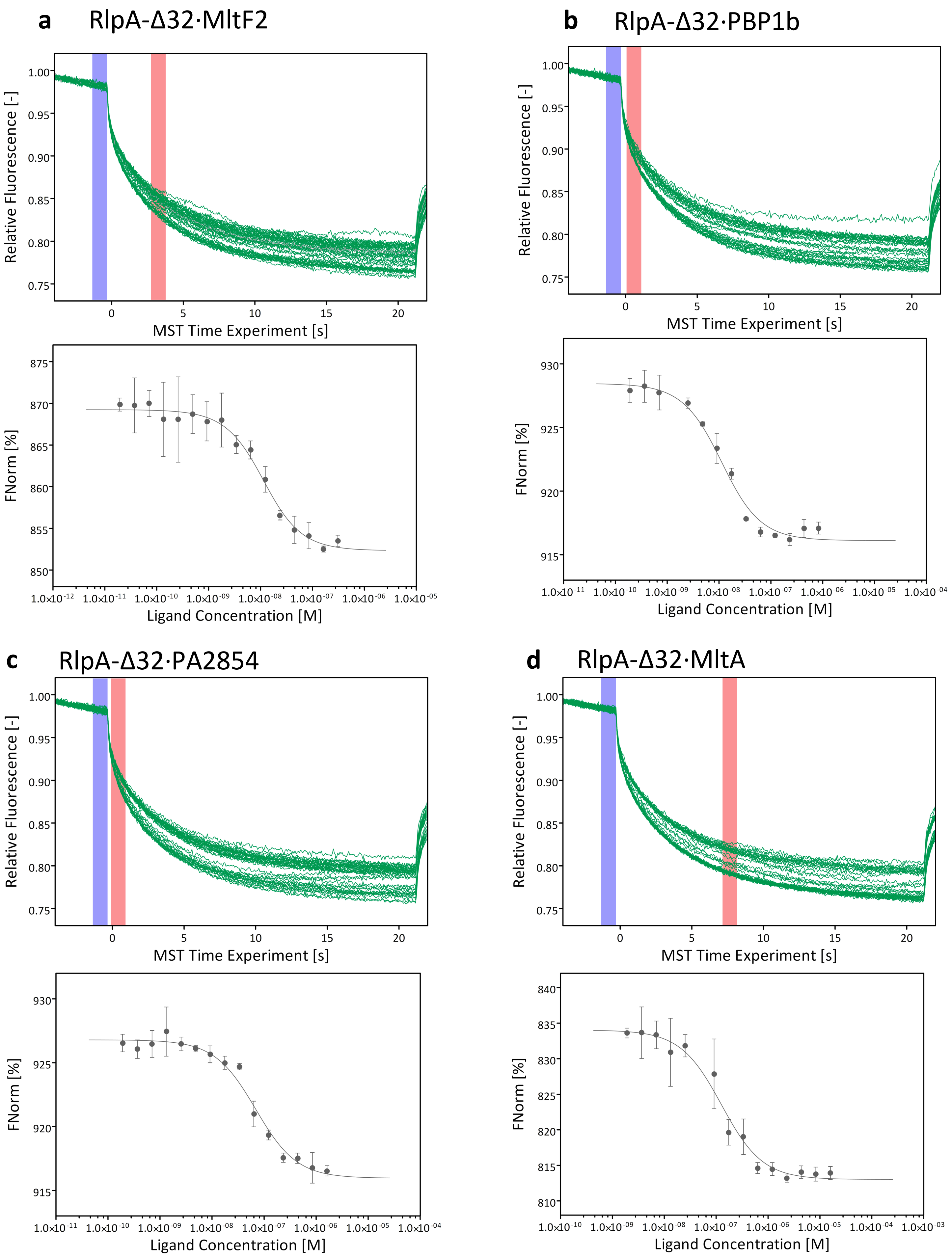
a
RlpA-Δ32·MltF2
b
RlpA-Δ32·PBP1b
c
RlpA-Δ32·PA2854
d
RlpA-Δ32·MltA
Relative Fluorescence [-]
MST Time Experiment [s]
FNorm [%]
Ligand Concentration [M]

e RlpA-Δ32·LptE

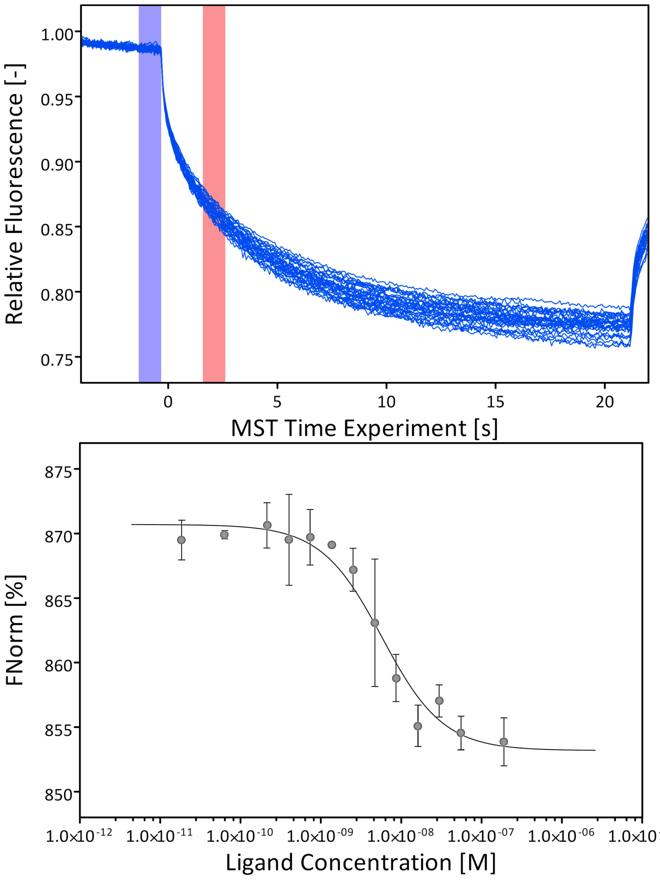

f RlpA-Δ32·SltB3

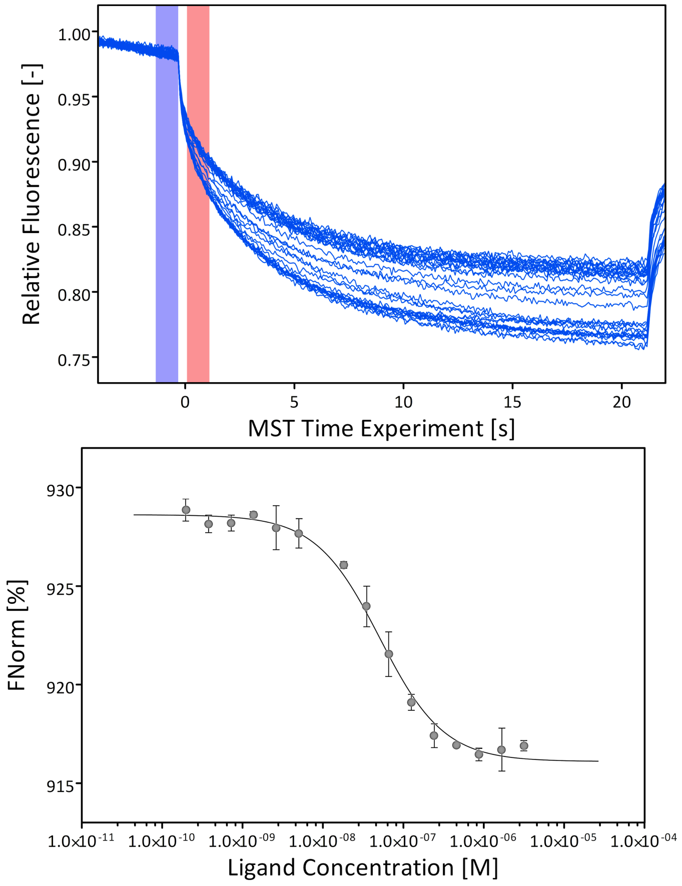

g RlpA-Δ32·PBP1a

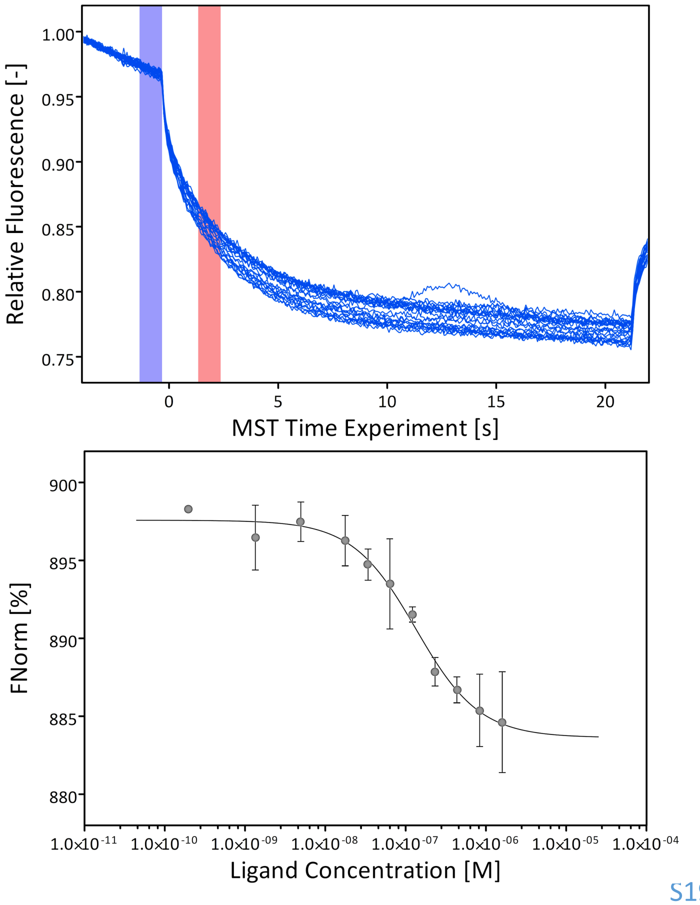

h RlpA-Δ32·MltB

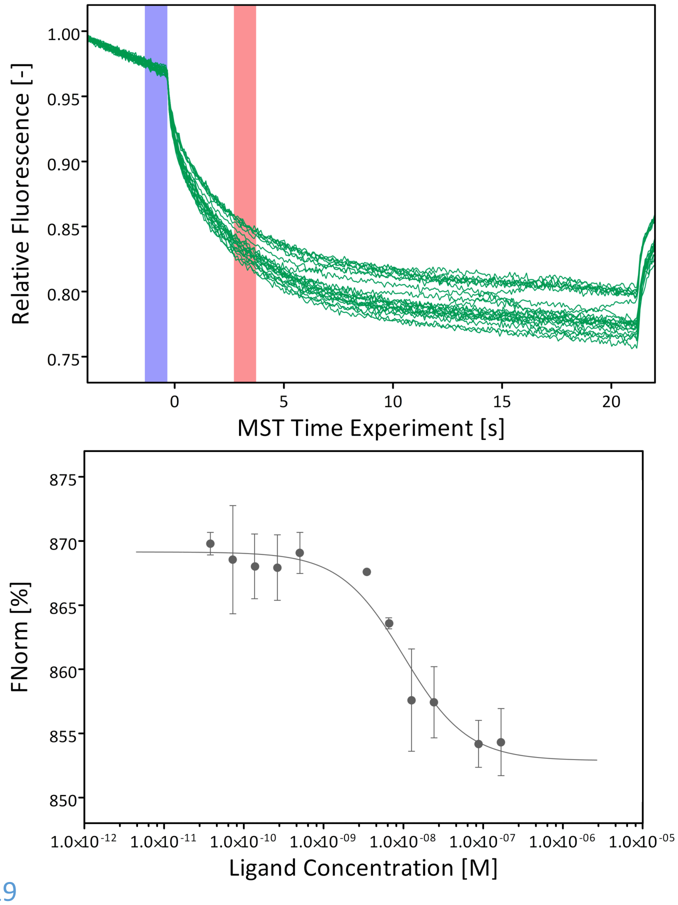

i RlpA-Δ32·MltF

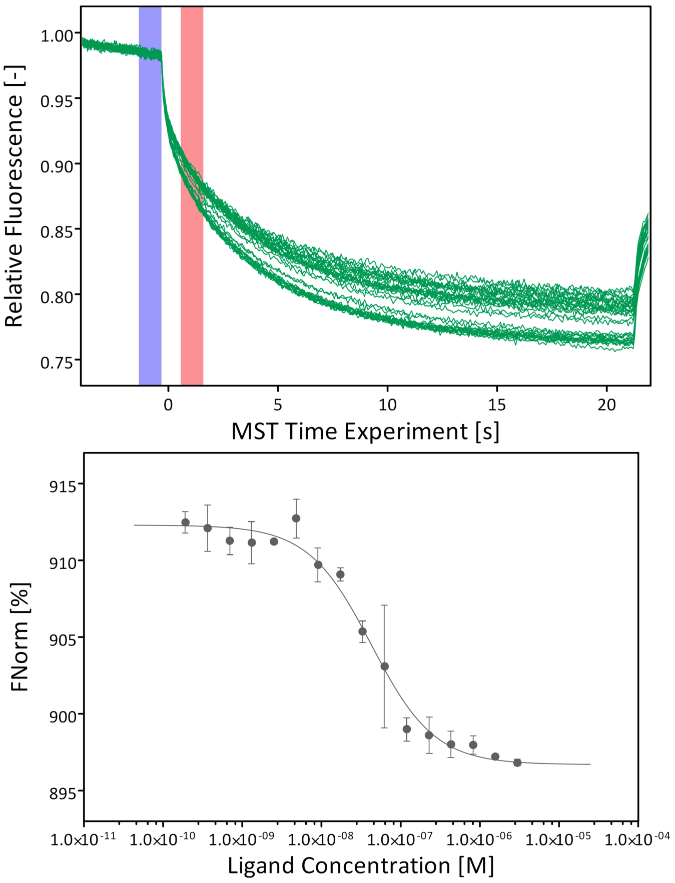

j RlpA-Δ32·SltB2

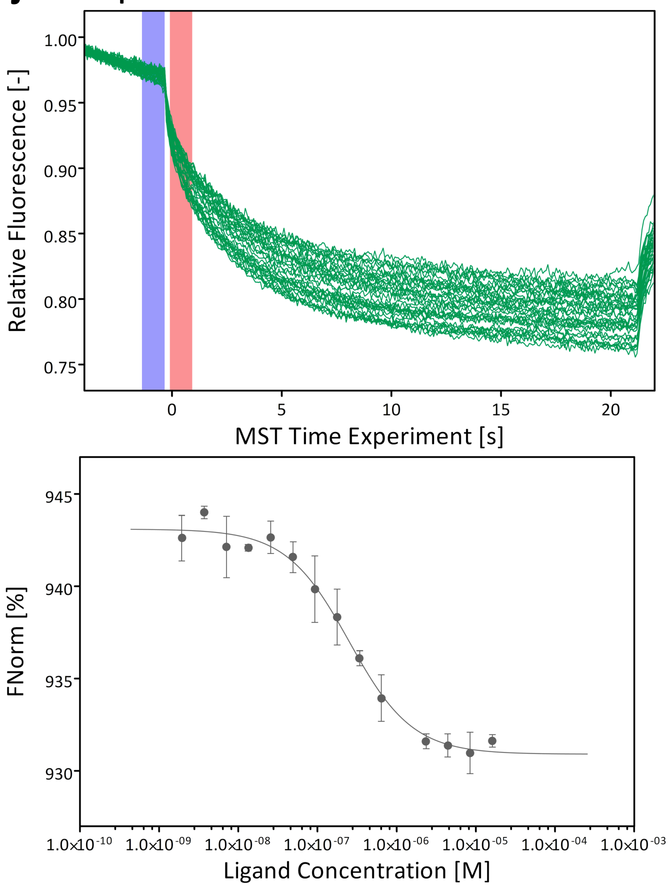

k RlpA-Δ32·SltB1

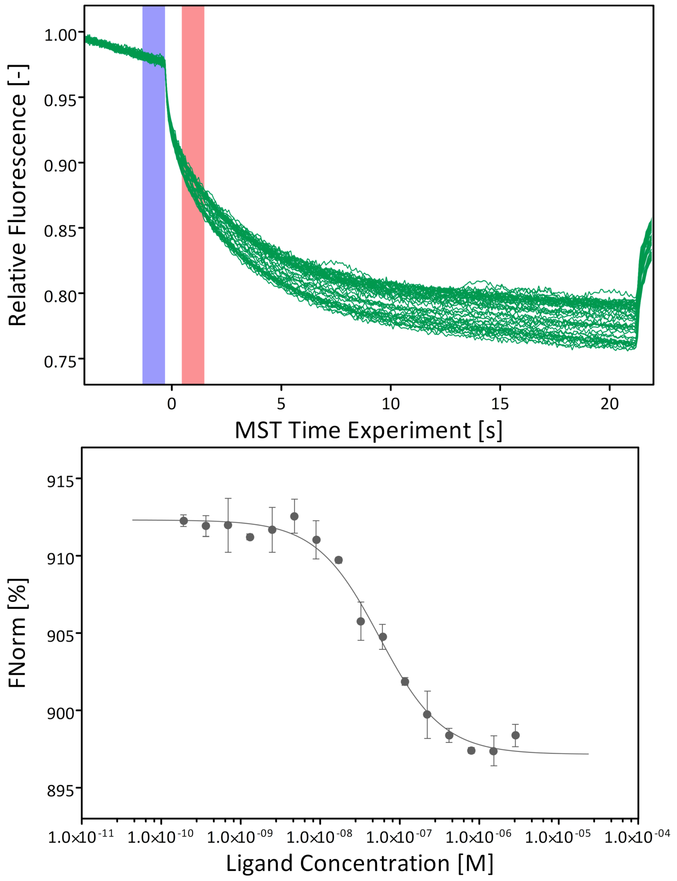

l RlpA-Δ32·PA4063

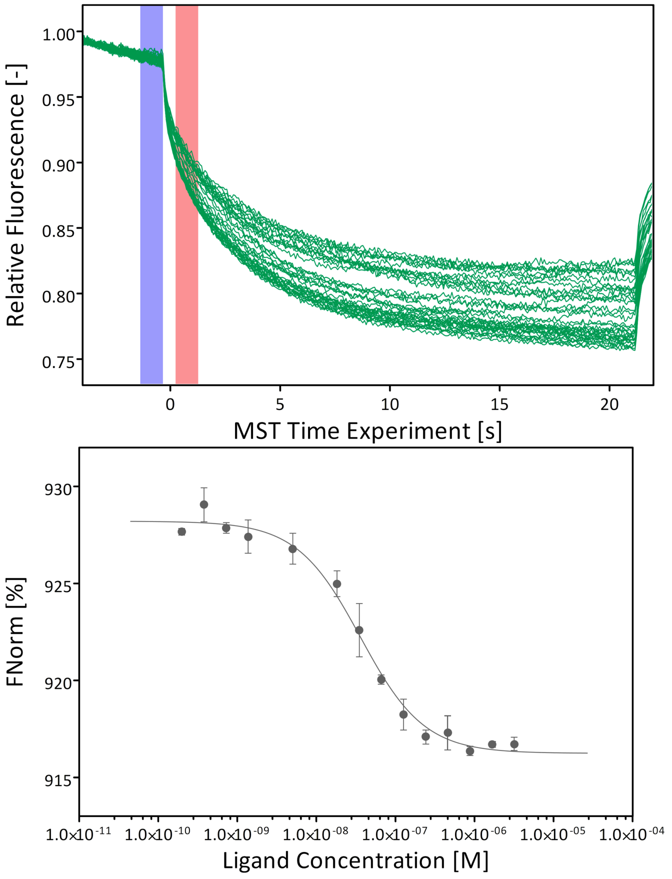

m RlpA-Δ32·MltD

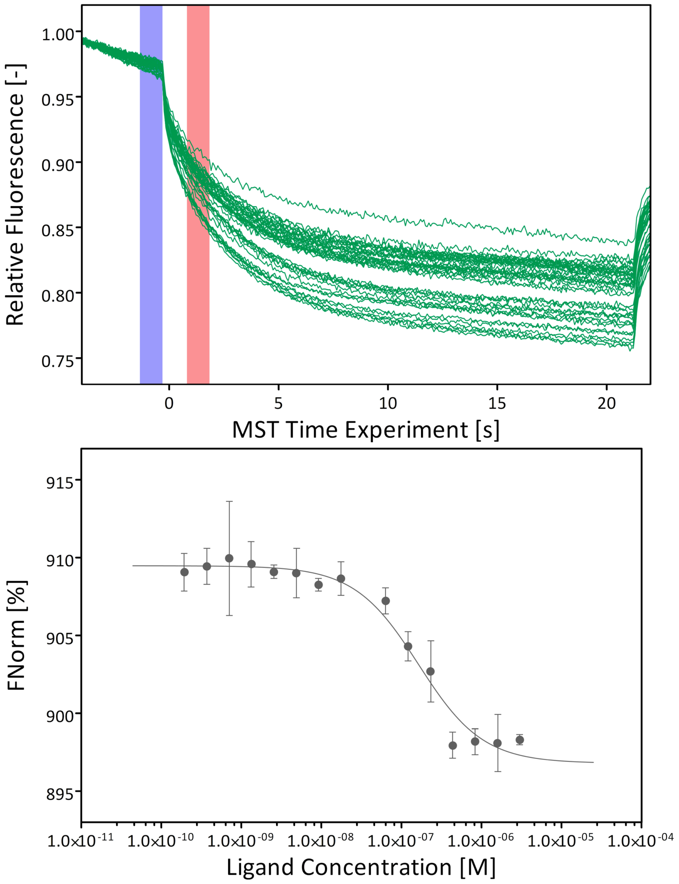

n RlpA-Δ32·PilA

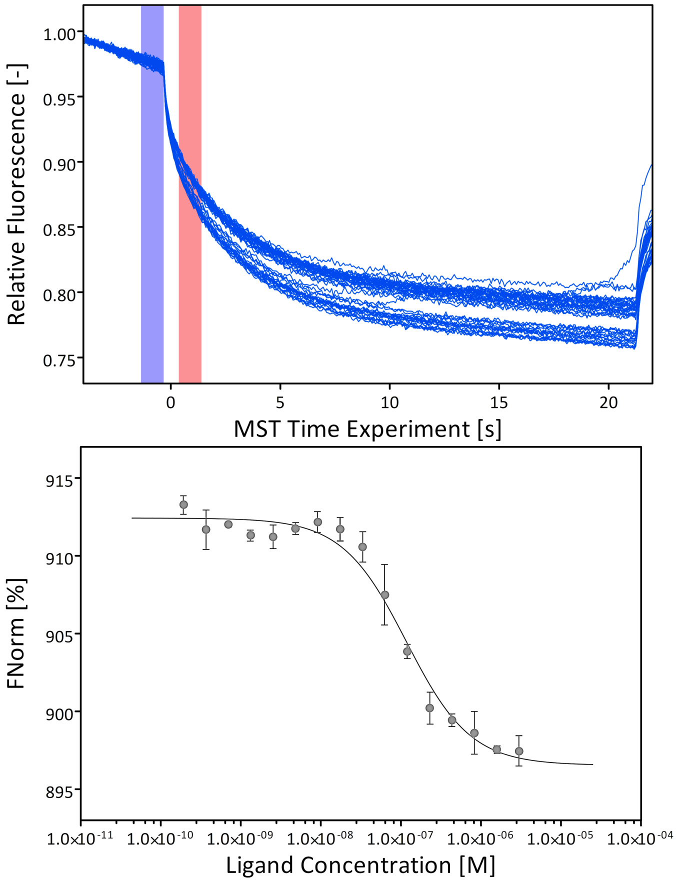

o RlpA-Δ32·PilO

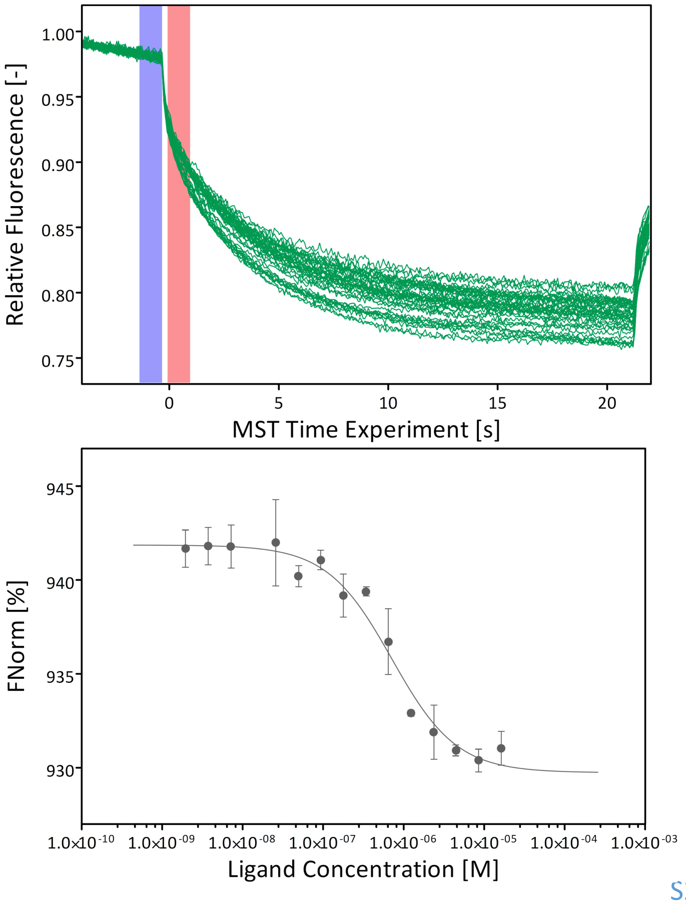

p RlpA-Δ32·PBP7

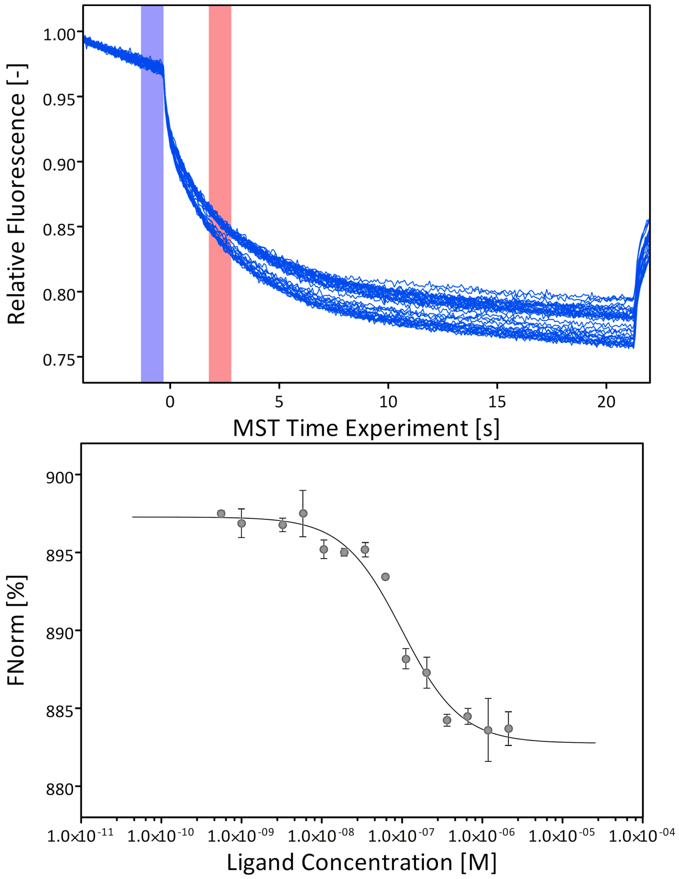

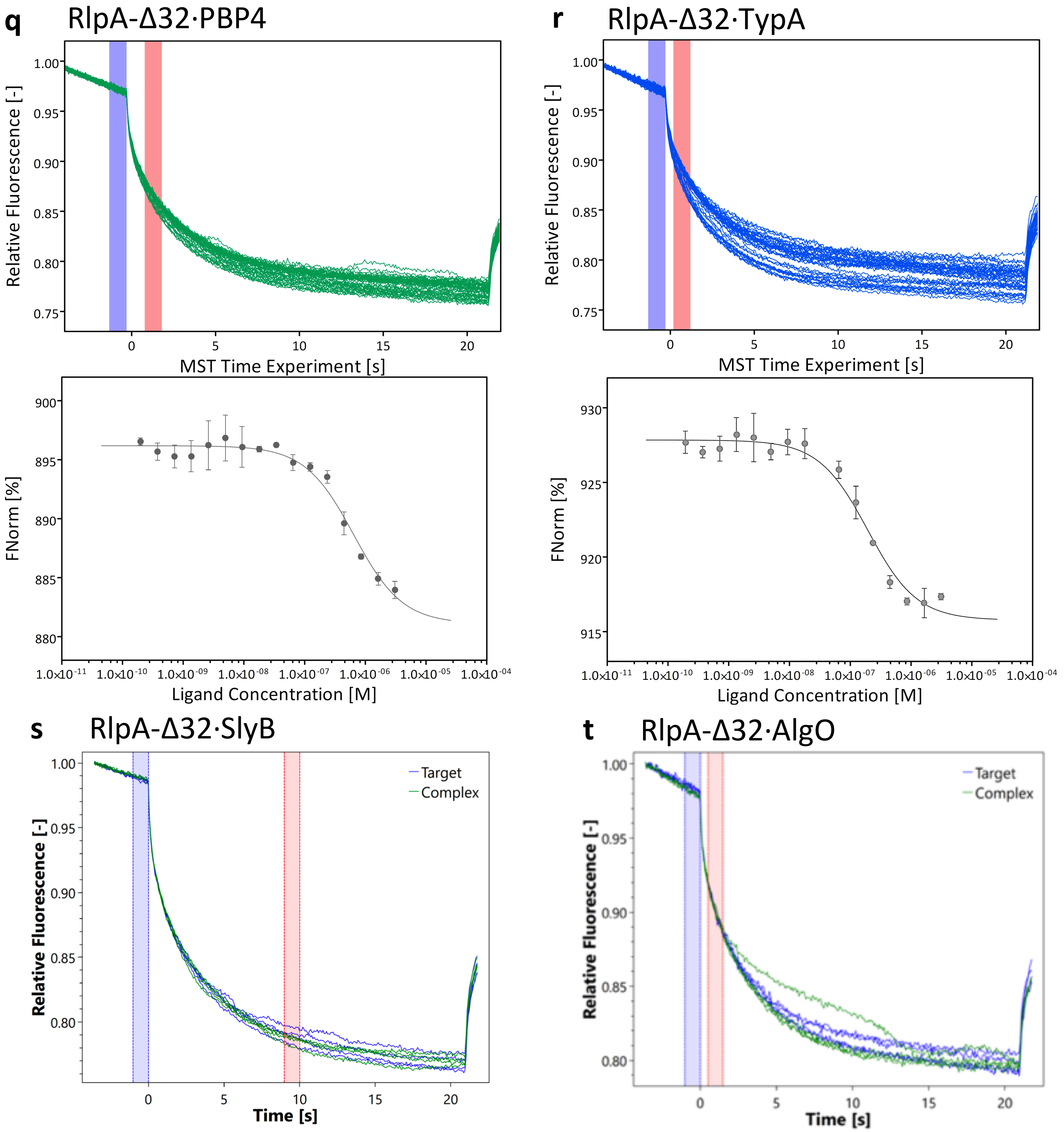

Supplementary Fig. 3. MST traces (top) and dose-response curves (bottom) of tested binary RlpA-Δ32 combinations. **a** RlpA-Δ32 and MltF2. **b** RlpA-Δ32 and PBP1b. **c** RlpA-Δ32 and PA2854. **d** RlpA-Δ32 and MltA. **e** RlpA-Δ32 and LptE. **f** RlpA-Δ32 and SltB3. **g** RlpA-Δ32 and PBP1a. **h** RlpA-Δ32 and MltB. **i** RlpA-Δ32 and MltF. **j** RlpA-Δ32 and SltB2. **k** RlpA-Δ32 and SltB1. **l** RlpA-Δ32 and PA4063. **m** RlpA-Δ32 and MltD. **n** RlpA-Δ32 and PilA. **o** RlpA-Δ32 and PilO. **p** RlpA-Δ32 and PBP7. **q** RlpA-Δ32 and PBP4. **r** RlpA-Δ32 and TypA. **s** RlpA-Δ32 and SlyB; no binding interaction determined within this binary combination. Only MST traces are shown since dose-response curve is not available for this analysis. **t** RlpA-Δ32 and AlgO; no binding interaction determined within this binary combination. Only MST traces are shown since dose-response curve is not available for this analysis. View Table 1 (left column) for K_D parameters of the tested RlpA-Δ32 binary combinations through MST. Data are presented as means ± S.D. from triplicate experiments.

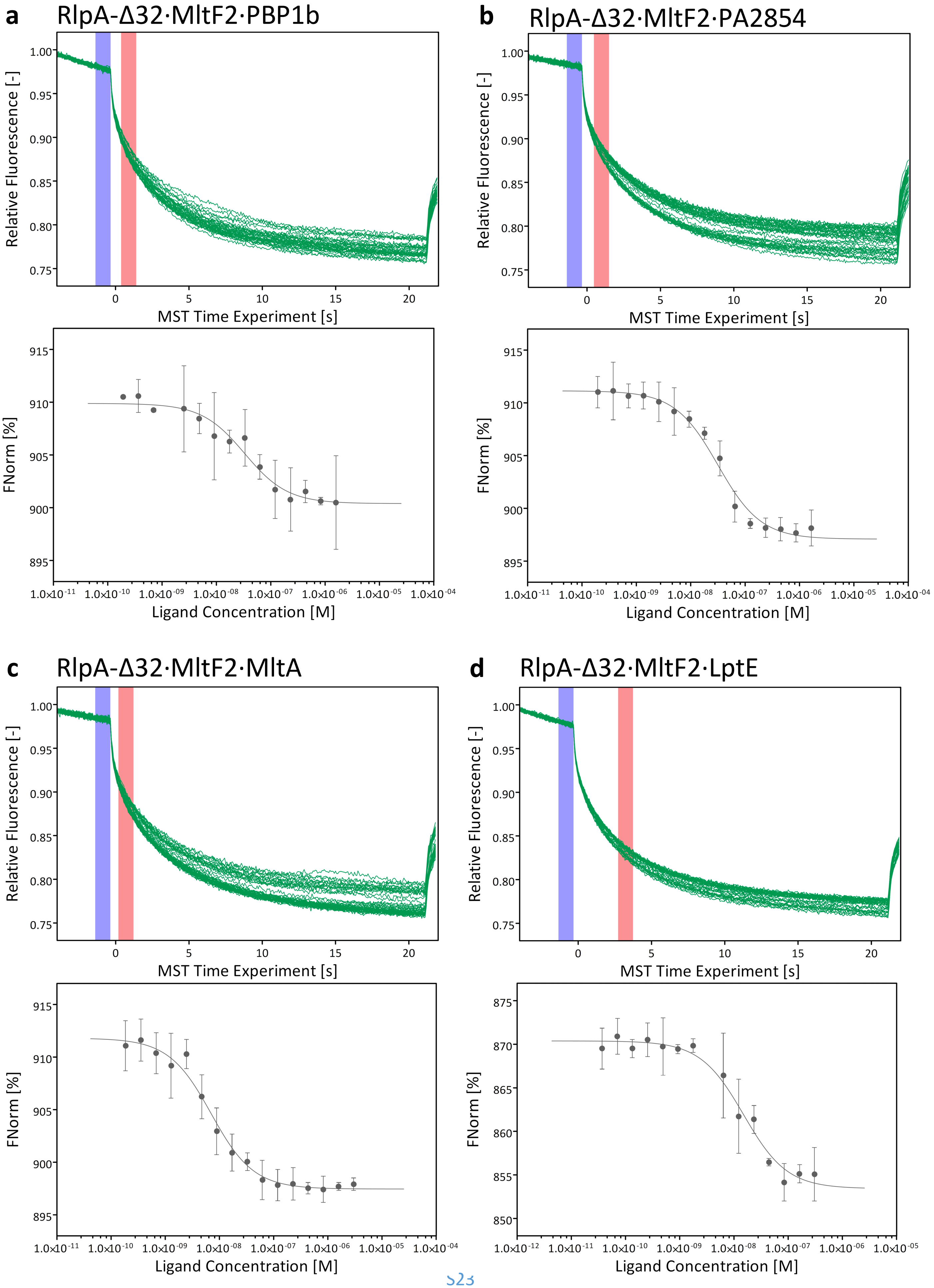
a
RlpA-Δ32·MltF2·PBP1b
b
RlpA-Δ32·MltF2·PA2854
c
RlpA-Δ32·MltF2·MltA
d
RlpA-Δ32·MltF2·LptE
Relative Fluorescence [-]
MST Time Experiment [s]
FNorm [%]
Ligand Concentration [M]

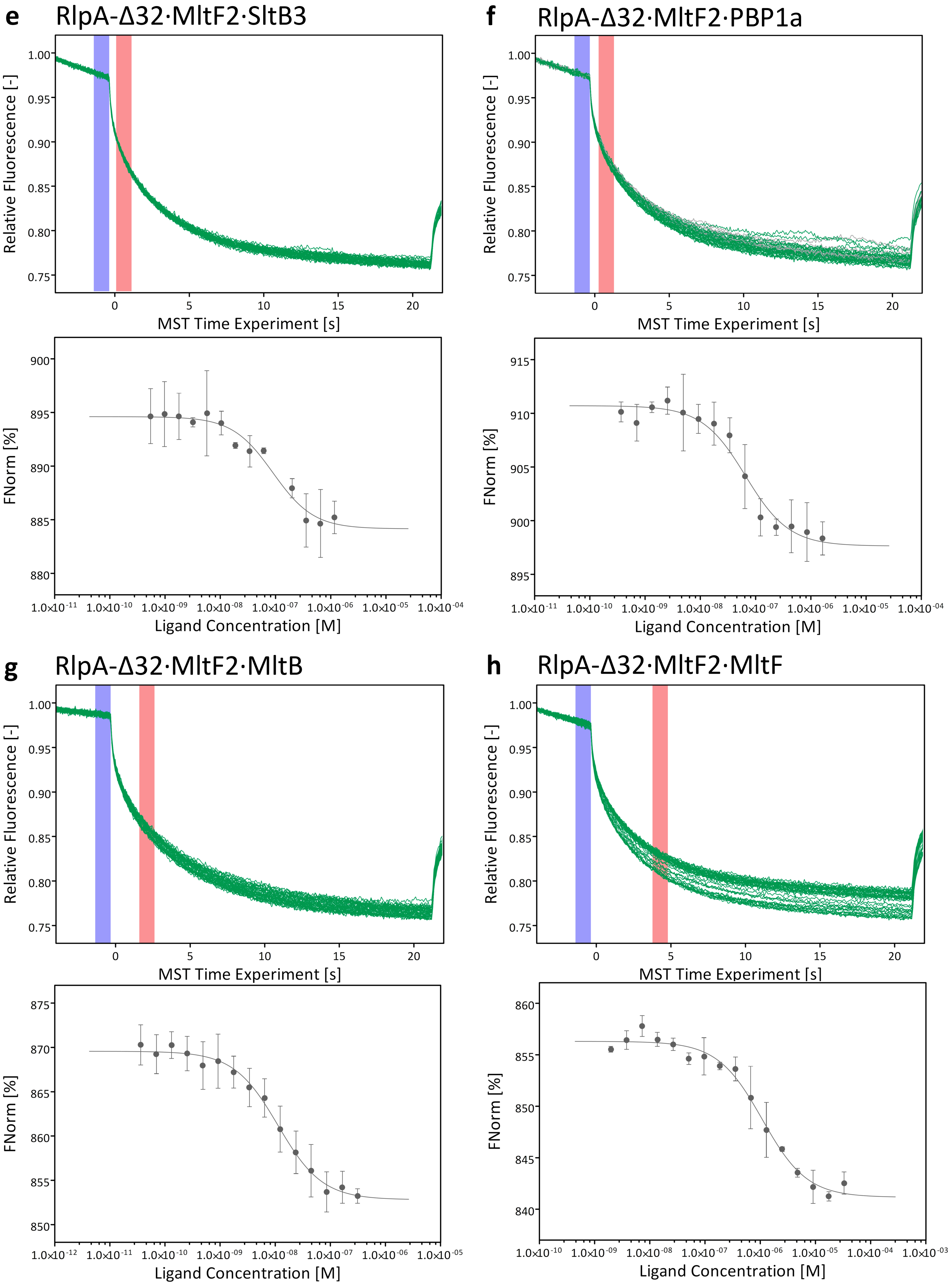
e
RlpA-Δ32·MltF2·SltB3
Relative Fluorescence [-]
MST Time Experiment [s]
FNorm [%]
Ligand Concentration [M]
f
RlpA-Δ32·MltF2·PBP1a
Relative Fluorescence [-]
MST Time Experiment [s]
FNorm [%]
Ligand Concentration [M]
g
RlpA-Δ32·MltF2·MltB
Relative Fluorescence [-]
MST Time Experiment [s]
FNorm [%]
Ligand Concentration [M]
h
RlpA-Δ32·MltF2·MltF
Relative Fluorescence [-]
MST Time Experiment [s]
FNorm [%]
Ligand Concentration [M]

i RlpA-Δ32·MltF2·SltB2

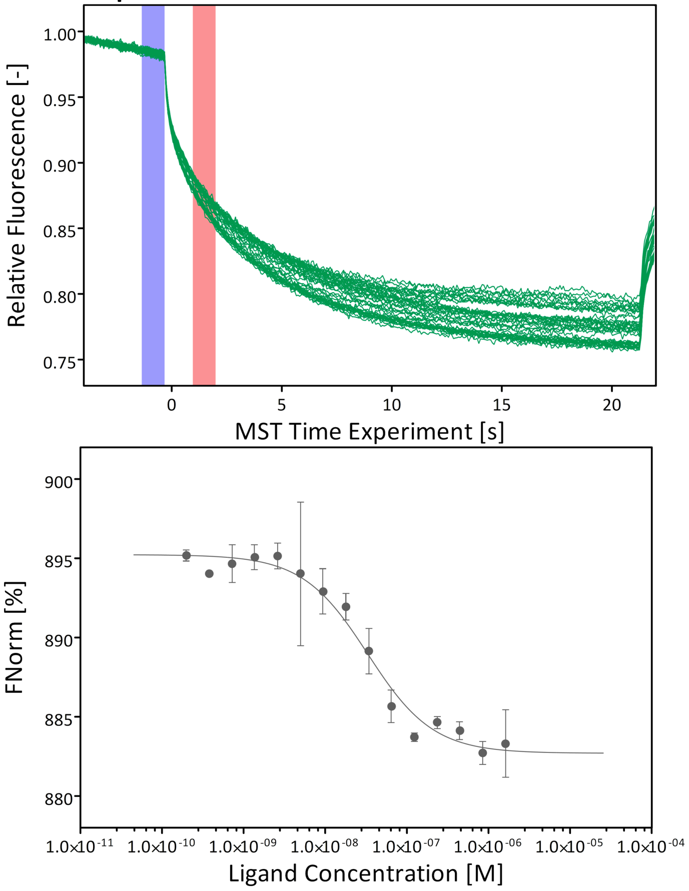

j RlpA-Δ32·MltF2·SltB1

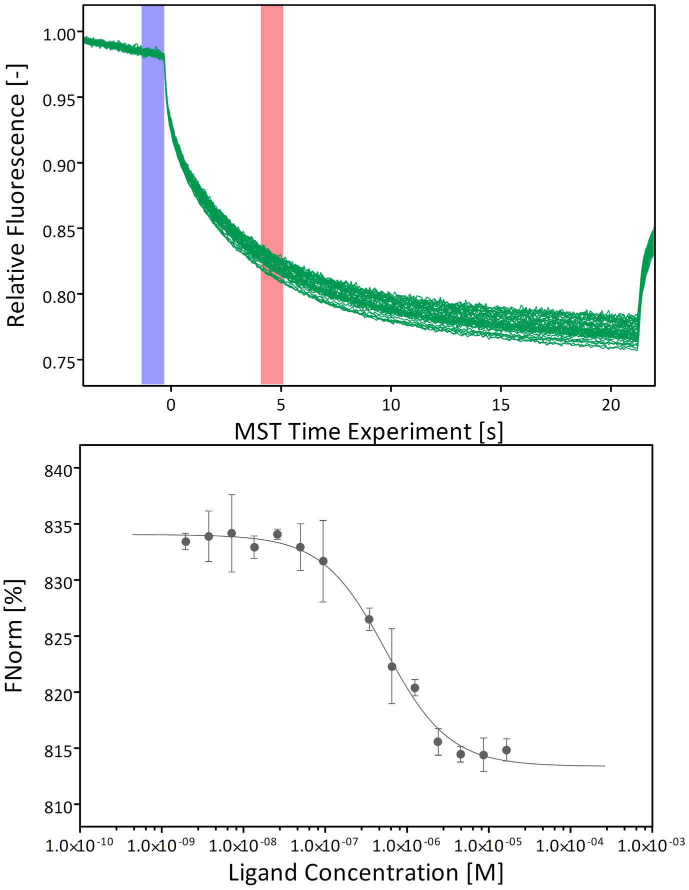

k RlpA-Δ32·PA4063

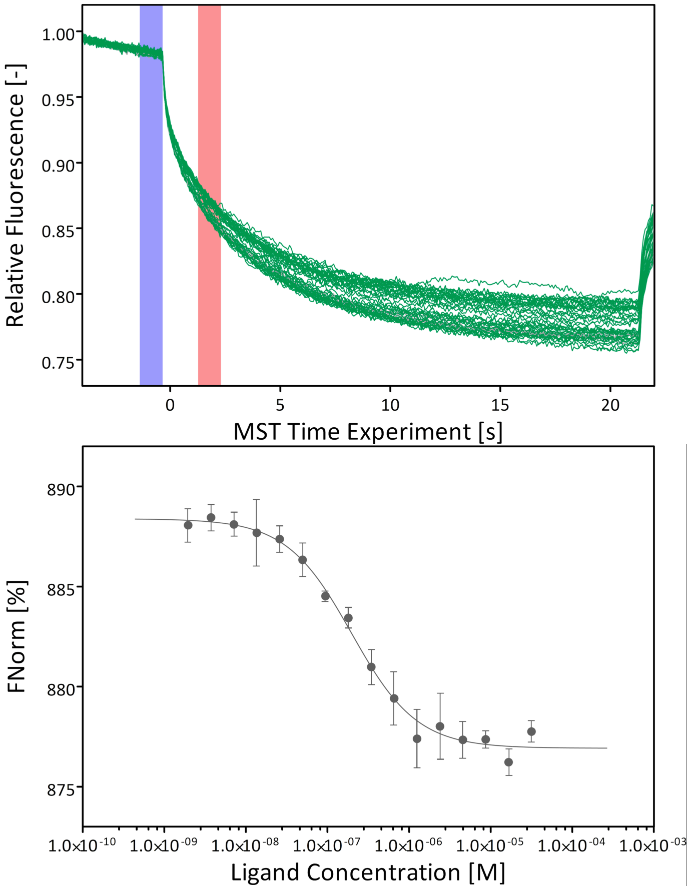

l RlpA-Δ32·MltF2·MltD

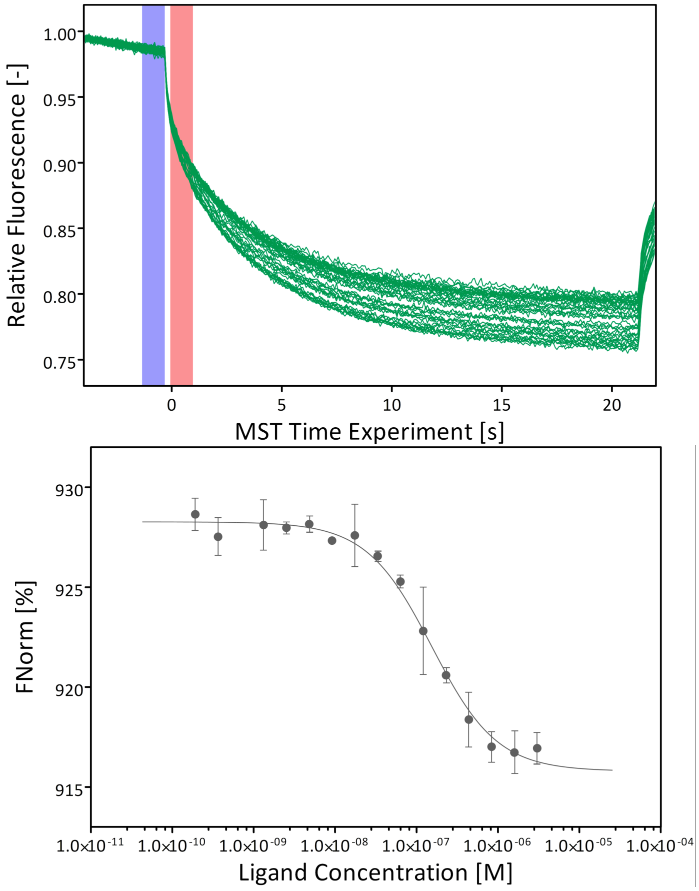

m RlpA-Δ32·MltF2·PilA

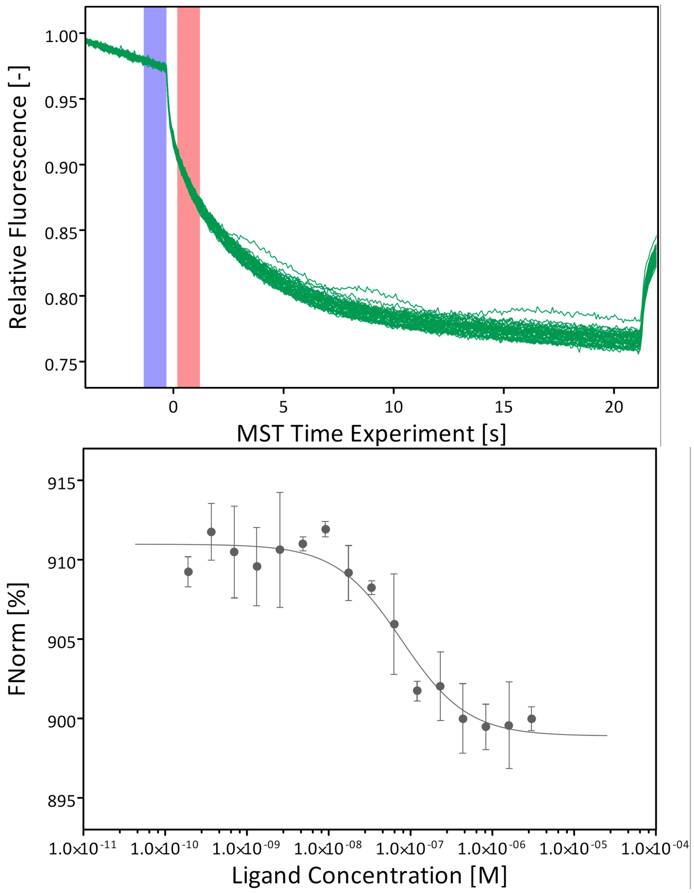

n RlpA-Δ32·MltF2·PilO

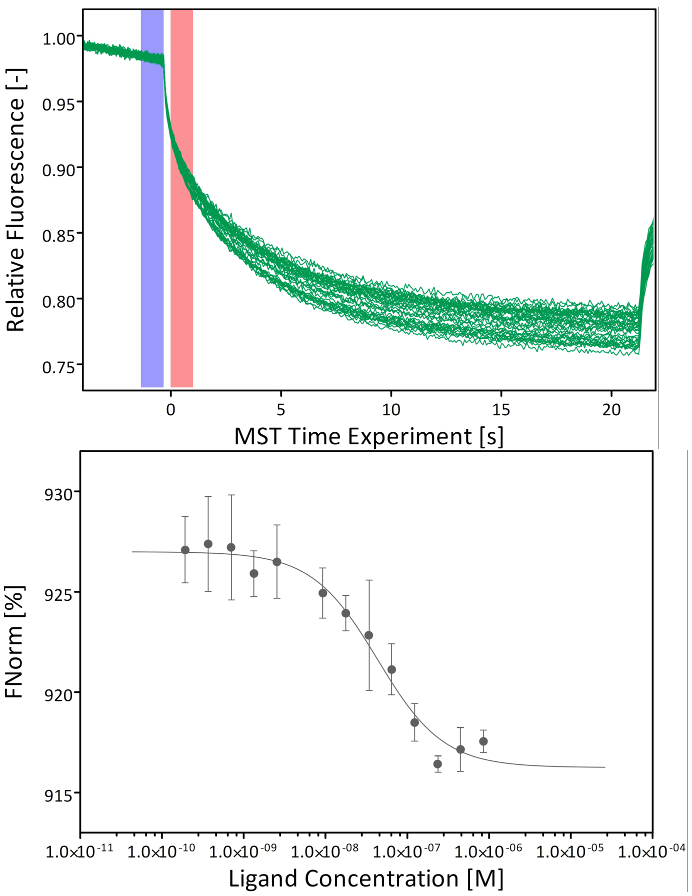

o RlpA-Δ32·MltF2·PBP4

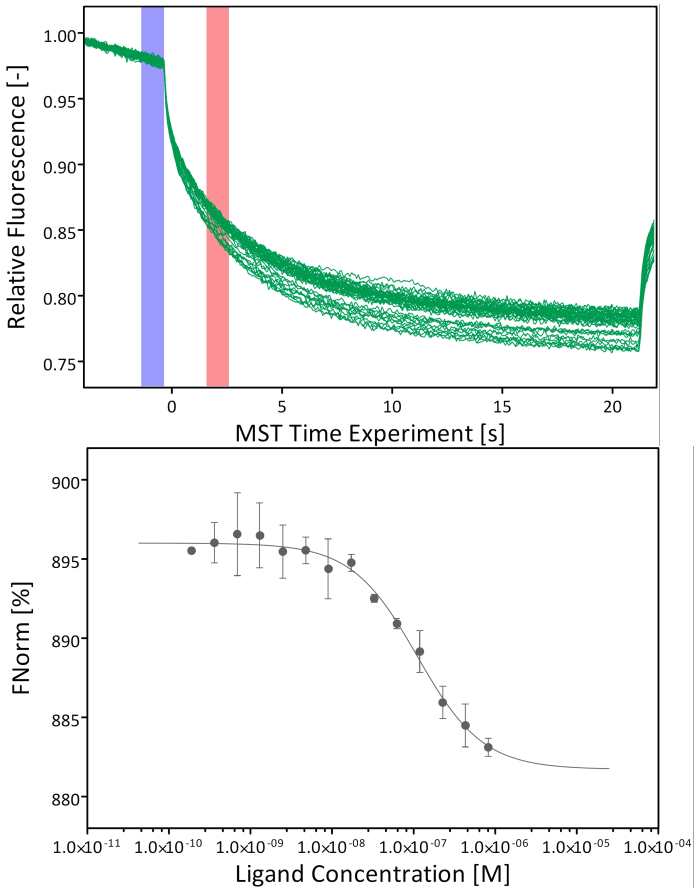

p RlpA-Δ32·MltF2·TypA

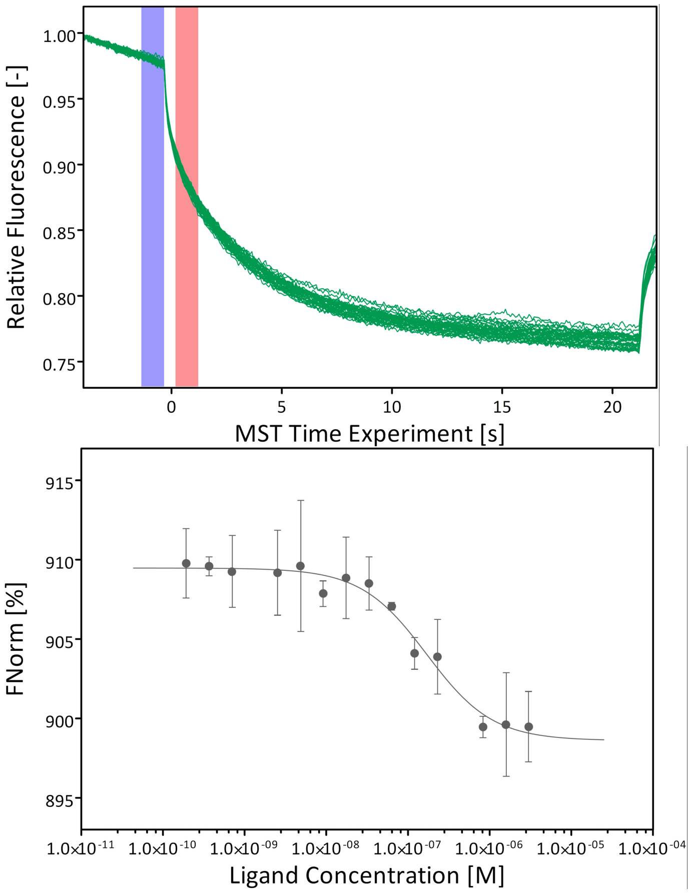

q RlpA-Δ32·MltF2·PBP7

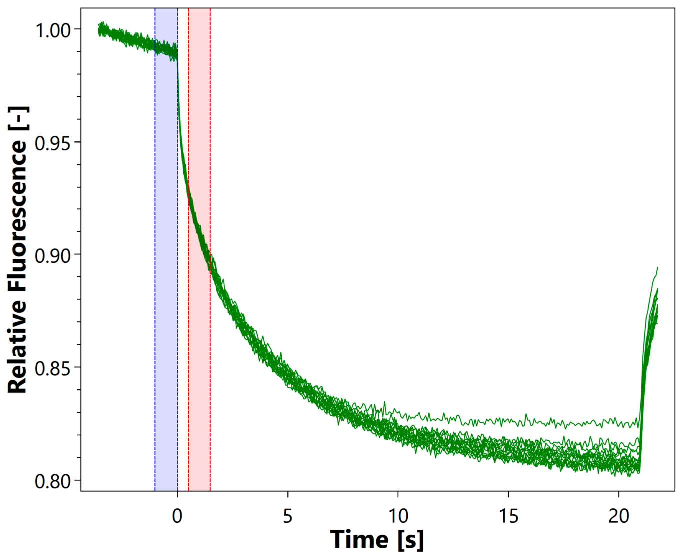

r RlpA-Δ32·MltF2·SlyB

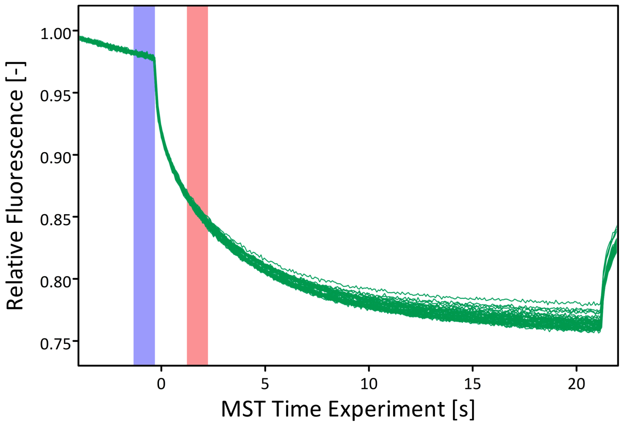

s RlpA-Δ32·MltF2·AlgO

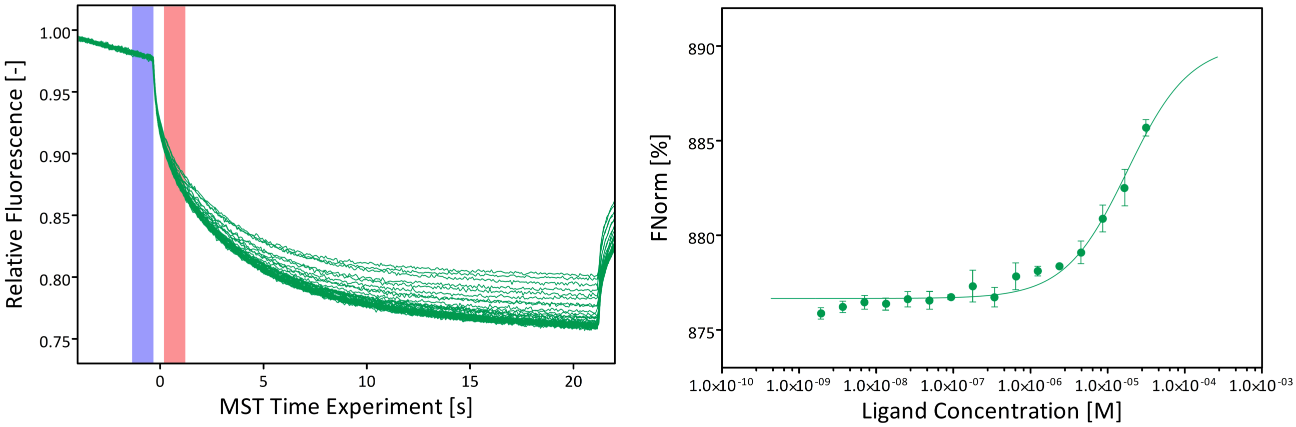

Supplementary Fig. 4. MST traces (top) and dose-response curves (bottom) of tested ternary RlpA-Δ32 combinations. a RlpA-Δ32, MltF2, and PBP1b. **b** RlpA-Δ32, MltF2, and PA2854. **c** RlpA-Δ32, MltF2, and MltA. **d** RlpA-Δ32, MltF2, and LptE. **e** RlpA-Δ32, MltF2, and SltB3. **f** RlpA-Δ32, MltF2, and PBP1a **g** RlpA-Δ32, MltF2, and MltB. **h** RlpA-Δ32, MltF2, and MltF. **i** RlpA-Δ32, MltF2, and SltB2. **j** RlpA-Δ32, MltF2, and SltB1. **k** RlpA-Δ32, MltF2, and PA4063. **l** RlpA-Δ32, MltF2, and MltD. **m** RlpA-Δ32, MltF2, and PilA. **n** RlpA-Δ32, MltF2, and PilO. **o** RlpA-Δ32, MltF2, and PBP4. **p** RlpA-Δ32, MltF2, and TypA. **q** RlpA-Δ32, MltF2, and PBP7; no binding interaction determined within this ternary combination. **r** RlpA-Δ32, MltF2, and SlyB; no binding interaction determined within this ternary combination. **s** RlpA-Δ32, MltF2, AlgO. View Table 1 (right column) for K_D parameters of tested RlpA-Δ32 ternary combinations through MST. Data are presented as means ± S.D. from triplicate experiments.

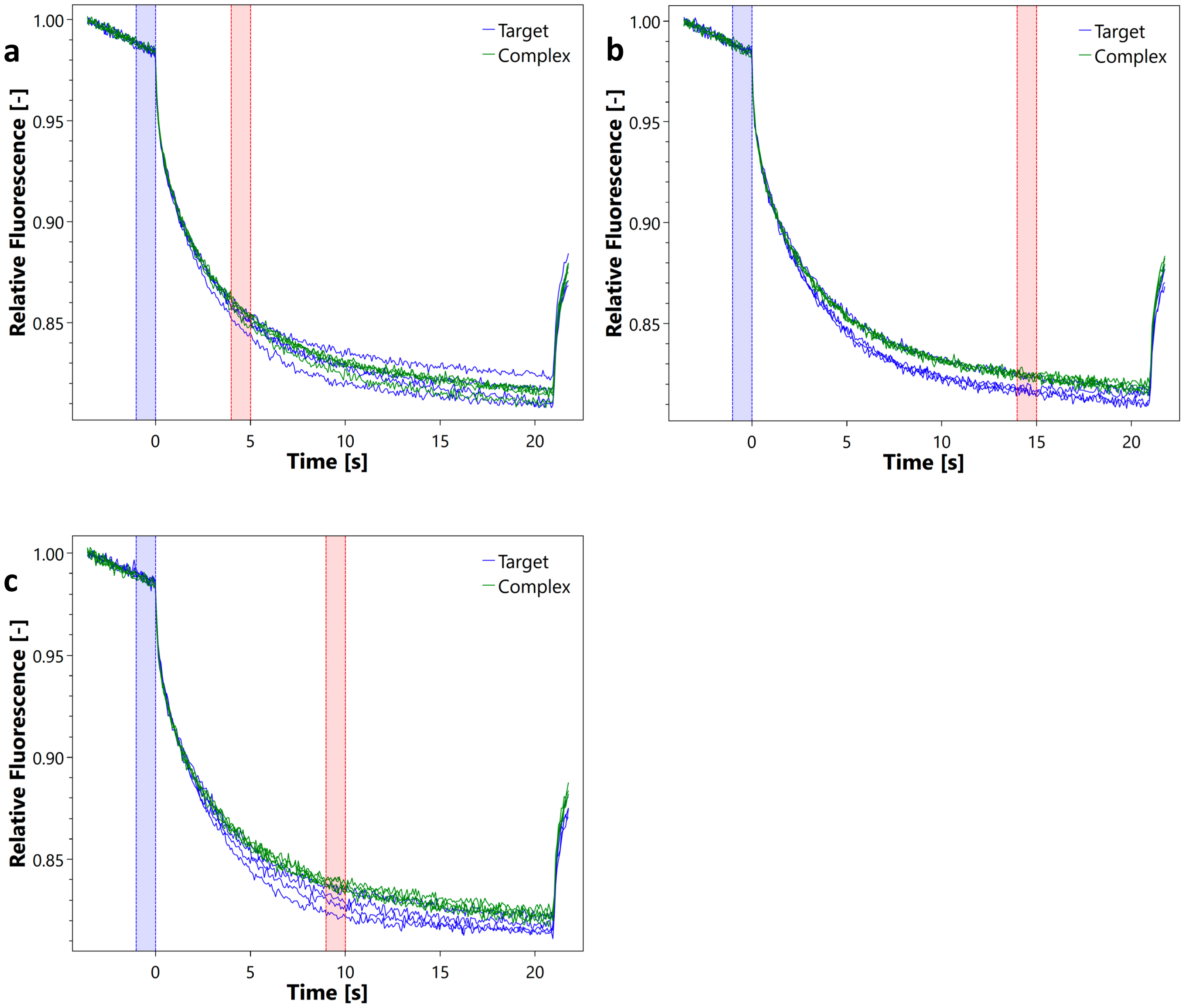

Supplementary Fig. 5. Determination of the availability of binding for presumed negative controls against RlpA-Δ32. **a** RlpA-Δ32 and lysozyme (chicken egg white, Sigma-Aldrich). **b** RlpA-Δ32 and heat-denatured MltF2 (at 120 °C for 10 min prior to analysis). **c** RlpA-Δ32 and BulH. No binding interaction was determined for these tested protein-protein combinations. MST traces are shown since dose-response curve is not available for these analyses.

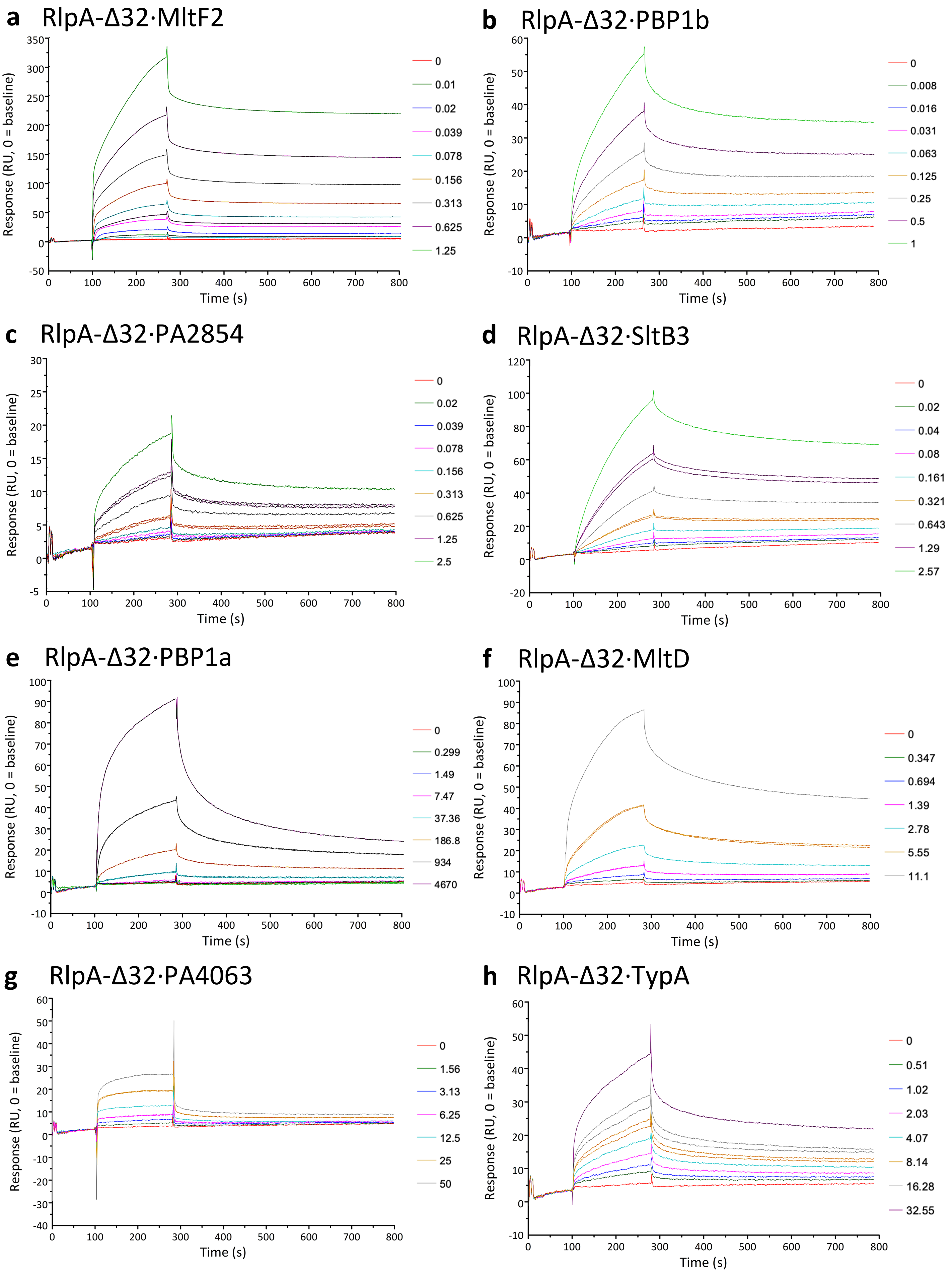

a RlpA-Δ32·MltF2
Response (RU, 0 = baseline)
Time (s)
0
0.01
0.02
0.039
0.078
0.156
0.313
0.625
1.25
b RlpA-Δ32·PBP1b
Response (RU, 0 = baseline)
Time (s)
0
0.008
0.016
0.031
0.063
0.125
0.25
0.5
1
c RlpA-Δ32·PA2854
Response (RU, 0 = baseline)
Time (s)
0
0.02
0.039
0.078
0.156
0.313
0.625
1.25
2.5
d RlpA-Δ32·SltB3
Response (RU, 0 = baseline)
Time (s)
0
0.02
0.04
0.08
0.161
0.321
0.643
1.29
2.57
e RlpA-Δ32·PBP1a
Response (RU, 0 = baseline)
Time (s)
0
0.299
1.49
7.47
37.36
186.8
934
4670
f RlpA-Δ32·MltD
Response (RU, 0 = baseline)
Time (s)
0
0.347
0.694
1.39
2.78
5.55
11.1
g RlpA-Δ32·PA4063
Response (RU, 0 = baseline)
Time (s)
0
1.56
3.13
6.25
12.5
25
50
h RlpA-Δ32·TypA
Response (RU, 0 = baseline)
Time (s)
0
0.51
1.02
2.03
4.07
8.14
16.28
32.55

i RlpA-Δ32·PilO

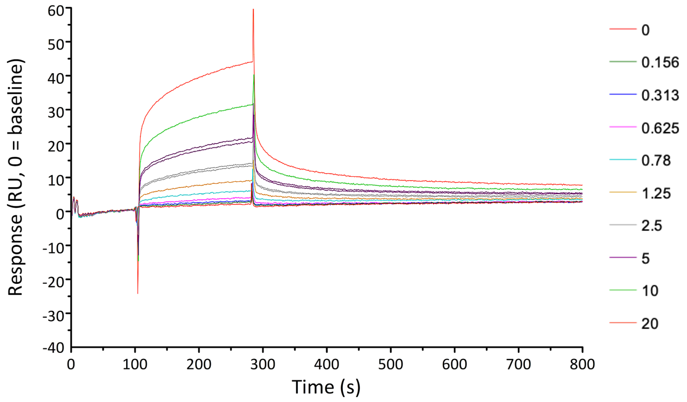

j RlpA-Δ32·AlgO

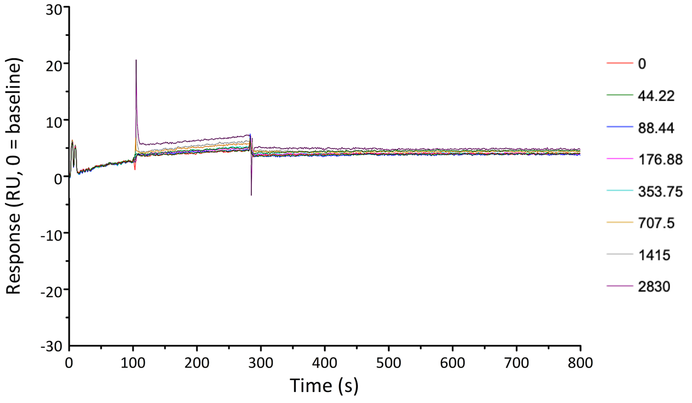

k RlpA-Δ32·PilA

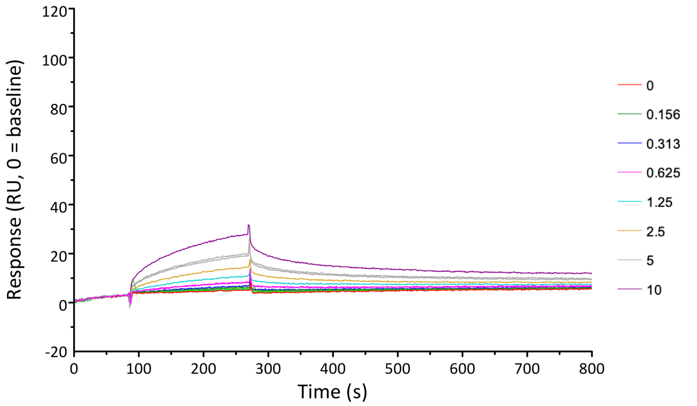

l RlpA-Δ32·SltB2

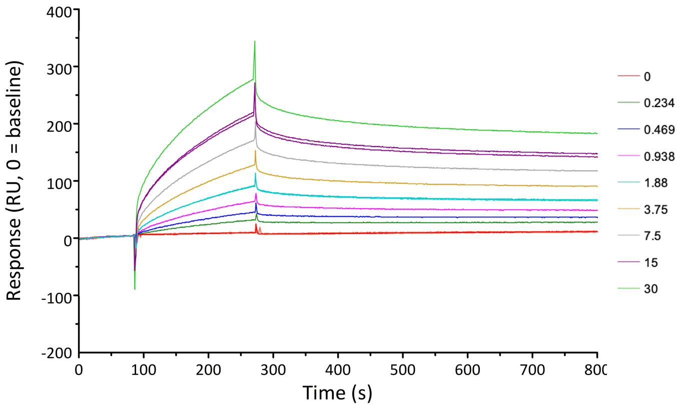

m RlpA-Δ32·MltB

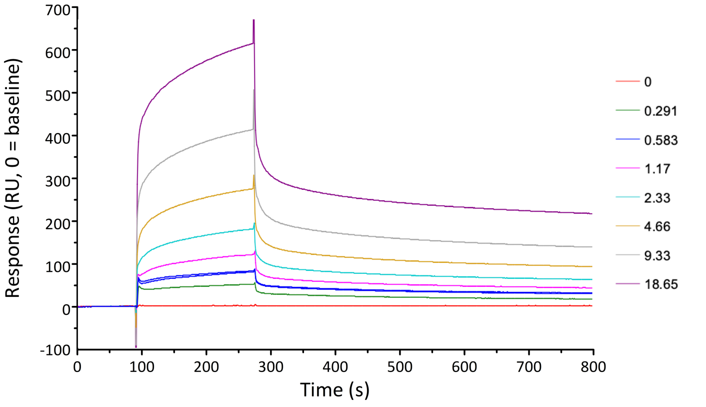

n RlpA-Δ32·PBP7

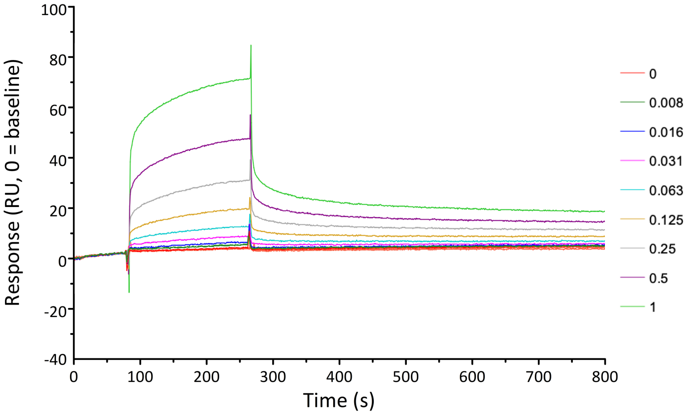

o RlpA-Δ32·PBP4

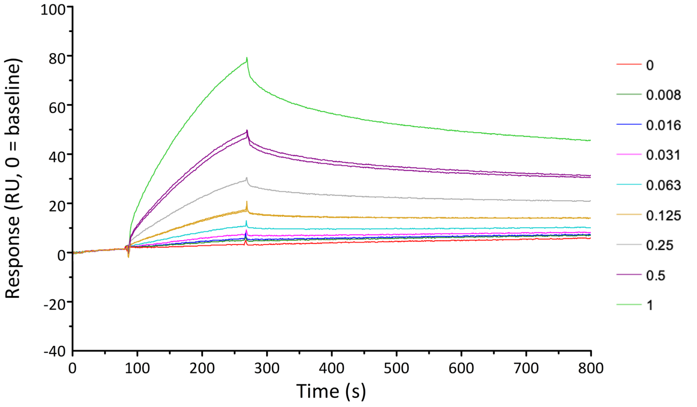

p RlpA-Δ32·SltB1

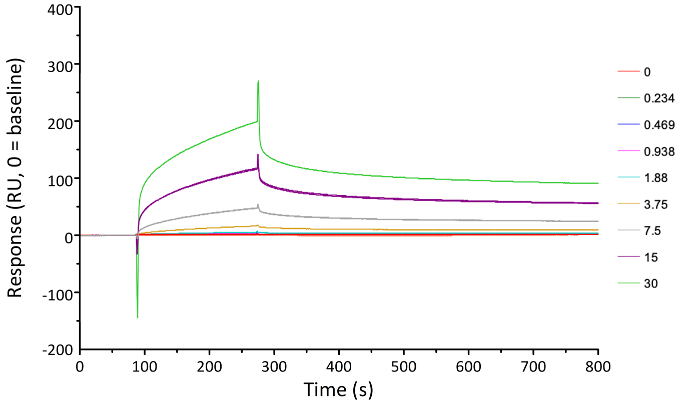

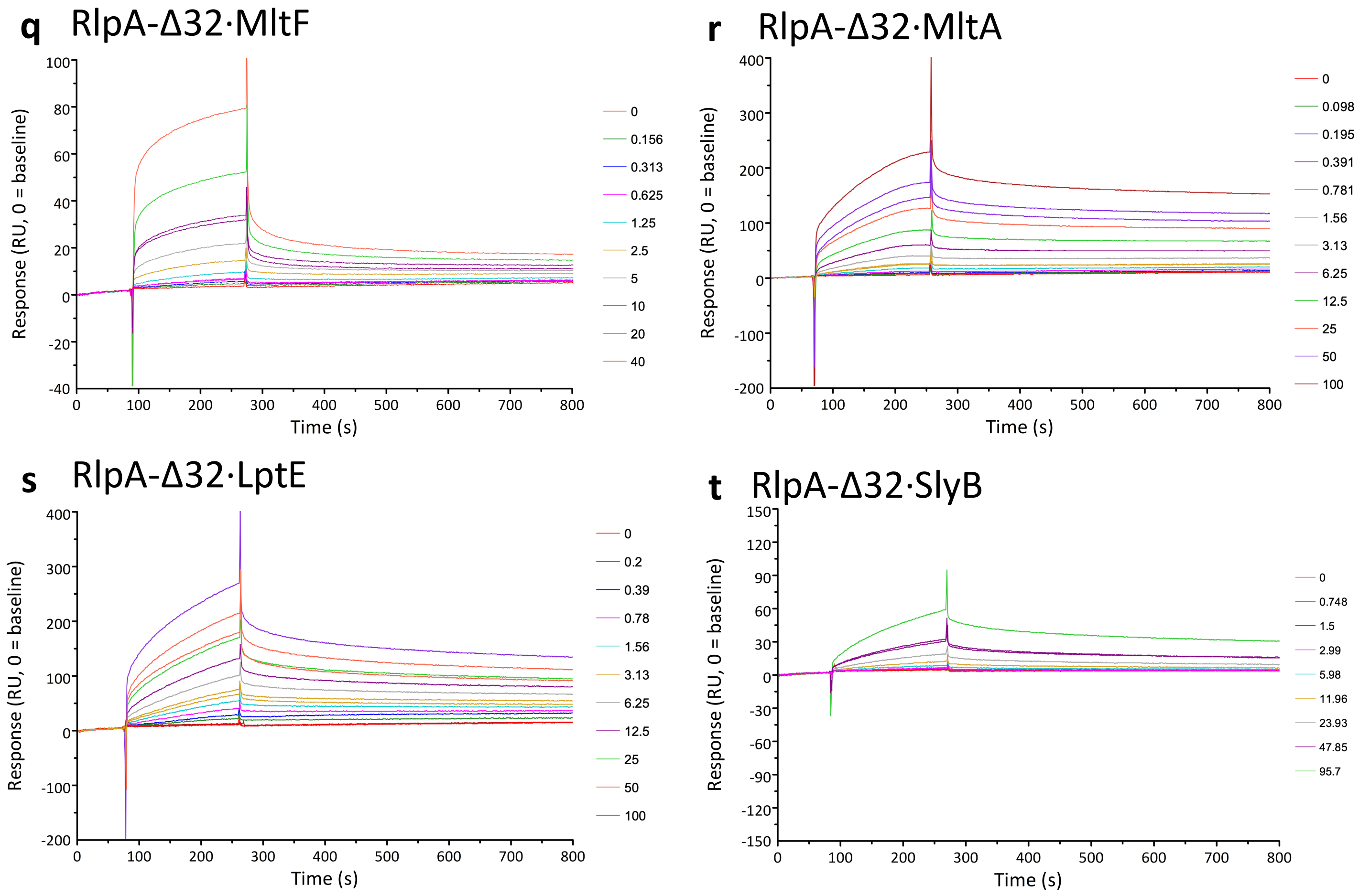

Supplementary Fig. 6. SPR sensorgrams of tested binary RlpA-Δ32 interactions. a RlpA-Δ32 and MltF2 (units are in μM). **b** RlpA-Δ32 and PBP1b (units are in μM). **c** RlpA-Δ32 and PA2854 (units are in μM). **d** RlpA-Δ32 and SltB3 (units are in μM). **e** RlpA-Δ32 and PBP1a (units are in nM). **f** RlpA-Δ32 and MltD (units are in μM). **g** RlpA-Δ32 and PA4063 (units are in μM). **h** RlpA-Δ32 and TypA (units are in μM). **i** RlpA-Δ32 and PilO (units are in μM). **j** RlpA-Δ32 and AlgO; no binding detected (units are in nM). **k** RlpA-Δ32 and PilA (units are in μM). **l** RlpA-Δ32 and SltB2 (units are in μM). **m** RlpA-Δ32 and MltB (units are in μM). **n** RlpA-Δ32 and PBP7 (units are in μM). **o** RlpA-Δ32 and PBP4 (units are in μM). **p** RlpA-Δ32 and SltB1 (units are in μM). **q** RlpA-Δ32 and MltF (units are in μM). (**R**) RlpA-Δ32 and MltA (units are in μM). **s** RlpA-Δ32 and LptE (units are in μM). **t** RlpA-Δ32 and SlyB (units are in μM). All sensorgrams have had their baselines corrected to zero. Any presence of 'spikes' at the beginning of both the association and disassociation phases were eliminated prior to kinetic one-to-one modeling. RU denotes Response Units. All SPR runs were done in triplicate. Each sensorgram under Supplementary Fig. 6 is one of the triplicate SPR runs. View Table 1 for kinetic parameters of tested RlpA-Δ32 binary combinations through SPR.

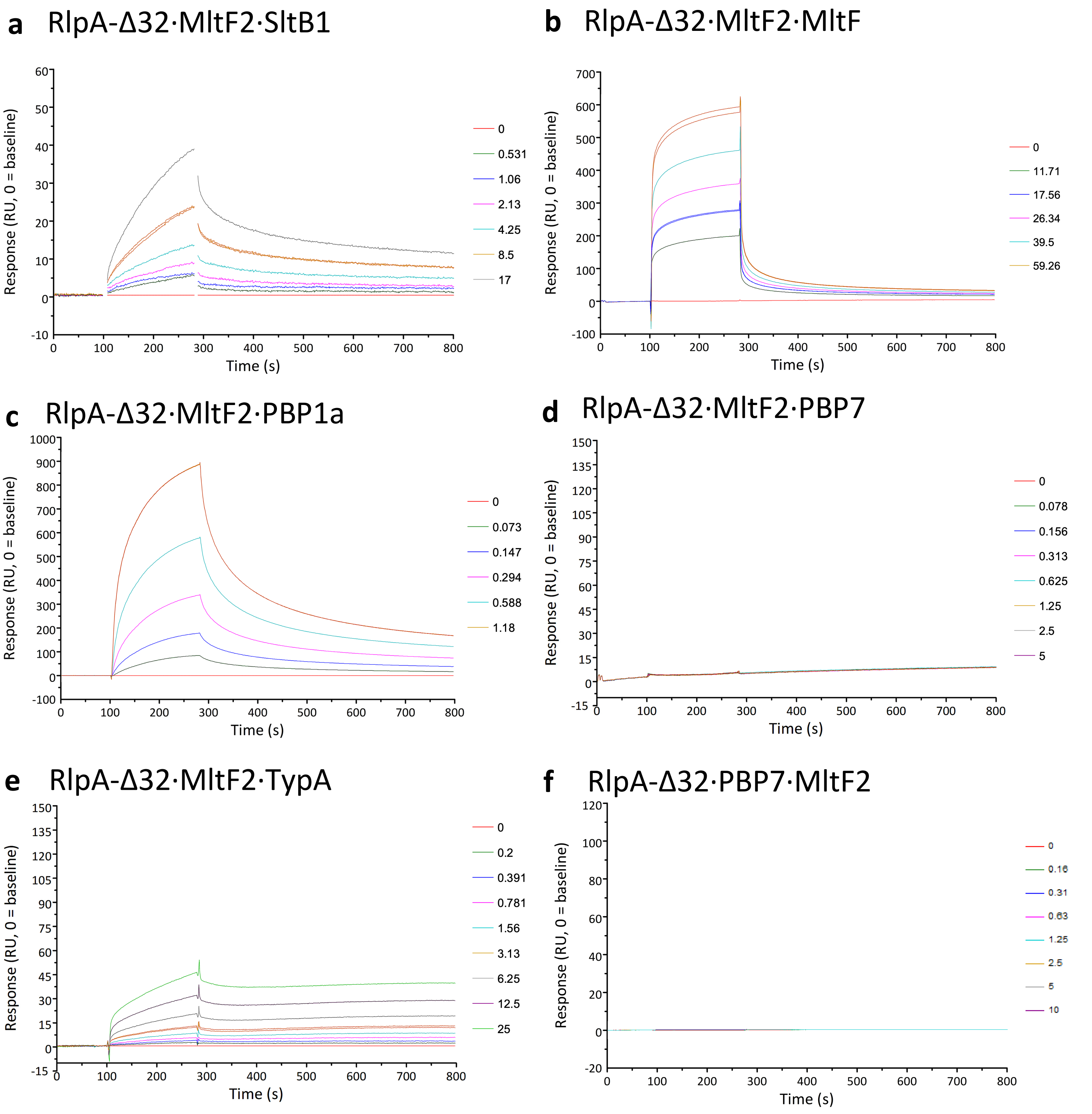

RlpA-Δ32 MltF2	K_D (nM)	10^{-3} k_{on} ($M^{-1} s^{-1}$)	10^{3} k_{off} (s^{-1})
SltB1	N/A	BD[a]	BD[a]
MltF	N/A	BD[a]	BD[a]
PBP1a	280 ± 17	51.7 ± 0.2	4.8 ± 0.1
PBP7	NBD[b]	NBD[b]	NBD[b]
TypA	N/A	BD[a]	BD[a]
**RlpA-Δ32 PBP7**	**K_D (nM)**	**10^{-3} k_{on} ($M^{-1} s^{-1}$)**	**10^{3} k_{off} (s^{-1})**
MltF2	NBD[b]	NBD[b]	NBD[b]

Supplementary Fig. 7. SPR sensorgrams and kinetics of tested ternary RlpA-Δ32 interactions. a–e Sensorgrams have RlpA-Δ32 and MltF2 as ligand, crosslinked to each other prior to any SPR runs or analysis. **a** SltB2 as analyte. **b** MltF as analyte. **c** PBP1a as analyte. **d** PBP7 as analyte. **e** TypA as analyte. **f** sensorgram have RlpA-Δ32 and PBP7 as ligand, crosslinked to each other prior to any SPR run or analysis with MltF2 as analyte. All units are in μM. All sensorgrams have had their baselines corrected to zero. Any presence of 'spikes' at the beginning of both the association and disassociation phases were eliminated prior to kinetic 1:1:1 modeling. RU denotes Response Units. All SPR runs were done in triplicate. Each sensorgram under Supplementary Fig. 7 is one of the triplicate SPR runs. Table displays kinetics from sensorgram fitting. [a]BD denotes "binding detected"; however, kinetic parameters were outside the limitation of the instrument for accurate measurement. [b]NBD for "no binding detected". Data are presented as means ± S.E.M from triplicate experiments.

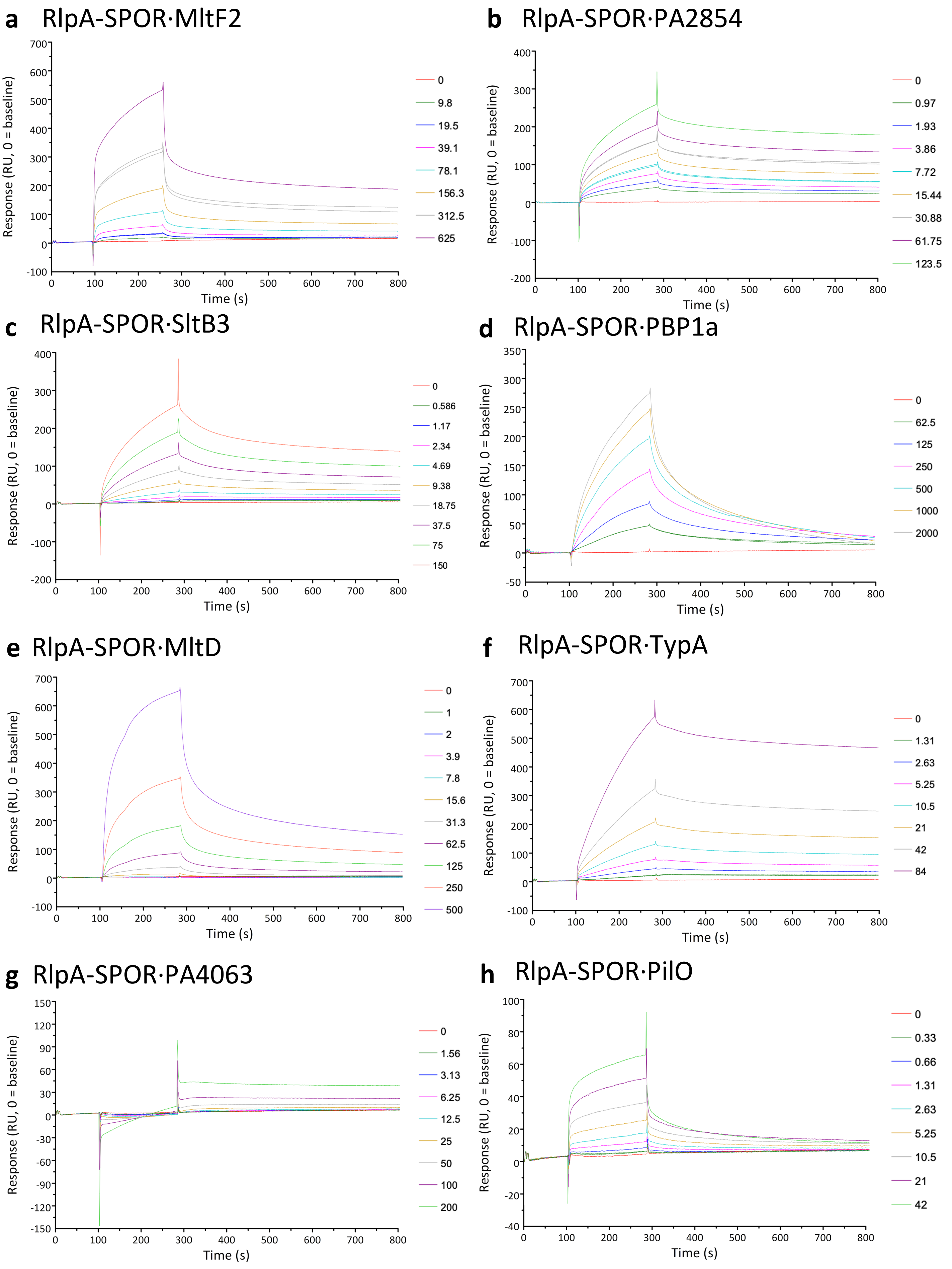
a RlpA-SPOR·MltF2
Response (RU, 0 = baseline)
Time (s)
0
9.8
19.5
39.1
78.1
156.3
312.5
625
b RlpA-SPOR·PA2854
0
0.97
1.93
3.86
7.72
15.44
30.88
61.75
123.5
c RlpA-SPOR·SltB3
0
0.586
1.17
2.34
4.69
9.38
18.75
37.5
75
150
d RlpA-SPOR·PBP1a
0
62.5
125
250
500
1000
2000
e RlpA-SPOR·MltD
0
1
2
3.9
7.8
15.6
31.3
62.5
125
250
500
f RlpA-SPOR·TypA
0
1.31
2.63
5.25
10.5
21
42
84
g RlpA-SPOR·PA4063
0
1.56
3.13
6.25
12.5
25
50
100
200
h RlpA-SPOR·PilO
0
0.33
0.66
1.31
2.63
5.25
10.5
21
42

Supplementary Fig. 8. SPR sensorgrams of tested RlpA-SPOR interactions. a RlpA-SPOR and MltF2 (units are in nM). **b** RlpA-SPOR and PA2854 (units are in μM). **c** RlpA-SPOR and SltB3 (units are in μM). **d** RlpA-SPOR and PBP1a (units are in nM). **e** RlpA-SPOR and MltD (units are in nM). **f** RlpA-SPOR and PA4063 (units are in μM). **g** RlpA-SPOR and TypA (units are in μM). **h** RlpA-SPOR and PilO (units are in μM). All sensorgrams have had their baselines corrected to zero. Any presence of 'spikes' at the beginning of both the association and disassociation phases were eliminated prior to kinetic 1:1 modeling. RU denotes Response Units. All SPR runs were done in triplicate. Each sensorgram under Supplementary Fig. 8 is one of the triplicate SPR runs. View Table 3 for kinetic parameters of tested RlpA-SPOR binary combinations through SPR.

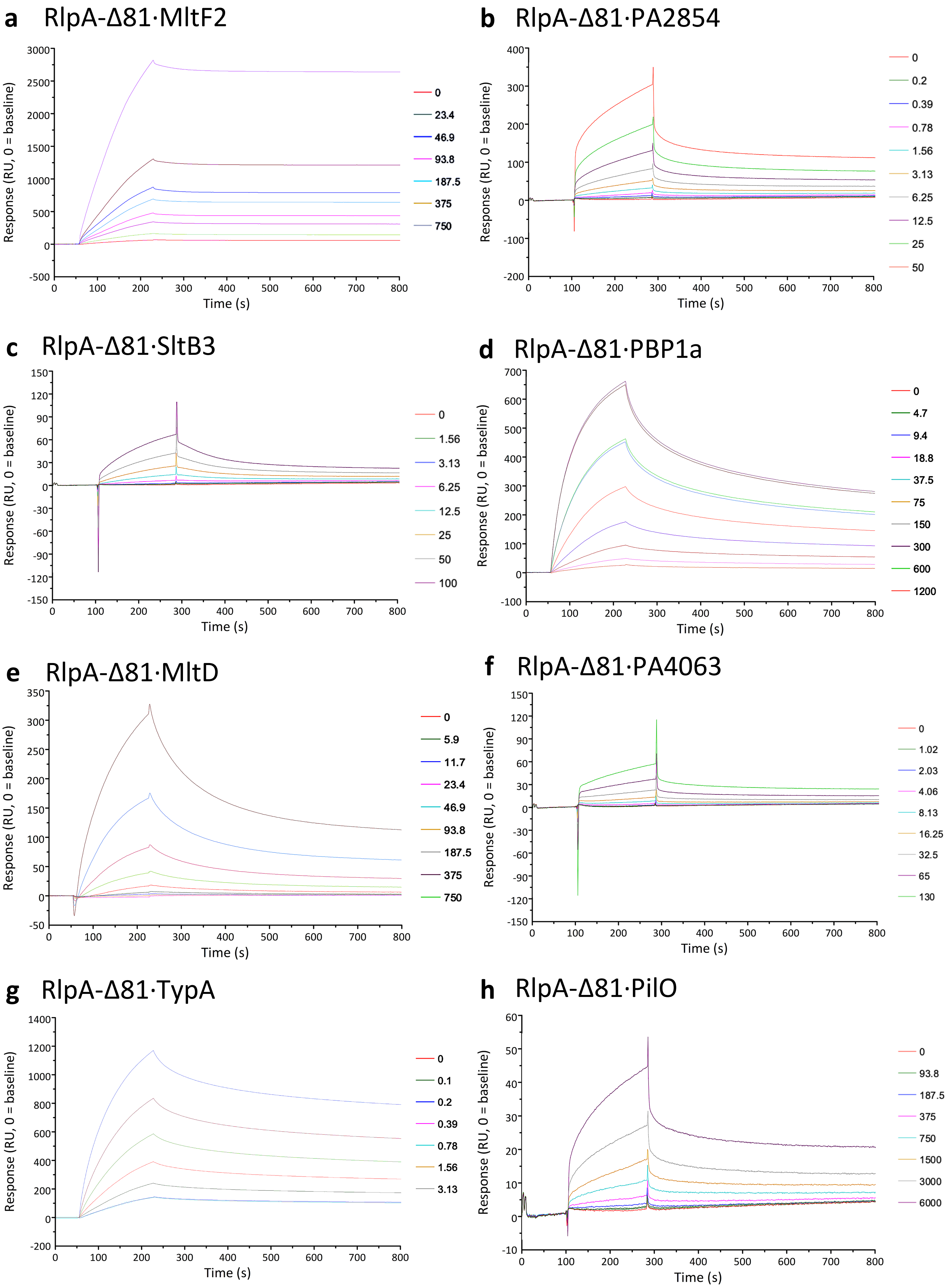
a RlpA-Δ81·MltF2
Response (RU, 0 = baseline)
Time (s)
0
23.4
46.9
93.8
187.5
375
750
b RlpA-Δ81·PA2854
Response (RU, 0 = baseline)
Time (s)
0
0.2
0.39
0.78
1.56
3.13
6.25
12.5
25
50
c RlpA-Δ81·SltB3
Response (RU, 0 = baseline)
Time (s)
0
1.56
3.13
6.25
12.5
25
50
100
d RlpA-Δ81·PBP1a
Response (RU, 0 = baseline)
Time (s)
0
4.7
9.4
18.8
37.5
75
150
300
600
1200
e RlpA-Δ81·MltD
Response (RU, 0 = baseline)
Time (s)
0
5.9
11.7
23.4
46.9
93.8
187.5
375
750
f RlpA-Δ81·PA4063
Response (RU, 0 = baseline)
Time (s)
0
1.02
2.03
4.06
8.13
16.25
32.5
65
130
g RlpA-Δ81·TypA
Response (RU, 0 = baseline)
Time (s)
0
0.1
0.2
0.39
0.78
1.56
3.13
h RlpA-Δ81·PilO
Response (RU, 0 = baseline)
Time (s)
0
93.8
187.5
375
750
1500
3000
6000

Supplementary Fig. 9. SPR sensorgrams of tested RlpA-Δ81 interactions. a RlpA-Δ81 and MltF2 (units are in nM). **b** RlpA-Δ81 and PA2854 (units are in μM). **c** RlpA-Δ81 and SltB3 (units are in μM). **d** RlpA-Δ81 and PBP1a (units are in nM). **e** RlpA-Δ81 and MltD (units are in nM). **f** RlpA-Δ81 and PA4063 (units are in μM). **g** RlpA-Δ81 and TypA (units are in μM). **h** RlpA-Δ81 and PilO (units are in nM). All sensorgrams have had their baselines corrected to zero. Any presence of 'spikes' at the beginning of both the association and disassociation phases were eliminated prior to kinetic 1:1 modeling. RU denotes Response Units. All SPR runs were done in triplicate. Each sensorgram under Supplementary Fig. 9 is one of the triplicate SPR runs. View Table 3 for kinetic parameters of tested RlpA-Δ81 binary combinations tested SPR.

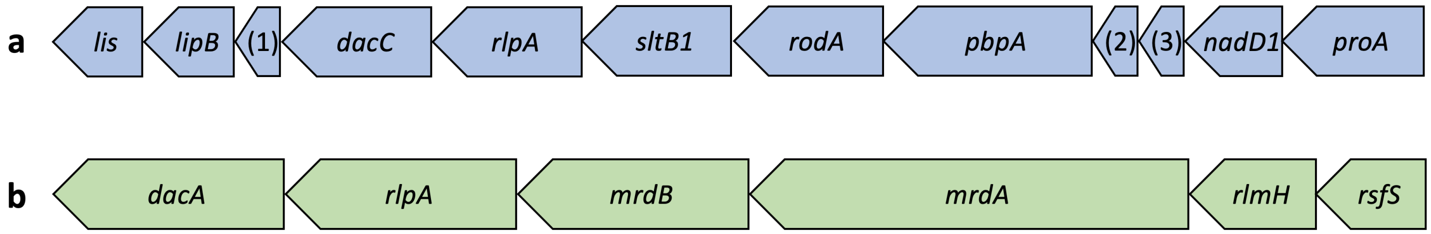

Supplementary Fig. 10. RlpA operons in *Escherichia coli* and *Pseudomonas aeruginosa.* **a** RlpA operon in *P. aeruginosa* PAO1, a subset of 12 genes. Operon is from the Pseudomonas Genome Database. **b** RlpA operon in *E. coli* K-12, a subset of six genes. Operon is from the EcoCyc Database. In **a**, *lis* encodes for lipoic acid synthetase LipA. *lipB* encodes for lipoate biosynthesis protein B LipB. '(1)' denotes the PA3998 gene. PA3998 encodes for the uncharacterized and conserved hypothetical protein YbeD. *dacC* encodes for D-ala-D-ala endopeptidase pencillin-binding protein PBP5. *rlpA* encodes for rare lipoprotein A RlpA (*E. coli* homolog *rlpA*). *sltB1* encodes for soluble lytic transglycosylase SltB1. *rodA* encodes for rod shape-determining protein RodA (*E. coli* homolog is *mrdB*). *pbpA* encodes for transpeptidase pencillin-binding protein PBP2 (*E. coli* homolog is *mrdA*). '(2)' denotes PA4004 gene. PA4004 encodes for encodes for 23S mRNA methyltransferase RlmH (*E. coli* homolog *rlmH*). '(3)' denotes PA4005 gene. PA4005 encodes for the ribosomal silencing factor RsfS (*E. coli* homolog is *rsfS*). *nadD1* encodes for nicotinic acid mononucleotide adenylyltransferase NadD1 (synonym of the protein name is YbeN). *proA* encodes for the glutamate-semialdehyde dehydrogenase ProA. In **b**, *dacA* encodes for the D-ala-D-ala carboxypeptidase pencillin-binding protein PBP1a. The synonym of the gene name is *pbpA.* The *P. aeruginosa* homolog is *ponA*. *rlpA* encodes for the rare lipoprotein A RlpA (*P. aeruginosa* homolog is *rlpA*). *mrdB* encodes for rod shape-determinating protein RodA (*P. aeruginosa* homolog is *rodA*). *mrdA* encodes for transpeptidase pencillin-binding protein PBP2. The *P. aeruginosa* homolog is *pbpA*. *rlmH* encodes for 23S mRNA methyltransferase RlmH (*P. aeruginosa* homolog is PA4004). *rsfS* encodes for ribosomal silencing factor RsfS (*P. aeruginosa* homolog is PA4005). The length of each gene in **a** and **b** is representative of their base pair length within their own operons, but are not scaled for interspecies comparisons in gene length.

Supplementary Fig. 11. Primary structures of SPOR-domain containing proteins within *Pseudomonas aeruginosa* PAO1.

Protein	Amino Acid Sequence
**RlpA (PA4000)**	MSKRVRSSLILPAVCGLGLAAVLLSSCSSKAPQQPARQAGISGPGDYSRPHRDGAPWWDVDVSRIPDAVPMPHNGSVKANPYTVLGKTYYPMNDARAYRMVGTASWYGTKFHGQATANGETYDLYGMTAAHKTLPLPSYVRVTNLDNGKSVIVRVNDRGPFYSDRVIDLSFAAAKKLGYAETGTARVKVEGIDPVQWWAQRGRPAPMVLAQPKQAVAQAAPAAAQTQAVAMAQPIETYTPPPAQHAAAVLPVQIDSKKNASLPADGLYLQVGAFANPDAAELLKAKLSGVTAAPVFISSVVRNQQILHRVRLGPIGSADEVSRTQDSIRVANLGQPTLVRPD
**FtsN (PA5052)**	MAKKKPAPKRGASRYQAPAAKNGVPGWVWLVAGLAIGGFIMFLMKLEPGRKDVQRERPDAQRPAAVQGKQPQQNGQAQQPAQTQTPAQAKPKYEFYTLLPESVVVPPEAVPEKAPPPPTPAELAKADEARAKAALAGQVPPPLPKAVVAASTQFFLQAGSFRKQSDADRVRAQIILLGQSVNVEAGNVRDETWYRVMVGPFNSRDQLSQAQKTLSSNGFSNLLLQQRKAR
**PA3110**	MALLERGLKQRIVGALVLIALAVIFLPMLFTREDESRQVVVEAPPRPQSPAMPSVEVQPTEVPELQPGEEGIAPEIVEEGSPAAAGQPSQPIGGLPATPPATQPPAQAQAPAASLPPSQPQPPAAPPSPPPAEKRLDANNLPQSWSVQLASLSNRARAEELQKTLRSQGYNAYIRSFDGMNRVFVGPVIQRAEADRLRDQLSKQQKLNGFVVRFQPERG
**DedD (PA4278)**	MRWFFLFLLALNVFYYVWHQQQSPLRAKEIAPLELYKDGQKNILLLAESNLASRARSGSQVPAPSVPPAAEESIASESACLYLGGGGEEADARRLRQRLLGLDIEAEVEARGEMSVDYWVYLPPLASREAALRQLKELQARNIDSYLIGEGVLANGISLGMFSARDSAESAQVRLKTAGYEAELKELPRGQRDFWVRVAPGSRRLVDEQLLQELARDFKGLQHQIMSCKGVASP
**DamX (PA5037)**	MTSLHADEAFLGHYQFSHDPFAPRVPGFKFFPAQRKPVLGQLHHLARYSQLLLLVTGPLGSGKTLLRQALVASTNKDAVLSVVISARTAADETSLLRQVAQGLSINQASLEAILTKVAQLAITGQDVYLMVDDAEQLQDSALEVLLLLASGTNEGRLHVFLFGEPSLLPRLEVFSEGEERFHAIELQPYSEEETRDYLAQRLEGAGQGIELISNDLLVDIHEQSEGWPGAINQVARDALIEAMLANRGAARKATGGSFNLPKKHLVILAVVAIGVIAAWFMQGKSKPEAPQTASTELSMNGATPAQAQQPGSGPAVEFNGSSQPLPLPLVGESQPVIREPLAQAAGQGGDDEGGLPSAAVPPTVSSSAPPVTPLANNGVTPMHPVPPAPTEPTAPAATPTPTQTPAPAAPVASAPASKPAPAPAPAKPAASKPATTAAAKPAPAPAAKPASGGGAGSQWYRNQSGGNFALQILGTGSEANAQAFIRQQGGGDFRYFKKTLQGKPFYVVTYGSFPNRAAALAAVKKLPSKVQAGKPWPRTFASIQQEIGQAH

All sequences were retrieved from The Pseudomonas Genome Database (Cystic Fibrosis Foundation, Therapeutics). The following SPOR proteins PA5052 {Lowe, MM, 2004; Vollmer, Mbio, 2020}, PA4278 {Gerding, De Boer, JB, 2009; Vollmer, Mbio, 2020}, and PA5037 {Weiss, Biochem, 2009; Vollmer, Mbio, 2020} have been annotated from the following references, respectively. The absence of annotation for PA3110 is for being a SPOR protein of unknown function. The SPOR domain is highlighted in purple lettering.

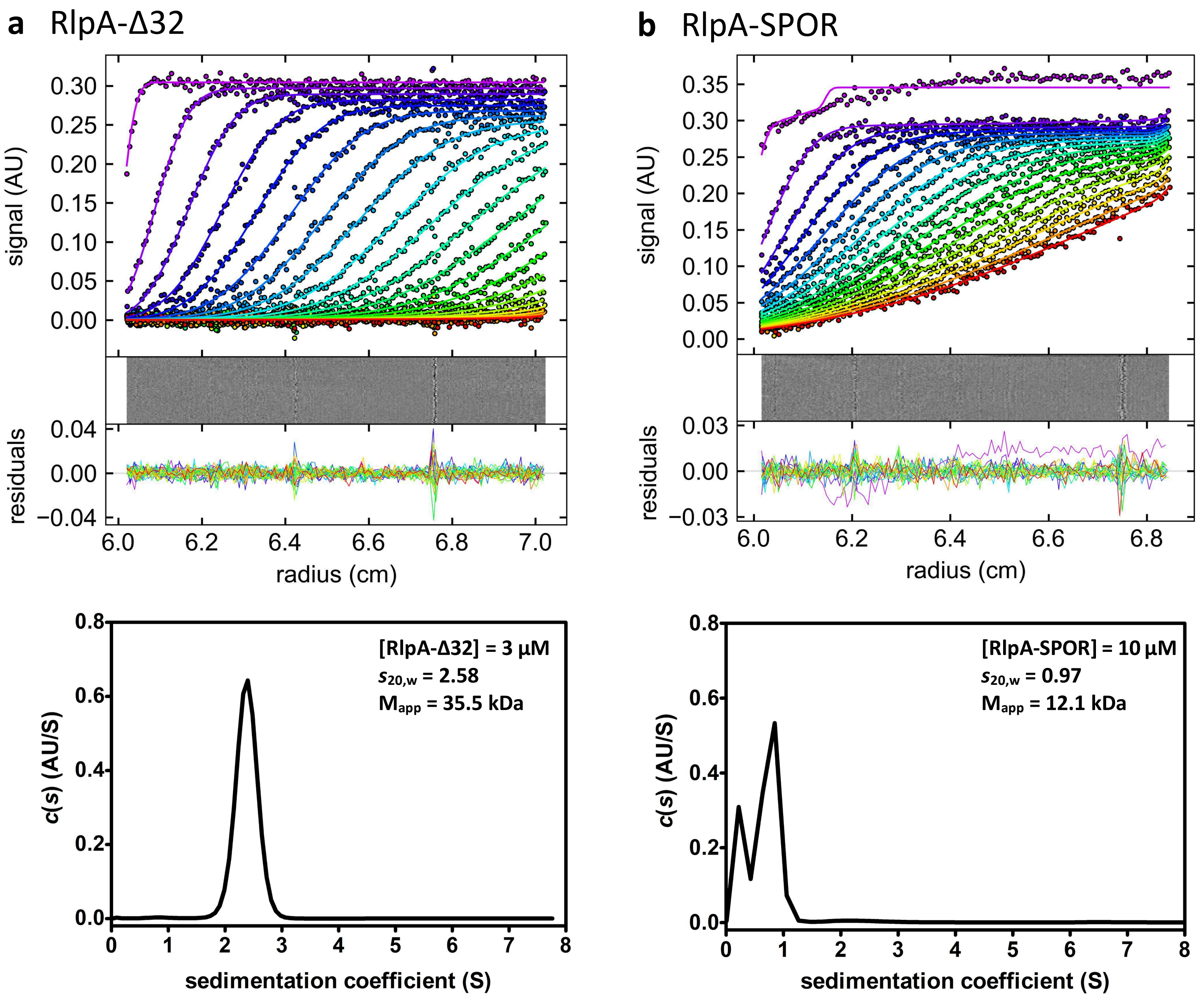

Supplementary Fig. 12. AUC c(*s*) analyses on the monomeric state of the RlpA-constructs. Sedimentation velocity (SV) analysis of RlpA-Δ32 (MW: 35875 Da) and RlpA-SPOR (MW: 11249 Da). **a** Analysis of 3 μM protein. The experimentally determined *s* value transformed to $s_{20,w}$ for the primary peak was 2.58 and an apparent mass of 35.5 kDa. **b** analysis of 10 μM protein. The experimentally determined *s* value transformed to $s_{20,w}$ for the primary peak was 0.97 and an apparent mass of 12.1 Da. Each experiment confirms that the RlpA constructs are monomers in solution.
