# Supplementary material for: In vitro studies of the protein-interaction network of cell-wall lytic transglycosylase RlpA of Pseudomonas aeruginosa
Source: Commun Biol. 2022 Nov 30;5:1314. doi: 10.1038/s42003-022-04230-x (PMC9712689; doi:10.1038/s42003-022-04230-x)
Supplement: Supplementary file 2 — Description of Additional Supplementary Files [file 42003_2022_4230_MOESM2_ESM.pdf]

Description of Additional Supplementary Files

File name: Supplementary Data 1

Description: The source data behind graphs in the paper
