# Supplementary material for: In vitro studies of the protein-interaction network of cell-wall lytic transglycosylase RlpA of Pseudomonas aeruginosa
Source: Commun Biol. 2022 Nov 30;5:1314. doi: 10.1038/s42003-022-04230-x (PMC9712689; doi:10.1038/s42003-022-04230-x)
Supplement: Supplementary file 4 — Reporting Summary [file 42003_2022_4230_MOESM4_ESM.pdf]

nature portfolio

Corresponding author(s): Shahriar Mobashery

Last updated by author(s): Nov 4, 2022

Reporting Summary

Nature Portfolio wishes to improve the reproducibility of the work that we publish. This form provides structure for consistency and transparency in reporting. For further information on Nature Portfolio policies, see our Editorial Policies and the Editorial Policy Checklist.

Statistics

For all statistical analyses, confirm that the following items are present in the figure legend, table legend, main text, or Methods section.

n/a	Confirmed	
☐	☒	The exact sample size (*n*) for each experimental group/condition, given as a discrete number and unit of measurement
☐	☒	A statement on whether measurements were taken from distinct samples or whether the same sample was measured repeatedly
☐	☒	The statistical test(s) used AND whether they are one- or two-sided<br>*Only common tests should be described solely by name; describe more complex techniques in the Methods section.*
☒	☐	A description of all covariates tested
☒	☐	A description of any assumptions or corrections, such as tests of normality and adjustment for multiple comparisons
☒	☐	A full description of the statistical parameters including central tendency (e.g. means) or other basic estimates (e.g. regression coefficient) AND variation (e.g. standard deviation) or associated estimates of uncertainty (e.g. confidence intervals)
☒	☐	For null hypothesis testing, the test statistic (e.g. *F*, *t*, *r*) with confidence intervals, effect sizes, degrees of freedom and *P* value noted<br>*Give P values as exact values whenever suitable.*
☒	☐	For Bayesian analysis, information on the choice of priors and Markov chain Monte Carlo settings
☒	☐	For hierarchical and complex designs, identification of the appropriate level for tests and full reporting of outcomes
☒	☐	Estimates of effect sizes (e.g. Cohen's *d*, Pearson's *r*), indicating how they were calculated

Our web collection on statistics for biologists contains articles on many of the points above.

Software and code

Policy information about availability of computer code

Data collection	RAW files collected from the LT-pulldowns followed by proteomics experiments were done through MaxQuant and quantified using Label-Free Quantification (MaxLFQ). NanoTemper MO Control was used for MST data collection. Biacore-Cytiva X100 Control software was used for SPR data collection. Malvern MicroCal PEAQ-ITC Contorl software was used for ITC data collection. SEDNTERP was used to calculate buffer density and sample viscosity for AUC trials.
Data analysis	The Pseudomonas FASTA database was obtained from the publicly available Pseudomonas Genome Database (the Cystic Fibrosis Foundation, Therapeutics) for proteomic analysis on the LT-pulldown experiments. NanoTemper Affinity Analysis was used for MST data analysis. Cytiva BIAevaluation software was used for SPR data analysis. GraphPad Prism 5 was used for ITC data analysis. AUC data was analyzed using the c(s) distribution and c(M) distribution models in Sedfit, and data was plotted using GUSSI.

For manuscripts utilizing custom algorithms or software that are central to the research but not yet described in published literature, software must be made available to editors and reviewers. We strongly encourage code deposition in a community repository (e.g. GitHub). See the Nature Portfolio guidelines for submitting code & software for further information.

Data

Policy information about availability of data

All manuscripts must include a data availability statement. This statement should provide the following information, where applicable:

- Accession codes, unique identifiers, or web links for publicly available datasets
- A description of any restrictions on data availability
- For clinical datasets or third party data, please ensure that the statement adheres to our policy

The datasets generated from the LT-pulldown coupled with proteomic analysis experiments in the present study are publicly available through the MassIVE/ProteomeExchange data repository (http://mchampion-nas.esc.nd.edu:5000/sharing/zcDQDUJQb Password PA01#2022). Before publication, all data was archived in the Shahriar Mobashery Laboratory. There were no restrictions for any materials used in this study.

Human research participants

Policy information about studies involving human research participants and Sex and Gender in Research.

Reporting on sex and gender	N/A
Population characteristics	N/A
Recruitment	N/A
Ethics oversight	N/A

Note that full information on the approval of the study protocol must also be provided in the manuscript.

Field-specific reporting

Please select the one below that is the best fit for your research. If you are not sure, read the appropriate sections before making your selection.

☒ Life sciences ☐ Behavioural & social sciences ☐ Ecological, evolutionary & environmental sciences

For a reference copy of the document with all sections, see nature.com/documents/nr-reporting-summary-flat.pdf

Life sciences study design

All studies must disclose on these points even when the disclosure is negative.

Sample size	No sample size calculations were performed.
Data exclusions	No data was excluded.
Replication	All trials and methodology were completed in triplicate, as stated within the manuscript.
Randomization	No randomization was necessary.
Blinding	Blinding was not necessary.

Reporting for specific materials, systems and methods

We require information from authors about some types of materials, experimental systems and methods used in many studies. Here, indicate whether each material, system or method listed is relevant to your study. If you are not sure if a list item applies to your research, read the appropriate section before selecting a response.

Materials & experimental systems

n/a	Involved in the study
☒	☐ Antibodies
☒	☐ Eukaryotic cell lines
☒	☐ Palaeontology and archaeology
☒	☐ Animals and other organisms
☒	☐ Clinical data
☒	☐ Dual use research of concern

Methods

n/a	Involved in the study
☒	☐ ChIP-seq
☒	☐ Flow cytometry
☒	☐ MRI-based neuroimaging
